# Supplementary material for: PI3Kδ coordinates transcriptional, chromatin, and metabolic changes to promote effector CD8+ T cells at the expense of central memory
Source: Cell Rep. Author manuscript; Available in PMC 2021 Nov 11. (PMC8582080; doi:10.1016/j.celrep.2021.109804)
Supplement: 1 [file NIHMS1747909-supplement-1.pdf]

Cell Reports, Volume *37*

Supplemental information

PI3Kδ coordinates transcriptional, chromatin, and metabolic changes to promote effector $CD8^+$ T cells at the expense of central memory

Jennifer L. Cannons, Alejandro V. Villarino, Senta M. Kapnick, Silvia Preite, Han-Yu Shih, Julio Gomez-Rodriguez, Zenia Kaul, Hirofumi Shibata, Julie M. Reilley, Bonnie Huang, Robin Handon, Ian T. McBain, Selamawit Gossa, Tuoqi Wu, Helen C. Su, Dorian B. McGavern, John J. O'Shea, Peter J. McGuire, Gulbu Uzel, and Pamela L. Schwartzberg

Supplemental Figure 1

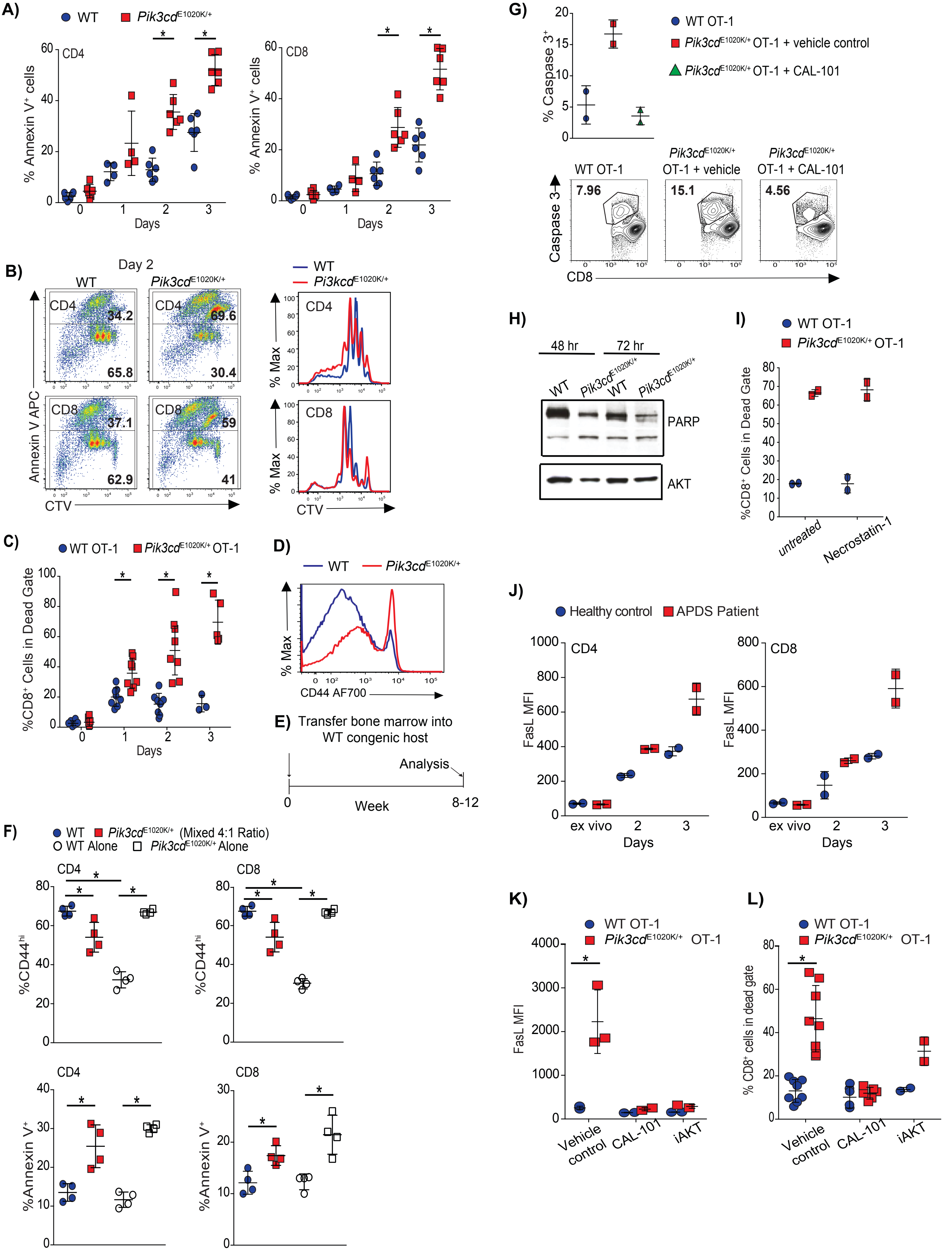

Supplemental Figures

Figure S1. *Pik3cd*$^{E1020K/+}$ T cells reveal pronounced FasL-mediated cell death. Related to Figure 1.

(A-B) CTV labeled WT and *Pik3cd*$^{E1020K/+}$ CD4^{+} (left panel) and CD8^{+} (right panel) T cells stimulated with anti-CD3 plus anti-CD28 and evaluated for A) Annexin-V staining, B) representative flow plot (left panel), histogram of viable cells (right panel) (n=6). (C) Peptide stimulated OT-1 cells were evaluated for cell death by viability stain (n=3-6). (D) Flow cytometric analysis: CD44 expression on viable CD8^{+} T cells from WT and *Pik3cd*$^{E1020K/+}$ mice (mice aged 10 weeks). (E-F) Recipients received WT or *Pik3cd*$^{E1020K/+}$ bone marrow, or for mixed chimeras, recipients received bone marrow from WT and *Pik3cd*$^{E1020K/+}$ (4:1 ratio). E) Experimental outline. F) Percentage of CD44^{hi} CD4^{+} or CD8^{+} T cells (top panels), percentage of Annexin-V^{+} CD4^{+} or CD8^{+} T cells (lower panels). (G) OT-1 cells stimulated with peptide +/- of p110δ inhibitor, CAL-101, and evaluated for caspase-3 activity, 48hr. Bottom panel: representative flow plot, n=2. (H) WT and *Pik3cd*$^{E1020K/+}$ T cells stimulated with anti-CD3 plus anti-CD28. Representative western blot for PARP and AKT (n=2). (I) Cell death analysis of OT-1 cells stimulated with peptide +/- Necrostatin-1 (n=2). (J) Surface FasL MFI: *ex vivo* and anti-CD3 plus anti-CD28 stimulated CD4^{+} (left panel) and CD8^{+} (right panel) T cells from healthy controls and ADPS patients (n=2). (K-L) OT-1 cells evaluated post peptide stimulation in the presence of p110δ inhibitor CAL-101 or iAKT (n=2-5). K) FasL MFI from viable cells and L) Cell death analysis, (n=2-3). Graphs show mean ± SEM *$p<0.05$.

Supplemental Figure 2

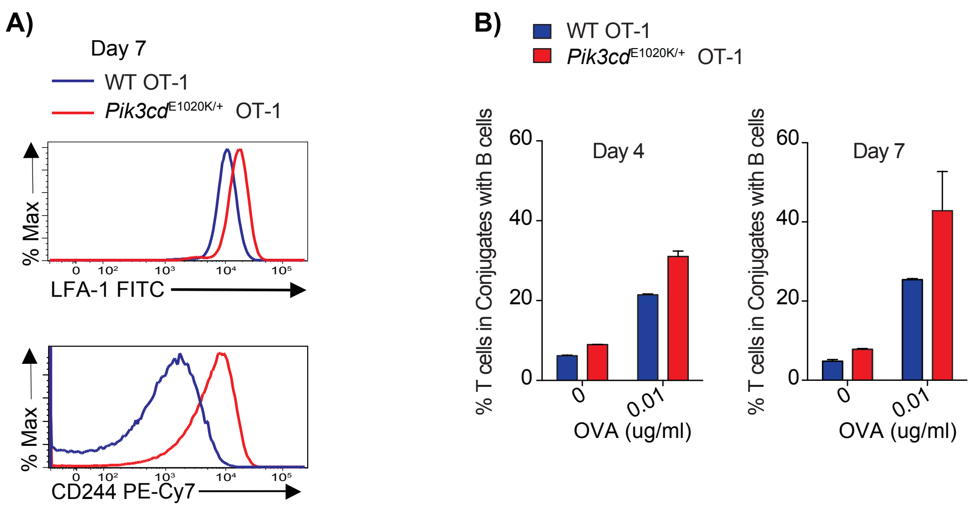

Figure S2. ***Pik3cd*$^{E1020K/+}$ OT-1 CD8^{+} T cells display an early and enhanced effector phenotype.**

Related to Figure 2.

(A) OT-1 T cells were stimulated with peptide for 3 days and expanded in exogenous IL-2. Flow cytometric analysis of LFA-1 (top panel) and CD244 (2B4) (bottom panel) expression on day 7 (n=3, representative histogram shown). (B) WT and *Pik3cd*$^{E1020K/+}$ OT-1 cells were stimulated with peptide for 3 days and expanded in exogenous IL-2 for the indicated times. Conjugate efficiency with LPS-activated B cells pulsed with $OVA_{257-264}$ (n=2). Graphs show mean ± SEM $p<0.05$.

Supplemental Figure 3

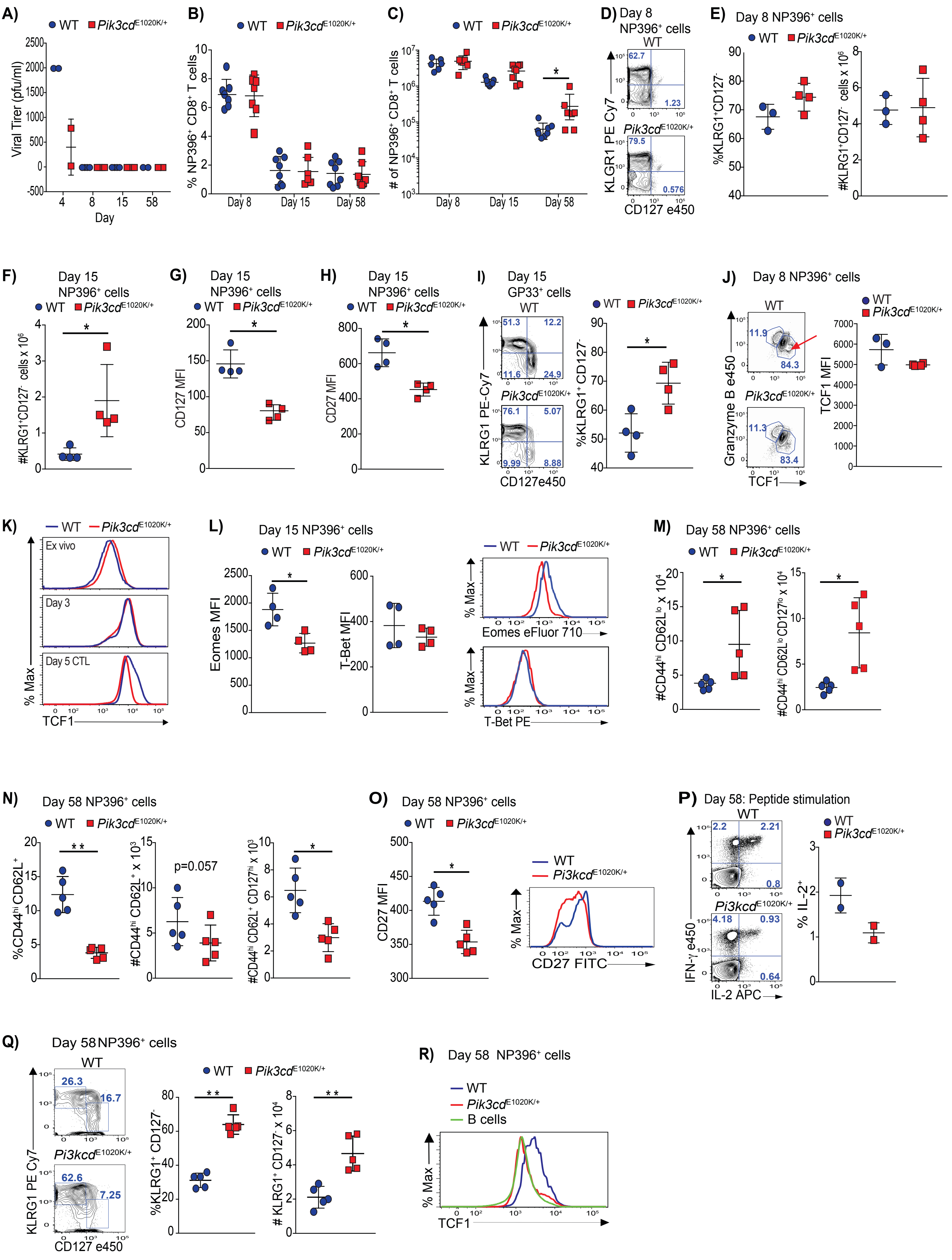

Figure S3. ***Pik3cd*$^{E1020K/+}$ mice exhibit impaired development of T_{CM} populations. Related to Figure 3.**

Mice were infected with LCMV Armstrong strain (n=2, 2-5 mice/group/time point). (A) Viral plaque assay from liver samples. B) Percentage and C) number of NP396-specific $CD8^+$ T cells on day 8, 15 and 58 post-infection. (D-E) Day 8 NP396-specific $CD8^+$ T cells. D) Analysis of KLGR1 and CD127 expression, representative flow plot, E) %$KLRG1^+CD127^-$ cells (left panel), number of $KLRG1^+CD127^-$ cells (right panel). (F-H) Day 15 NP396-specific $CD8^+$ T cells, F) number of $KLRG1^+CD127^-$ cells. G) CD127 MFI and H) CD27 MFI. (I) Day 15 GP33-specific $CD8^+$ T cells. Analysis of KLGR1 and CD127 expression, representative flow plot (left panel). %$KLRG1^+CD127^-$ cells (right panel). (J) Day 8 NP396-specific $CD8^+$ T cells. Analysis of Granzyme B and TCF1 expression, representative flow plot, left panel. Arrow points to $TCF1^{hi}$ population. TCF1 MFI, right panel. (K) OT-1 T cells were stimulated with peptide for 3 days and subsequently cultured with IL-2. Viable OT-1 cells were examined for TCF1 expression (n=3, representative histograms). (L) Day 15 NP396-specific $CD8^+$ T cells, Eomes MFI (left panel), T-Bet MFI (middle panel)), representative histograms, right panel. (M-N) Day 58 NP396-specific $CD8^+$ T cells. Cell numbers of $CD44^{hi}CD62L^{lo}$ (total T_{EM}) and $CD44^{hi}CD62L^{lo}CD127^{lo}$ (LLEC), populations; percentages and cell numbers of $CD44^{hi}CD62L^+$ and cell numbers of $CD44^{hi}CD62L^+CD127^{hi}$ T_{CM} cells. (O) Day 58 NP396-specific $CD8^+$ T cells, CD27 MFI (left panel), representative histogram (right panel). (P) Day 58, splenocytes stimulated with peptide and evaluated for IFN-γ and IL-2 production. Left panel, flow plot and right panel, % $IL\text{-}2^+$ cells. (Q) Day 58 NP396-specific $CD8^+$ T cells evaluated for KLRG1 and CD127 (left panel, representative flow plot), right panels: percentage and cells numbers of $KLRG1^+CD127^-$ populations. (R) Day 58 NP396-specific $CD8^+$ T cells, representative TCF1 histogram. Graphs show mean ± SEM *p<0.05, **p<0.01.

Supplemental Figure 4

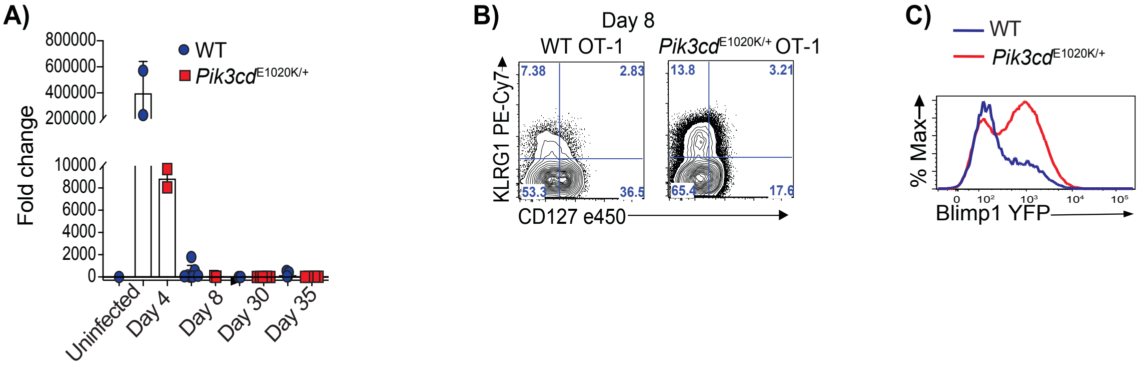

Figure S4. *Pik3cd*$^{E1020K/+}$ mice exhibit altered responses to influenza infection. Related to Figure 3.

(A) Lung influenza viral titer following X31 infection and PR8 challenge (n=2, 3-5 mice per time point). (B) WT and *Pik3cd*$^{E1020K/+}$ OT-1 cells were transferred into congenic hosts, subsequently infected with influenza X31-OVA (n=2, 3 mice/genotype/time point). Analysis of CD127 and KLRG1 expression on transferred OT-1 cells, day 8 (representative flow plot). (C) OT-1 Blimp1-YFP CD8^{+} T cells were transferred into CD45.1/2 host, subsequently infected with aerosolized X31-OVA influenza. Blimp1-YPF expression assessed day 8 post-infection (n=3 mice, representative example). Graphs show mean ± SEM.

Supplemental Figure 5

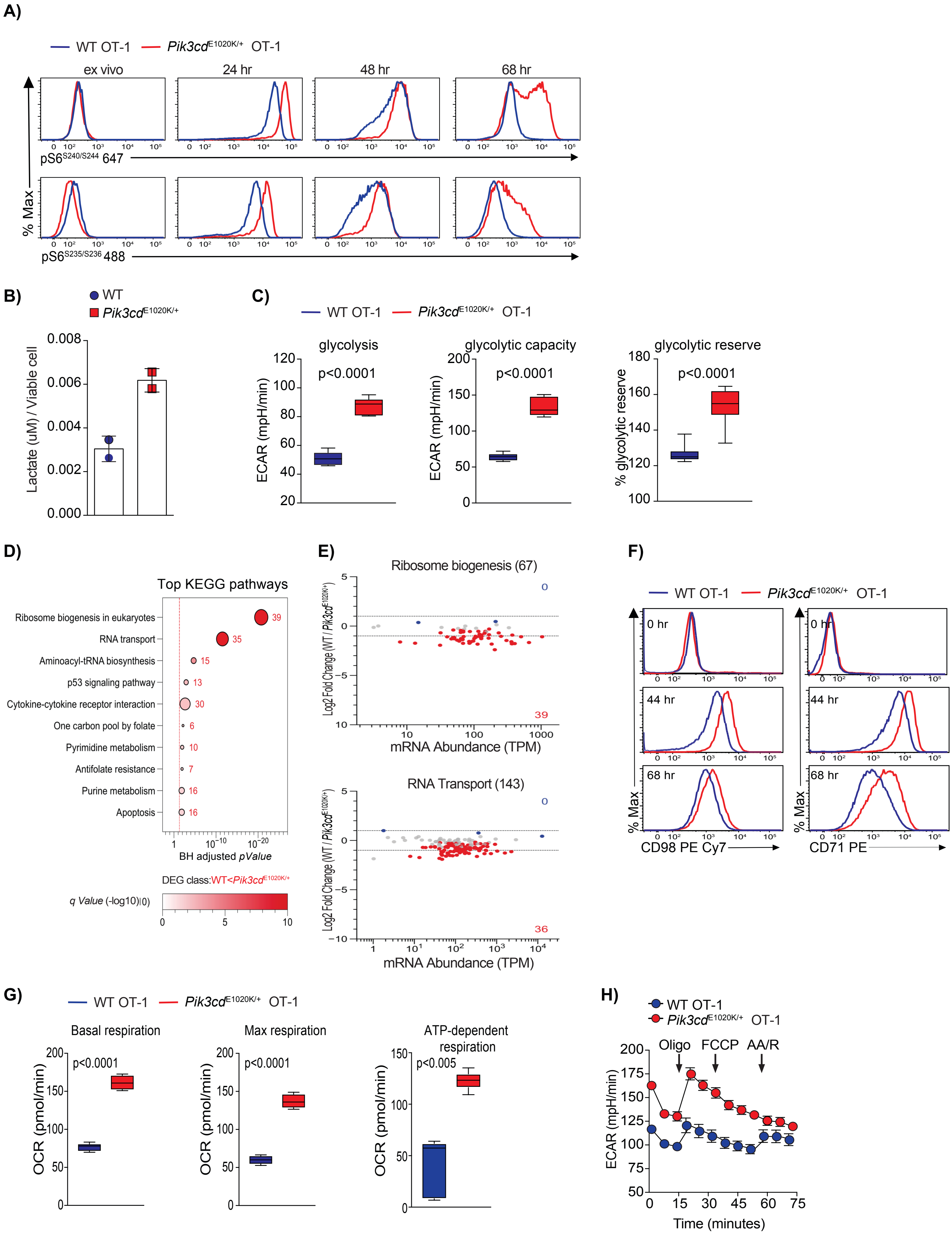

Figure S5. Activated *Pik3cd*$^{E1020K/+}$ OT-1 T cells demonstrate elevated and sustained pS6 and c-Myc expression accompanied by metabolic perturbations.

Related to Figure 4.

(A) Flow cytometric analysis of pS6$^{S235/236}$ and pS6$^{S240/244}$ from viable WT and *Pik3cd*$^{E1020K/+}$ OT-1 cells stimulated with peptide for the indicated times (n=3, representative histogram shown). (B) L-lactate was measured in CD8^{+} T cell supernatants by ELISA, 24 hr following anti-CD3 plus anti-CD28 stimulation (n=2). (C) WT and *Pik3cd*$^{E1020K/+}$ OT-1 CD8^{+} T cells were stimulated with peptide for 3 days. Sorted viable CD8^{+} T cells were evaluated via extracellular flux: ECAR was measured under basal conditions and in response to exogenous glucose, oligomycin and 2-deoxy-D-glucose. Glycolysis, glycolytic capacity and reserve are shown (n=3, representative example shown). (D-E) RNA-Seq analysis of gene expression. D) Scatter plot shows HGT data for top 10 enriched KEGG pathways among positively regulated DEGs (*Pik3cd*$^{E1020K/+}$>WT). Size and color saturations are proportional to gene count (total shown) and *p* value, respectively, n=3 biological replicates. (E) M (log ratio) and A (mean average) data (MA plots) show TPM and log2 fold change for all genes within selected KEGG pathways. Negatively (*Pik3cd*$^{E1020K/+}$<WT) and positively (*Pik3cd*$^{E1020K/+}$>WT) regulated DEGs are highlighted in blue and red, respectively. (F) Flow cytometric analysis of peptide stimulated OT-1 cells: CD98 (left panel) and CD71 (right panel), (n=3, representative example). (G-H) OT-1 T cells stimulated with peptide for 3 days. Extracellular flux analysis on sorted viable CD8^{+} T cells: G) OCR, basal respiration, maximal respiration and ATP-dependent respiration calculated as in methods and H) ECAR in response to oligomycin, FCCP, and antimycin A plus rotenone in the presence of glucose (n=3, representative example). Graph B show mean ± SEM. Graphs C, G and H show mean ± SEM *p value in graph.

Supplemental Figure 6

A)

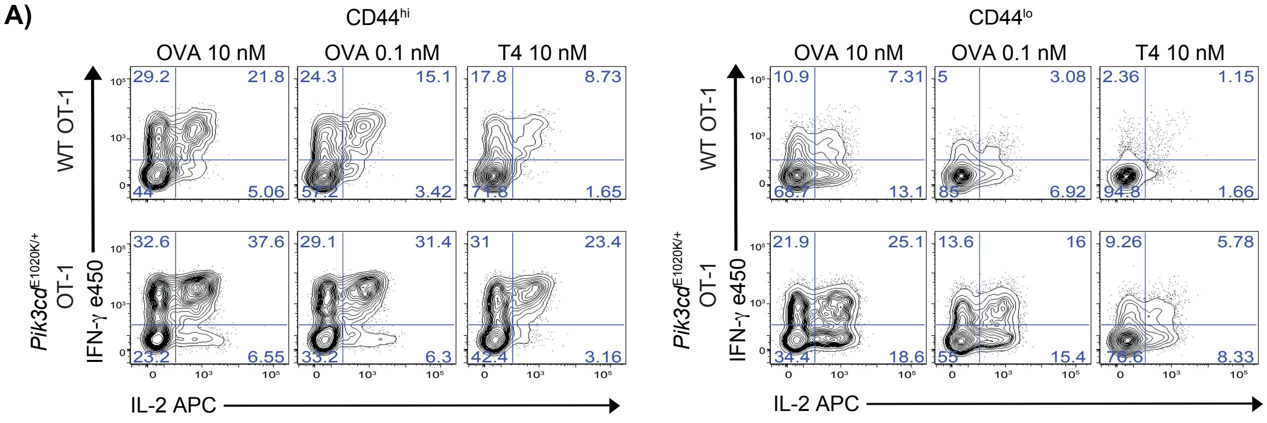

Figure S6. ***Pik3cd*$^{E1020K/+}$ OT-1 CD8^{+} T cells produce early and elevated IL-2.**

Related to Figure 5.

(A) OT-1 T cells were stimulated with peptide and Golgi Stop for 3hr. Viable CD8^{+} T cells gated on CD44^{hi} (left panels) or CD44^{lo} (right panels) expression were assessed for IFN-γ and IL-2 production. Representative flow plots shown, (n=3).

Supplemental Figure 7

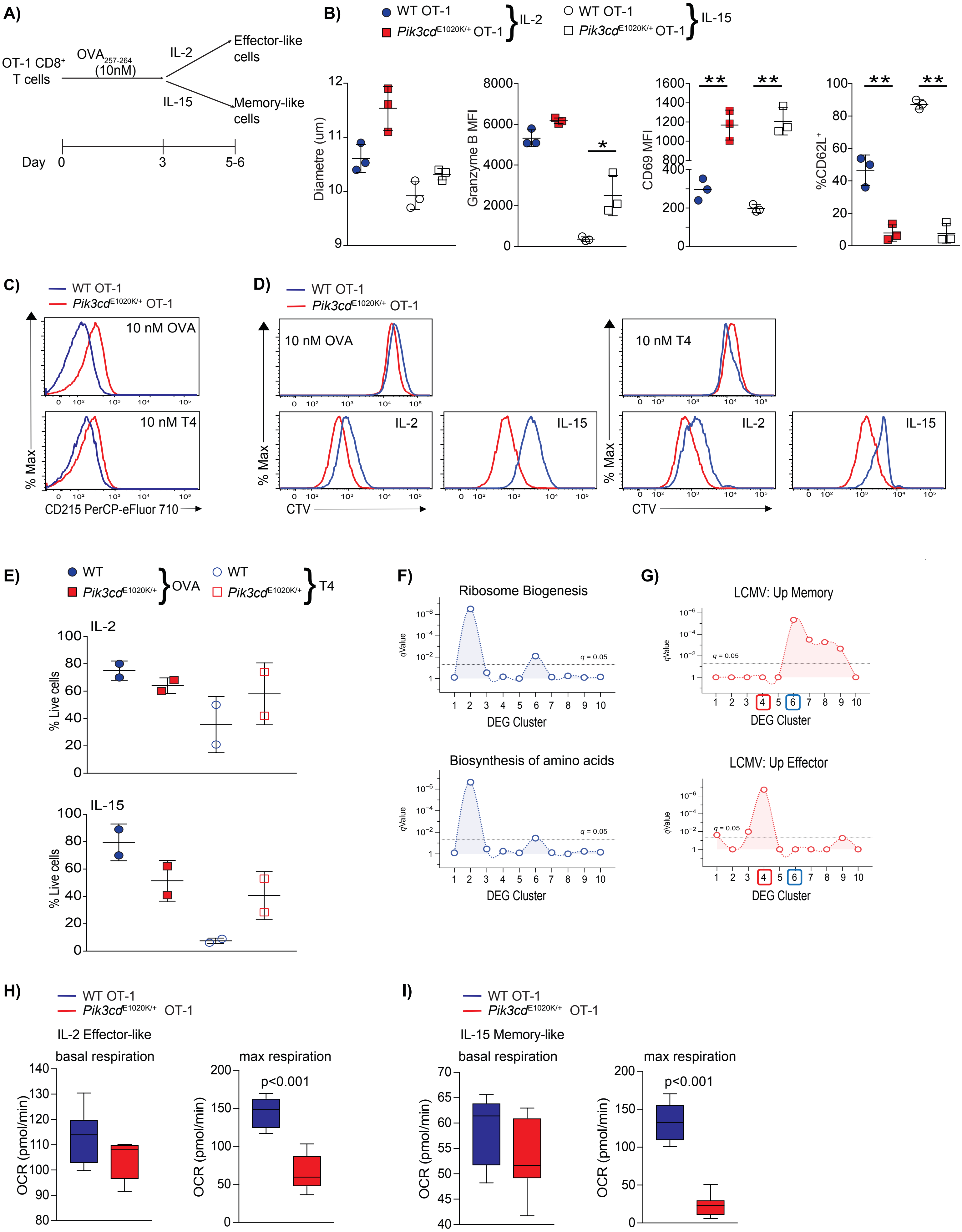

Figure S7. IL-15 differentiated *Pik3cd*$^{E1020K/+}$ OT-1 CD8^{+} T cells display features exemplified by lytic effector cells.

Related to Figure 6.

(A-B) OT-1 cells were stimulated with peptide for 3 days and subsequently cultured with cytokine to generate IL-2-effectors or IL-15-memory cells. A) Experimental outline. B) Cell diameter and flow cytometric analysis evaluating Granzyme B, CD69 and CD62L expression (n=3, summary plots shown). (C) OT-1 cells were activated with peptide for 3 days and assessed for CD215 expression, (n=3, representative histogram shown). (D-E) OT-1 cells were activated with $OVA_{257-264}$ or T4 for 3 days. Viable cells were CTV labeled and cultured in either IL-2 or IL-15 for 2 days. D) Cell expansion evaluated by CTV dilution. E) Cell viability evaluated post-cytokine culture. (F-G) RNA-Seq analysis of gene expression at day 6 on sorted viable OT-1 cells. Line graphs show enrichment of Kyoto Encyclopedia of Genes and Genomes (KEGG) pathways (F) or curated effector/memory CD8^{+} T cell gene sets (G) across 10 row clusters from (Figure 6C) https://www.gsea-msigdb.org/gsea/msigdb/cards/KAECH_DAY15_EFF_VS_MEMORY_CD8_TCELL_DN.html. (H-I) OT-1 cells were peptide stimulated for 3 days and subsequently cultured with either H) IL-2 or I) IL-15 to generate effector-like and memory-like cells, respectively. Extracellular flux analysis: OCR measured in real time under basal conditions and in response to mitochondrial inhibitors oligomycin, FCCP and antimycin A plus rotenone. OCR: basal and maximal respiration assessed (representative data shown, n=4). Graphs B and E show mean ± SEM *p<0.05, **p<0.01.Graphs H and I show mean ± SEM *p value in graph.

Supplemental Figure 8

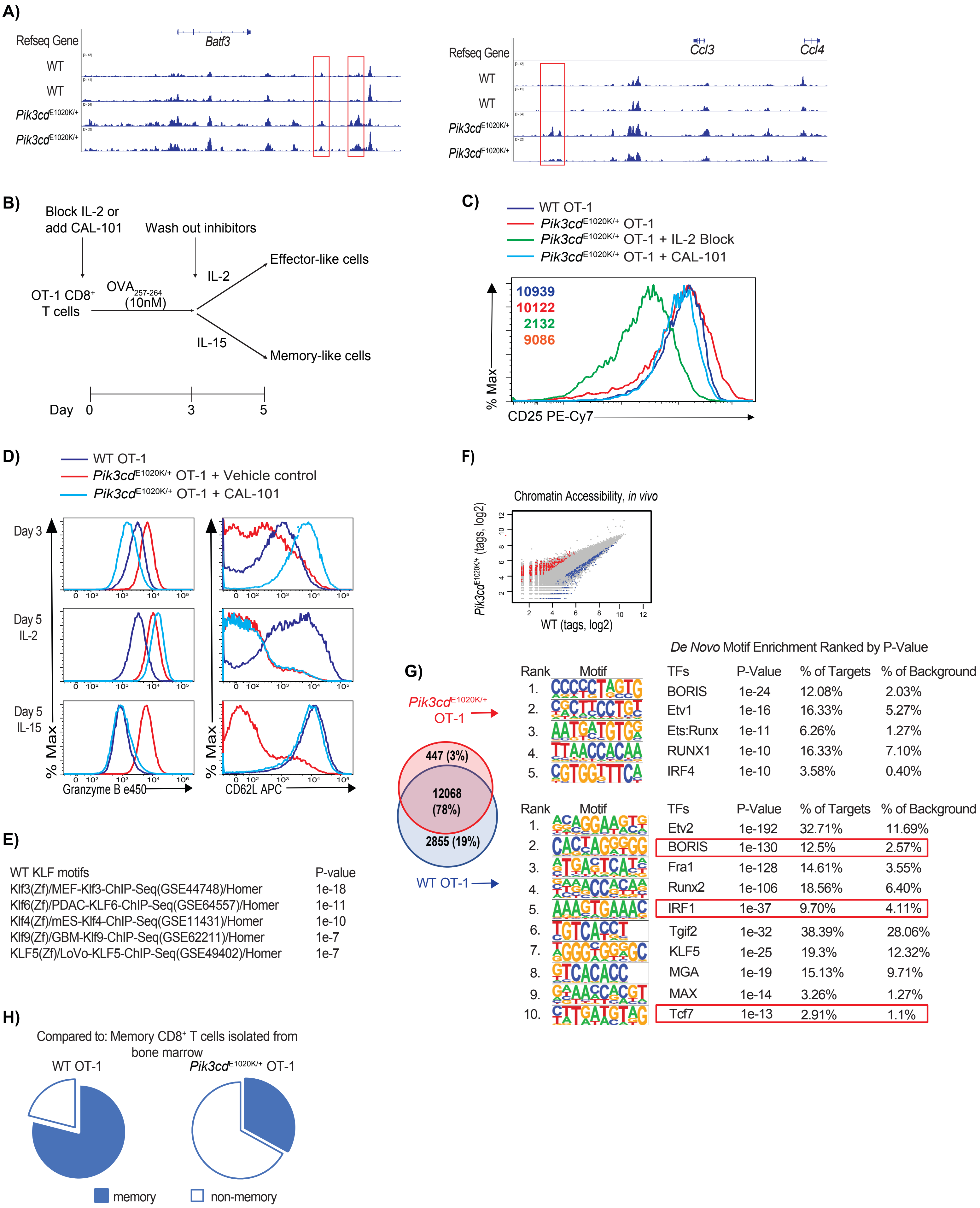

Figure S8. **Effector differentiation of *Pik3cd*$^{E1020K/+}$ CD8^{+} T cells is determined by IL-2. Related to Figure 7.**

(A) Genomic tracks of ATAC-Seq signal profile across *Batf3* (left panel) and *Ccl3* (right panel). Red boxes highlight differences in chromatin accessibility. (B-D) OT-1 cells were stimulated with peptide for 3 days +/- blocking IL-2 antibodies, p110δ inhibitor CAL-101, Ig control or vehicle control and subsequently cultured with either IL-2 or IL-15 to generate effector-like or memory-like cells, respectively (n=3). B) Experimental outline. C) Flow cytometry analysis of CD25 expression on day 3, representative histogram shown. D) Flow cytometric analysis of Granzyme B and CD62L evaluated +/- CAL-101, representative example shown (n=3). (E) ATAC-Seq analysis of day 3 activated sorted viable WT and *Pik3cd*$^{E1020K/+}$ OT-1 cells (n=2 independent experiments). *De novo* motif discovery using revealed KLF motifs enriched in WT cells. (F-G) ATAC-Seq analysis of sorted viable lung WT and *Pik3cd*$^{E1020K/+}$ OT-1 cells day 7 following influenza X31-OVA infection (n=2). E) Scatter plots of normalized ATAQ-Seq tag density compare chromatin accessibility (fold change>2; FDR<0.05). F) *De novo* motif discovery using HOMER revealed enrichment of BORIS, IRF4 and Tcf7 enriched motifs in WT cells (lower half). (H) Pie chart illustrating overlap of unique ATAC-Seq peaks from WT and *Pik3cd*$^{E1020K/+}$ day 3 activated CD8^{+} T cells with peaks from memory CD8^{+} T cells isolated from bone marrow.
