# Supplementary material for: PI3Kδ coordinates transcriptional, chromatin, and metabolic changes to promote effector CD8+ T cells at the expense of central memory
Source: Cell Rep. Author manuscript; Available in PMC 2021 Nov 11. (PMC8582080; doi:10.1016/j.celrep.2021.109804)
Supplement: 4 [file NIHMS1747909-supplement-4.pdf]

Cell Reports

Article

PI3Kδ coordinates transcriptional, chromatin, and metabolic changes to promote effector CD8$^+$ T cells at the expense of central memory

Graphical abstract

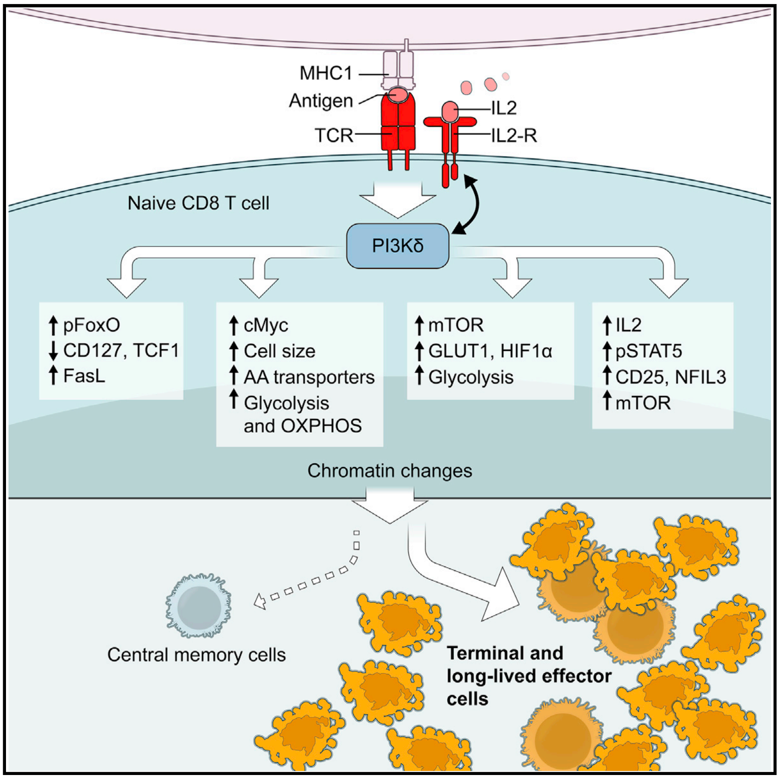

Highlights

- Activated PI3Kδ drives accelerated effector T cell function and FasL-driven apoptosis
- Activated PI3Kδ increases IL-2, mTOR, and Myc signatures and alters metabolism
- Activated PI3Kδ is associated with decreased TCF1 and altered chromatin
- After infection, PI3Kδ drives terminal and long-lived effector cells but poor T_{CM}

Authors

Jennifer L. Cannons,
Alejandro V. Villarino,
Senta M. Kapnick, ..., Peter J. McGuire,
Gulbu Uzel, Pamela L. Schwartzberg

Correspondence

jcannons@mail.nih.gov (J.L.C.),
pams@nih.gov (P.L.S.)

In brief

Using T cells from patients and a mouse model of activated PI3Kδ syndrome (APDS), Cannons et al. provide evidence that activated PI3Kδ drives transcriptional, chromatin, and metabolic changes involving IL-2, mTOR, Myc, and TCF1 that promote the differentiation of terminal and long-lived effector populations at the expense of central memory cells.

Cannons et al., 2021, Cell Reports *37*, 109804
October 12, 2021 © 2021 The Author(s).
https://doi.org/10.1016/j.celrep.2021.109804

Cell Reports

CellPress
OPEN ACCESS

Article

PI3Kδ coordinates transcriptional, chromatin, and metabolic changes to promote effector CD8+ T cells at the expense of central memory

Jennifer L. Cannons,[1,2,*] Alejandro V. Villarino,[3,6,11] Senta M. Kapnick,[2,7,11] Silvia Preite,[1,2,11] Han-Yu Shih,[3,4,11] Julio Gomez-Rodriguez,[1,2,8] Zenia Kaul,[1] Hirofumi Shibata,[1] Julie M. Reilley,[1,2] Bonnie Huang,[1,2] Robin Handon,[2] Ian T. McBain,[1] Selamawit Gossa,[5] Tuoqi Wu,[1,2,9,10] Helen C. Su,[1] Dorian B. McGavern,[5] John J. O'Shea,[3] Peter J. McGuire,[2] Gulbu Uzel,[1] and Pamela L. Schwartzberg[1,2,12,*]

[1]National Institute of Allergy and Infectious Diseases, NIH, Bethesda, MD 20892, USA
[2]National Human Genome Research Institute, NIH, Bethesda, MD 20892, USA
[3]National Institute of Arthritis and Musculoskeletal and Skin Diseases, NIH, Bethesda, MD 20892, USA
[4]National Eye Institute, NIH, Bethesda, MD 20892, USA
[5]National Institute of Neurological Disorders and Stroke, NIH, Bethesda, MD 20892, USA
[6]Department of Microbiology & Immunology and Sylvester Comprehensive Cancer Center, University of Miami, Miami, FL 33136, USA
[7]Fischell Department of Bioengineering, University of Maryland, College Park, MD 20742, USA
[8]TCR2 Therapeutics, Cambridge, MA 02142, USA
[9]University of Colorado, Department of Immunology, Denver, CO 80204, USA
[10]Department of Immunology and Harold C. Simmons Comprehensive Cancer Center, UT Southwestern Medical Center, Dallas, TX 75390
[11]These authors contributed equally
[12]Lead contact
*Correspondence: jcannons@mail.nih.gov (J.L.C.), pams@nih.gov (P.L.S.)
https://doi.org/10.1016/j.celrep.2021.109804

SUMMARY

Patients with activated phosphatidylinositol 3-kinase delta (PI3Kδ) syndrome (APDS) present with sinopulmonary infections, lymphadenopathy, and cytomegalvirus (CMV) and/or Epstein-Barr virus (EBV) viremia, yet why patients fail to clear certain chronic viral infections remains incompletely understood. Using patient samples and a mouse model (*Pik3cd*$^{E1020K/+}$ mice), we demonstrate that, upon activation, *Pik3cd*$^{E1020K/+}$ CD8$^+$ T cells exhibit exaggerated features of effector populations both *in vitro* and after viral infection that are associated with increased Fas-mediated apoptosis due to sustained FoxO1 phosphorylation and *Fasl* derepression, enhanced mTORC1 and c-Myc signatures, metabolic perturbations, and an altered chromatin landscape. Conversely, *Pik3cd*$^{E1020K/+}$ CD8$^+$ cells fail to sustain expression of proteins critical for central memory, including TCF1. Strikingly, activated *Pik3cd*$^{E1020K/+}$ CD8$^+$ cells exhibit altered transcriptional and epigenetic circuits characterized by pronounced interleukin-2 (IL-2)/STAT5 signatures and heightened IL-2 responses that prevent differentiation to memory-like cells in IL-15. Our data position PI3Kδ as integrating multiple signaling nodes that promote CD8$^+$ T cell effector differentiation, providing insight into phenotypes of patients with APDS.

INTRODUCTION

Following T cell receptor (TCR) ligation and cytokine signals, naive CD8$^+$ T cells proliferate and differentiate into effector cytotoxic T lymphocytes (CTLs) that express cytolytic proteins and help eliminate virus-infected cells and tumors (Stinchcombe and Griffiths, 2007). When infections are resolved, the CTL population contracts; however, a fraction persists as long-lived memory cells, including central and effector memory populations (T_{CM} and T_{EM} cells) that provide protection against subsequent infection (Hashimoto et al., 2019). T_{CM} cells, in particular, expand upon rechallenge and maintain long-term immunity. Recently, it has been recognized that T_{EM} contain a subset of long-lived effector cells (LLECs) or terminally differentiated (terminal)-T_{EM} cells with potent cytolytic activity, in addition to longer-lived true T_{EM} cells that express high CD127 and CD27 (Milner et al., 2020; Renkema et al., 2020).

Many studies have enumerated factors critical for T cell memory, including cytokines, transcription factors (TFs), chromatin remodelers, and metabolic regulators. Of note, there is considerable overlap between the transcriptomes of CD8$^+$ T_{CM} cells and TCF1$^+$ stem-like progenitor cells that maintain long-term T cell responses in chronic infection and cancer (McLane et al., 2019). Thus, elucidating mechanisms guiding T_{CM} cell generation is important both for understanding memory in acute infection and harnessing immune responses in chronic infection. Whether there is a common theme unifying the regulation of effector/memory cell fate decisions remains unclear.

Class IA phosphoinositide 3-kinases (PI3Ks) are lipid kinases that are important for lymphocyte signaling, differentiation,

© 2021 The Author(s).
This is an open access article under the CC BY license (http://creativecommons.org/licenses/by/4.0/).

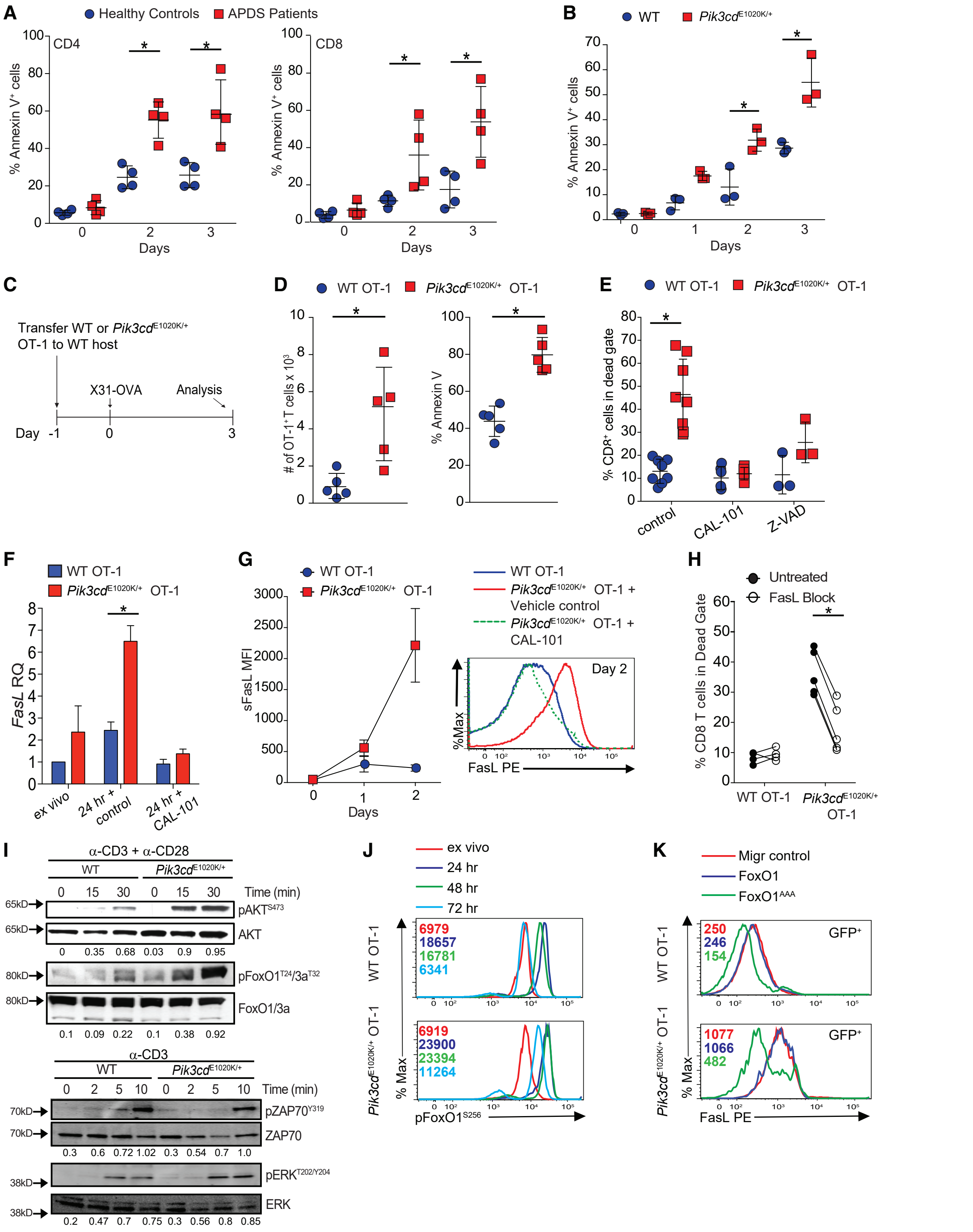

Figure 1. Activated PI3Kδ T cells show increased cell death, FasL, and pFoxO1

(A) Annexin-V$^+$ T cells from healthy controls and patients with APDS stimulated with anti-CD3 plus anti-CD28 (n = 4).

(B) Annexin-V staining of sorted naive (CD62L^{hi}CD44^{lo}) CD8$^+$ cells stimulated with anti-CD3 plus anti-CD28 (n = 3).

(C and D) OT-1 cells were transferred into CD45 congenic hosts subsequently infected with X31-OVA influenza. (C) Experimental outline. (D) Left: live OT-1 cell numbers. Right: Annexin-V$^+$ cells, (n = 2, 2–3 mice/group).

(legend continued on next page)

CellPress
OPEN ACCESS

survival, metabolism, and migration (Okkenhaug, 2013); p110δ is the predominant catalytic isoform in leukocytes. Upon activation by cell surface receptors, p110 generates phosphatidylinositol-3,4,5-triphosphate (PIP_3), which recruits proteins containing pleckstrin homology and other PIP_3-binding domains to the membrane (Okkenhaug, 2013). These include AKT kinases, which phosphorylate FoxO and other TFs, regulators of the mammalian target of rapamycin (mTOR) complex I, and other targets (Okkenhaug, 2013). Several of these molecules are downstream effectors of the cytokines interleukin-2 (IL-2) and IL-15, which influence T cell activation and memory, respectively.

Patients with gain-of-function mutations in *PIK3CD*, which encodes p110δ, exhibit a primary immunodeficiency, activated PI3Kδ syndrome (APDS), characterized by lymphopenia, lymphoproliferation, and recurrent respiratory infections (Angulo et al., 2013; Coulter et al., 2017; Lucas et al., 2014). A significant fraction of patients have persistent Epstein-Barr virus (EBV) or cytomegalovirus (CMV) viremia, suggesting defective clearance of certain chronic viral infections, despite having EBV-specific $CD8^+$ T cells (Lucas et al., 2014). How activated PI3Kδ links to specific phenotypes remains only partially understood.

Here, we use a mouse model of patients with APDS (*Pik3cd*$^{E1020K/+}$ mice) (Preite et al., 2018) and patient samples to dissect how hyperactive PI3Kδ influences $CD8^+$ T cell survival, differentiation, and function. We find that a substantial fraction of activated PI3Kδ T cells die via TCR-induced apoptosis (Angulo et al., 2013; Edwards et al., 2019), which we link to impaired FoxO1-mediated repression of *Fasl*. Surviving *Pik3cd*$^{E1020K/+}$ $CD8^+$ T cells display enhanced and sustained mTOR and c-Myc activation, metabolic reprogramming, and increased expression of protein synthesis machinery consistent with an accelerated effector program. In contrast, *Pik3cd*$^{E1020K/+}$ cells fail to maintain expression of TCF1 and show a shift to an LLEC phenotype after infection. Cellular, transcriptome, and ATAC-seq analyses of *Pik3cd*$^{E1020K/+}$ $CD8^+$ T cells reveal early and amplified IL-2 responses that propel cells toward an effector fate, with a loss of memory potential manifested at the level of chromatin. Our work positions PI3Kδ as a master regulator of signaling and transcription networks that determine effector/memory lineage decisions and argues that stringent regulation of PI3Kδ is required for appropriate development of memory.

RESULTS

T cells from patients with APDS and *Pik3cd*$^{E1020K/+}$ mice die prematurely via FasL-mediated apoptosis

To understand how activated PI3Kδ affects $CD8^+$ T cell differentiation and function, we stimulated peripheral blood mononuclear cells (PBMCs) from healthy controls and patients with APDS with anti-CD3 plus anti-CD28. Strikingly, $CD4^+$ and $CD8^+$ T cells from patients with APDS exhibited high percentages of Annexin-V^+ cells (Figure 1A; Angulo et al., 2013; Bier et al., 2019). Similar results were observed with activated T cells from *Pik3cd*$^{E1020K/+}$ mice or *Pik3cd*$^{E1020K/+}$ OT-I mice expressing a class I major histocompatibility complex (MHC)-restricted transgenic TCR specific for the ovalbumin (OVA) peptide $OVA_{257-264}$, although surviving cells also showed evidence of increased proliferation (Figures S1A–S1C). Although patients with APDS and *Pik3cd*$^{E1020K/+}$ mice exhibit reduced naive T cells (Figure S1D; Angulo et al., 2013; Avery et al., 2018; Lucas et al., 2014; Preite et al., 2018; Stark et al., 2018), enhanced cell death was also observed in sorted naive ($CD62L^+CD44^{lo}$) *Pik3cd*$^{E1020K/+}$ $CD8^+$ T cells post-stimulation (Figure 1B). To determine whether increased death was cell intrinsic, we evaluated cells from mixed bone marrow (BM) chimeras (outline Figure S1E). Wild-type (WT) hosts receiving BM from either WT or *Pik3cd*$^{E1020K/+}$ donors reproduced the cellular phenotype of intact donor mice (Figure S1F, open symbols). In contrast, in mixed BM chimeras, the presence of *Pik3cd*$^{E1020K/+}$ cells converted WT T cells to an activated phenotype, with even higher percentages of $CD44^{hi}$ cells than *Pik3cd*$^{E1020K/+}$ cells (Figure S1F, upper panel, closed symbols) (Bier et al., 2019; Jia et al., 2021). Nonetheless, when T cells from mixed chimeras were stimulated *in vitro*, more *Pik3cd*$^{E1020K/+}$ T cells died than WT cells in the same cultures (Figure S1F, lower panel, closed symbols). Thus, increased cell death of *Pik3cd*$^{E1020K/+}$ T cells was cell intrinsic and not solely the result of prior activation state.

To evaluate antigen-induced cell death *in vivo*, WT and *Pik3cd*$^{E1020K/+}$ OT-1 cells were adoptively transferred into congenic hosts that were then infected with influenza X31 expressing the $OVA_{257-264}$ peptide (outline Figure 1C). Three days post-infection (p.i.), an elevated number of *Pik3cd*$^{E1020K/+}$ OT-1 cells were found in the lung compared to WT counterparts (Figure 1D, left panel). However, a higher percentage of *Pik3cd*$^{E1020K/+}$ OT-1 cells were Annexin-V^+ (Figure 1D, right panel).

Inclusion of CAL-101, a selective p110δ inhibitor, prevented increased cell death in cultured *Pik3cd*$^{E1020K/+}$ OT-1 cells (Figure 1E), confirming this phenotype was linked to PI3Kδ activity. Cell death was primarily apoptotic, as it was associated with increased active caspase-3 and cleaved PARP1 (Figures S1G and S1H) and partially blocked by Z-VAD, a pan-caspase inhibitor (Figure 1E), but not by Necrostatin-1, an inhibitor of necroptosis (Figure S1I). A prominent mechanism of apoptosis in activated T cells is mediated by Fas-FasL signals following TCR restimulation. Upon peptide stimulation, *Pik3cd*$^{E1020K/+}$

(E–G) OT-1 cells stimulated with $OVA_{257-264}$ with or without CAL-101 or Z-VAD (n = 3–5). (E) Cell death. (F) *Fasl* mRNA. (G) Surface FasL Mean fluorescence intensity (MFI) (left) and a representative histogram at 48 h (right).

(H) Viability of OT-1 cells stimulation with or without blocking FasL, 48 h (n = 4).

(I) Top: T cells stimulated with anti-CD3 plus anti-CD28. Immunoblot for $pAKT^{S473}$, AKT, $pFoxO1^{T24}/FoxO3a^{T32}$, and FoxO1. Bottom: T cells stimulated with anti-CD3. Immunoblot for $pZAP70^{Y319/Y352}$, ZAP70, $pERK^{T202/Y204}$, and ERK (representative blots, n = 3).

(J) $pFoxO1^{S256}$ of OT-1 cells stimulated with $OVA_{257-264}$ (n = 3, representative histogram, with MFI indicated).

(K) Surface FasL on viable GFP^+ OT-1 cells retrovirally transduced with Migr (control), Migr-FoxO1, or Migr-$FoxO1^{AAA}$ (n = 3, representative histogram).

Graphs show mean ± SEM. *p < 0.05. See Figure S1.

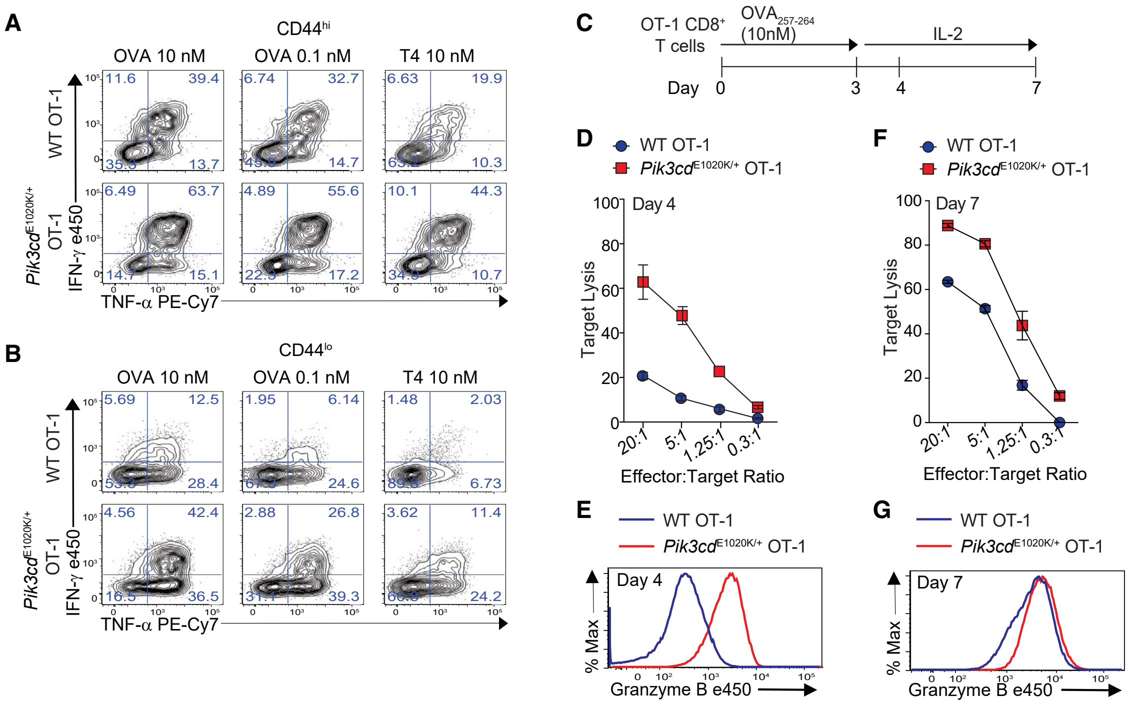

Figure 2. Early and enhanced effector phenotype of *Pik3cd*$^{E1020K/+}$ OT-1 CD8^{+} T cells

(A and B) IFN-γ and TNF-α production from OT-1 cells stimulated with indicated peptide for 3 h. Shown are CD44^{hi} (A) CD44^{lo} (B) cells (n = 3, representative flow plots).

(C–G) OT-1 cells were stimulated with OVA$_{257–264}$ for 3 days and then expanded in IL-2. (C) Outline. (D and F) *In vitro* cytolysis of LPS-activated B cells pulsed with 1 nM OVA$_{257–264}$ by day 4 (D) or day 7 (F) CTLs (n = 3). (E and G) GzmB on day 4 (E) and day 7 (G) (n = 3, representative histogram).

Graphs show mean ± SEM. See Figure S2.

OT-1 cells rapidly upregulated *Fasl* mRNA and surface FasL to a far greater extent than WT cells (Figures 1F and 1G); similar findings were observed with T cells from patients with APDS (Figure S1J). PI3Kδ inhibition prevented increased *Fasl* mRNA and surface FasL induction (Figures 1F and 1G), and blocking Fas/FasL interaction improved viability (Figure 1H). The data suggest that FasL-Fas-driven apoptosis is a major driver of increased cell death in activated PI3Kδ T cells.

FoxO1 represses *Fasl* expression in T cells

FoxOs are major PI3K-regulated TFs that are excluded from the nucleus and inactivated by AKT-mediated phosphorylation. Intriguingly, in neutrophils, FoxO3 represses *Fasl* expression (Jonsson et al., 2005). TCR-stimulated *Pik3cd*$^{E1020K/+}$ T cells exhibited elevated and sustained pAKT^{S473} and pFoxO1/3 (Figure 1I, top panels, and Figure 1J; Angulo et al., 2013; Lucas et al., 2014; Preite et al., 2018; Stark et al., 2018), whereas early TCR-mediated signaling, including pZAP70 and pERK, was minimally affected (Figure 1I, bottom panels). Moreover, inhibition of either p110δ or AKT decreased FasL expression and cell death (Figures S1K and S1L). Expression of FoxO1^{AAA}, a mutant that cannot be phosphorylated by AKT and is resistant to inactivation by PI3Kδ (Zhang et al., 2002), reduced FasL expression in both in WT and *Pik3cd*$^{E1020K/+}$ cells (Figure 1K). Thus, FoxO1 functions as a transcriptional repressor of *Fasl* in T cells.

Pik3cd$^{E1020K/+}$ OT-1 exhibit accelerated and enhanced effector function

When naive CD8^{+} T cells are activated, they undergo clonal expansion and differentiation into effectors that express cytotoxic molecules such as granzyme B (GzmB) and FasL and anti-viral cytokines (Hashimoto et al., 2019). Like FasL, *Pik3cd*$^{E1020K/+}$ OT-1 cells stimulated *ex vivo* produced excessive interferon-γ (IFN-γ) and tumor necrosis factor α (TNF-α) compared to WT OT-1 cells (Figure 2A). This increase was observed with low or high peptide, a weakly stimulating peptide (T4), and even in CD44^{lo} cells, suggesting a loss of quiescence and/or increased capacity to be rapidly activated even under suboptimal conditions (Figures 2A and 2B).

To evaluate CTL function, OT-1 cells were peptide stimulated, cultured with IL-2 to permit acquisition of cytolytic function, and then exposed to target cells (Figure 2C, outline). Surprisingly, surviving *Pik3cd*$^{E1020K/+}$ OT-1 cells displayed robust GzmB expression and killed targets efficiently after only 1 day in IL-2, when WT cells had not fully acquired cytolytic effector function (Figures 2D and 2E). By day 7, WT CTLs had strong cytolytic capacity, but *Pik3cd*$^{E1020K/+}$ OT-1 cells still expressed slightly more GzmB and killed targets better (Figures 2F and 2G). *Pik3cd*$^{E1020K/+}$ OT-1 effectors also exhibited elevated LFA-1 and adhesion to B cell targets (Figures S2A and S2B) as well as increased CD244, a costimulatory receptor that fine-tunes CTL function and killing of EBV-infected targets (Cannons

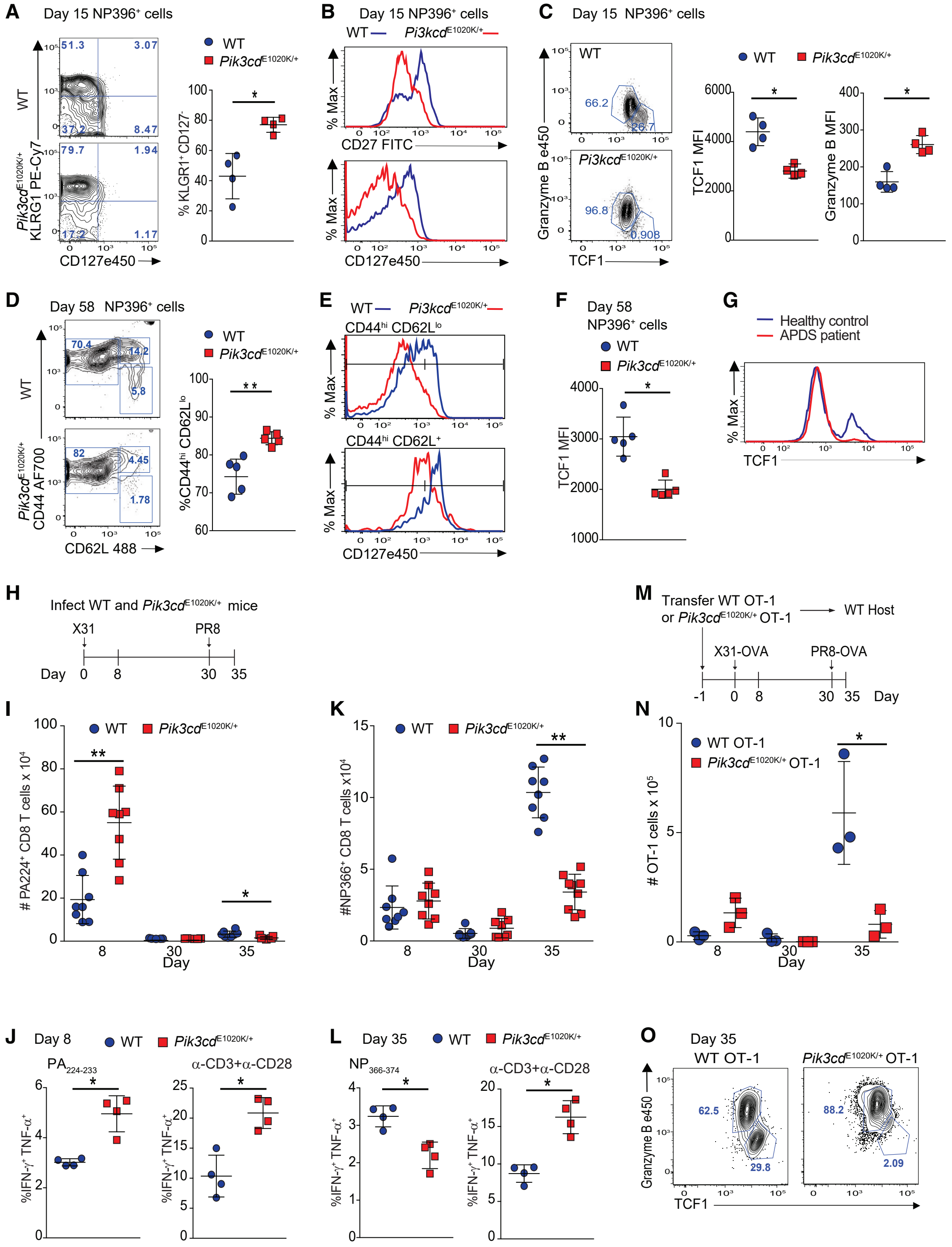

Figure 3. *Pik3cd*$^{E1020K/+}$ mice fail to develop a robust T_{CM} population

(A–F) Viable CD8$^+$ splenocytes from mice infected with LCMV Armstrong (n = 2, 4–5/group/time point). (A–C) NP396-specific CD8$^+$ cells day 15 p.i. (A) CD127 and KLRG1 expression (left: representative staining; right: %KLRG1$^+$CD127$^-$). (B) Representative histogram of CD27 (top) and CD127 (bottom). (C) GzmB and TCF1 staining. Middle: TCF1 MFI; right: GzmB MFI. (D–F) NP396-specific CD8$^+$ cells day 58 p.i. (D) CD44 and CD62L staining. Representative flow (left), % CD44^{hi}CD62L^{lo} cells (right). (E) CD127 histograms: CD44^{hi}CD62L^{lo} (top) and CD44^{hi}CD62L$^+$ (bottom). (F) TCF1 MFI.

(G) TCF1 staining of allo-reactive CD8$^+$ cells from healthy controls and patients with APDS (n = 2, representative histogram).

(legend continued on next page)

et al., 2018; Zhao et al., 2012; Figure S2A). Thus, hyperactive PI3Kδ drives accelerated and pronounced cytolytic effector function.

Activated PI3Kδ drives terminal effector T cell differentiation following viral infection

To evaluate effector phenotypes *in vivo*, mice were infected with lymphocytic choriomeningitis virus (LCMV) Armstrong. Evaluation at day 4 revealed lower viral loads in *Pik3cd*$^{E1020K/+}$ mice compared to WT, consistent with their increased cytolytic capacity (Figures S3A and 2D). By day 8, both genotypes cleared infection from the liver and serum (Matloubian et al., 1994; Figure 3SA; data not shown) and had comparable numbers and frequencies of NP396-specific CD8$^+$ T cells (Figures S3B and S3C) with similar phenotypes, although there were slightly higher percentages of KLRG1$^+$CD127$^-$ NP396-specific effector cells in the *Pik3cd*$^{E1020K/+}$ mice (Figures S3D and S3E). However, by day 15, percentages and numbers of effector-like antigen-specific cells were clearly elevated in *Pik3cd*$^{E1020K/+}$ mice with increased KLRG1$^+$ cells that expressed low CD27 and CD127 (IL-7Rα), a marker of memory precursor cells that is regulated by the FoxO1 target KLF2 (Kerdiles et al., 2009; Kim et al., 2013; Figures 3A, 3B, and S3F–S3I).

TCF1 is a positively regulated FoxO1 target that is essential for efficient T_{CM} generation (Jeannet et al., 2010; Zhou et al., 2010). On day 8, both WT and *Pik3cd*$^{E1020K/+}$ antigen-specific T cells showed a similar bifurcation of GzmB^{hi} versus TCF1$^+$ cells. However, WT cells also displayed a small yet discernable TCF1^{hi}GzmB^{lo} population (Figure S3J, arrow). By day 15, most *Pik3cd*$^{E1020K/+}$ NP396-specific CD8$^+$ T cells maintained high GzmB expression yet failed to sustain a clear TCF1$^+$ population, unlike WT cells (Figure 3C). Moreover, although both WT and *Pik3cd*$^{E1020K/+}$ OT-1 cells initially upregulated TCF1 when activated *in vitro*, *Pik3cd*$^{E1020K/+}$ CTLs had reduced TCF1 levels after expansion in IL-2 (Figure S3K). Day 15 p.i. *Pik3cd*$^{E1020K/+}$ tetramer$^+$ CD8$^+$ T cells also had reduced Eomes, despite equivalent T-bet expression as WT (Figure S3L). Thus, *Pik3cd*$^{E1020K/+}$ CD8$^+$ T cells display a skewed differentiation toward effector cell phenotypes.

On day 58 post-LCMV infection, similar frequencies of antigen-specific T cells were observed in WT and *Pik3cd*$^{E1020K/+}$ splenocytes, yet *Pik3cd*$^{E1020K/+}$ mice had an increase in the absolute number of tetramer$^+$ CD8$^+$ T cells (Figures S3B and S3C), perhaps secondary to increased T cell numbers in lymphoid organs as the mice age (Preite et al., 2018). To evaluate memory cells, we assessed expression of the T_{CM} marker, CD62L as well as CD44 (Jeannet et al., 2010; Zhou et al., 2010). Antigen-specific CD8$^+$ T cells from *Pik3cd*$^{E1020K/+}$ mice displayed an elevated frequency and number of CD44^{hi}CD62L^{lo} effector-like memory with a reduction in the frequency of CD44^{hi}CD62L^{hi} T_{CM} cells that were CD127^{hi} compared to WT mice (Figures 3D, 3E, S3M, and S3N). Overall, *Pik3cd*$^{E1020K/+}$ antigen-specific cells expressed decreased CD27 and CD127 (Figures 3E and S3O). Thus, many of the CD44$^+$CD62L^{lo} cells exhibited features of recently described LLECs or terminal T_{EM} cells rather than true CD127$^+$ T_{EM} cells (Milner et al., 2020; Olson et al., 2013; Renkema et al., 2020). Antigen-specific *Pik3cd*$^{E1020K/+}$ cells also expressed less IL-2 (Figure S3P) and high KLRG1 (Figure S3Q), similar to LLECs. Furthermore, NP396-specific *Pik3cd*$^{E1020K/+}$ CD8$^+$ T cells expressed lower TCF1 than WT cells on day 58 (Figure 3F and S3R). Notably, alloreactive CD8$^+$ T cells cultured from patients with APDS also failed to maintain a TCF1^{hi} population compared to healthy control cells, providing evidence for parallel phenotypes in human cells (Figure 3G). Thus, *Pik3cd*$^{E1020K/+}$ CD8$^+$ T cells exhibit a pronounced effector-like phenotype with reduced expression of CD127 and TCF1, which are critical for the development of quiescent T_{CM} cells.

Activated PI3Kδ impairs memory T cell expansion following viral infection

To determine whether *Pik3cd*$^{E1020K/+}$ CD8$^+$ T cells develop functional memory, mice were infected with X31 (H3N2) influenza virus followed by challenge with PR8 (H1N1) influenza on day 30 (Figure 3H). These mouse-adapted strains do not elicit antibody-cross reactivity, allowing for study of CD8$^+$ T cell memory (Rutigliano et al., 2010; Sabbagh et al., 2006). *Pik3cd*$^{E1020K/+}$ mice cleared X31 more rapidly, with reduced viral titers compared to WT mice on day 4. By day 8, both genotypes had cleared the virus from the lung (Figure S4A), but *Pik3cd*$^{E1020K/+}$ mice showed increased accumulation of PA224-specific CD8$^+$ T cells compared to WT mice (Figure 3I). Percentages of *Pik3cd*$^{E1020K/+}$ CD8$^+$ T cells producing effector cytokines IFN-γ and TNF-α in response to $PA_{224–233}$ or anti-CD3 plus anti-CD28 were also elevated (Figure 3J).

NP366-specific cells are a subdominant primary response to X31 but the major population that expands upon PR8 challenge (Rutigliano et al., 2010; Sabbagh et al., 2006). Similar numbers of NP366-specific cells were generated in WT and *Pik3cd*$^{E1020K/+}$ mice upon X31 infection (Figure 3K). However, *Pik3cd*$^{E1020K/+}$ NP366-specific cells expanded poorly in response to PR8 challenge, and the magnitude of the secondary NP366-specific response in *Pik3cd*$^{E1020K/+}$ mice resembled their primary response (Figure 3K). Similarly, lower percentages of *Pik3cd*$^{E1020K/+}$ CD8$^+$ T cells from PR8-challenged mice produced effector cytokines than WT cells when stimulated with $NP_{366–374}$ peptide (Figure 3L, left panel). In contrast, CD8$^+$ T cells from PR8-challenged *Pik3cd*$^{E1020K/+}$ mice mounted a robust polyclonal response to anti-CD3 and CD28 stimulation, with elevated cytokine-producing cells, providing evidence of aberrant immune activation (Figure 3L, right panel). Furthermore, lung viral titers were comparable between *Pik3cd*$^{E1020K/+}$ and WT mice, suggesting that *Pik3cd*$^{E1020K/+}$ mice were able

(H–L) Mice were infected with X31 and challenged with PR8 (n = 2, 3–5 mice/genotype/time point). (H) Infection outline. (I) PA224-specific CD8$^+$ cell numbers. (J) IFN-γ and TNF-α from day 8 cells stimulated with either $PA_{224–233}$ (left) or anti-CD3 plus anti-CD28 (right). (K) NP366-specific CD8$^+$ T cell numbers. (L) IFN-γ and TNF-α from day 35 cells stimulated with either $NP_{366–374}$ (left) or anti-CD3 plus anti-CD28 (right).

(M–O) OT-1 cells were transferred into congenic hosts, infected with influenza X31-OVA and challenged with PR8-OVA (n = 2, 3 mice/genotype/time point). (M) Outline. (N) Viable OT-1 cell numbers. (O) TCF1 and GzmB expression in OT-1 cells on day 35. Representative experiment (n = 2).

Graphs show mean ± SEM. *p < 0.05; **p < 0.01. See Figures S3 and S4.

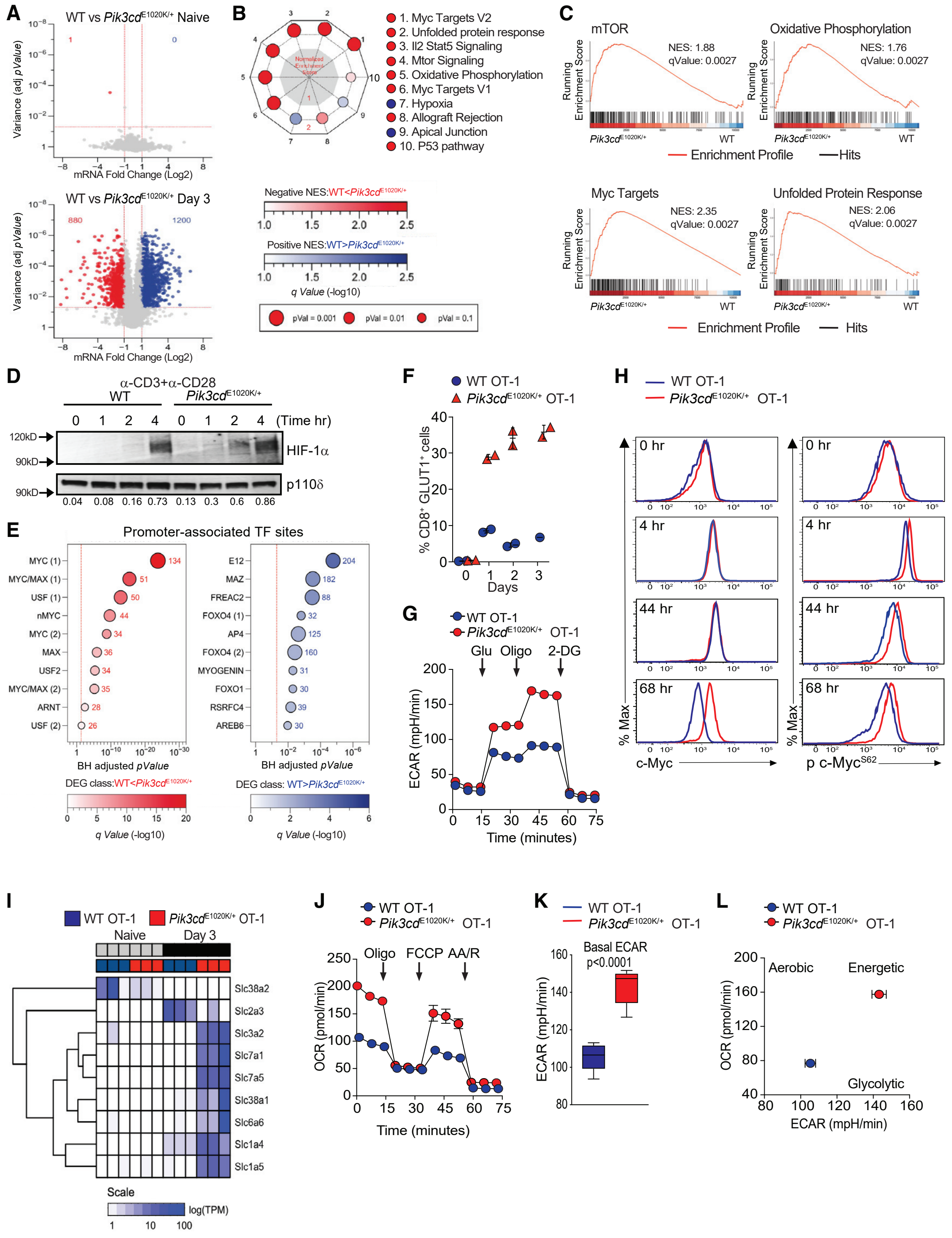

Figure 4. Enhanced mTORC1-pathways, sustained c-Myc, and metabolic perturbations in activated *Pik3cd*$^{E1020K/+}$ OT-I T cells

(A) Volcano plot shows log2 fold change and variance (Benjamini-Hochberg [BH] adjusted p value) for pairwise comparison of detectable transcripts from naive (top) and day 3 antigen-stimulated (bottom) OT-I cells. Negatively (*Pik3cd*$^{E1020K/+}$ < WT) and positively (*Pik3cd*$^{E1020K/+}$ > WT) DEGs are highlighted in blue and red, respectively.

(B) Radial plot of GSEA top 10 enriched MSigDB hallmark pathways. Distance from center denotes normalized enrichment score (NES). Color indicates directionality. Blue indicates enriched in WT, and red indicates enriched in *Pik3cd*$^{E1020K/+}$. Element size and saturation are proportional to p and *q* values, respectively.

(legend continued on next page)

to control viral load, consistent with functional LLECs (Figure S4A).

To determine whether aberrant T cell phenotypes in *Pik3cd*$^{E1020K/+}$ mice were cell intrinsic and not secondary to altered responses to a subdominant recall antigen or differences in viral milieu, we transferred WT and *Pik3cd*$^{E1020K/+}$ OT-1 cells into congenic hosts and infected mice with X31-OVA followed by PR8-OVA challenge (Figure 3M). *Pik3cd*$^{E1020K/+}$ OT-1 cells mounted robust primary responses with increased KLRG1^{hi} CD127^{lo} effector cells (Figure S4B) and elevated expression of Blimp-1, a critical TF for effector CD8^{+} T cell programing that is induced by IL-2 and repressed by BACH2, another TF that is inactivated by AKT and controls T cell quiescence, senescence, and survival (Ando et al., 2016; Figure S4C). However, *Pik3cd*$^{E1020K/+}$ OT-I cells displayed poor secondary expansion, suggestive of impaired memory cell proliferation and/or increased cell death (Figure 3N). Moreover, the TCF1^{hi} population was notably diminished in *Pik3cd*$^{E1020K/+}$ OT-1 donor cells, with most cells maintaining high GzmB (Figure 3O), confirming these phenotypes were T cell intrinsic.

Activated PI3Kδ drives aberrant mTORC1 activity associated with metabolic perturbations

To define molecular and genomic mechanisms by which hyperactive PI3Kδ disturbs CD8^{+} T cell differentiation, we utilized an *in vitro* culture system where the cellular environment could be finely controlled. Sorted naive (CD62L^{hi}CD44^{lo}) OT-1 cells were stimulated with antigen for 3 days and evaluated by RNA sequencing (RNA-seq). Transcriptomes for initial naive WT and *Pik3cd*$^{E1020K/+}$ OT-I cells were nearly indistinguishable (Figure 4A, upper panel). In contrast, after 3 days stimulation, 1,200 and 880 annotated transcripts were overrepresented in WT or *Pik3cd*$^{E1020K/+}$ cells, respectively (Figure 4A, lower panel). Gene set enrichment analysis (GSEA) of 50 "hallmark" functions and pathways (Subramanian et al., 2005) revealed the mTORC1 pathway was highly enriched in *Pik3cd*$^{E1020K/+}$ cells relative to WT (Figures 4B and 4C). mTORC1 is a conserved nutrient sensor that regulates protein synthesis and cell growth, CTL differentiation (Araki et al., 2009; Pollizzi et al., 2015; Sinclair et al., 2008), and induction of the HIF-1α TF, leading to increased glycolytic metabolism that accompanies T cell activation (Finlay et al., 2012; Nizet and Johnson, 2009). Accordingly, *Pik3cd*$^{E1020K/+}$ CD8^{+} T cells displayed enhanced and sustained induction of pS6$^{S240/244}$, a readout of mTORC1 activity (Figure S5A), and increased HIF-1α upon stimulation (Figure 4D).

Next we called differentially expressed genes (DEGs), segregated positively and negatively regulated fractions (mRNA increased or reduced in *Pik3cd*$^{E1020K/+}$ relative to WT), and performed hypergeometric testing (HGT) against gene sets cataloguing computationally defined DNA-binding motifs at gene promoters (Figure 4E; Liberzon et al., 2015). Binding motifs for ARNT (HIF-1β), an obligate partner of HIF-1α, were enriched within positively regulated DEG promoters in activated *Pik3cd*$^{E1020K/+}$ CD8^{+} T cells. In contrast, DEGs that were higher in WT cells included targets of FoxOs, as well as E12, a component of the E2a TF that is implicated in memory/effector cell decisions (Figure 4E; Masson et al., 2013; Yang et al., 2011).

Consistent with increased pS6 and HIF-1α, antigen-stimulated *Pik3cd*$^{E1020K/+}$ OT-1 cells displayed elevated GLUT1, a glucose transporter (Figure 4F) and increased lactate, a product of glycolysis, in the media 24 h post-activation (Figure S5B). Evaluation of extracellular acidification rate (ECAR) revealed enhanced glycolysis, glycolytic capacity, and glycolytic reserve in day 3 stimulated *Pik3cd*$^{E1020K/+}$ OT-1 cells (Figures 4G and S5C), providing evidence that activated PI3Kδ promotes a strong glycolytic profile upon T cell activation.

PI3Kδ hyperactivity promotes a c-Myc, RNA biogenesis, and aminoacyl-tRNA synthesis program

In T cells, c-Myc is another major TF that induces expression of genes encoding glycolytic enzymes (Wang et al., 2011). Notably, Myc target genes were the most enriched hallmark set identified by GSEA in *Pik3cd*$^{E1020K/+}$ transcriptomes, along with multiple Myc-induced pathways, including ribosomal biogenesis, aminoacyl-tRNA synthase, RNA transport, and the unfolded protein response (Figures 4B, 4C, S5D, and S5E). Myc-binding sites were also the most enriched motifs within positively regulated DEG promoters in *Pik3cd*$^{E1020K/+}$ CD8^{+} T cells (Figure 4E). c-Myc protein was induced similarly in both WT and *Pik3cd*$^{E1020K/+}$ cells 4 h post-stimulation. However, c-Myc expression diminished progressively in WT cultures but remained high in *Pik3cd*$^{E1020K/+}$ cells (Figure 4H, left panel). *Pik3cd*$^{E1020K/+}$ OT-1 cells also exhibited elevated phospho-c-Myc^{S62} post-stimulation (Figure 4H, right panel), suggestive of enhanced c-Myc activity (Preston et al., 2015).

c-Myc induces expression of a broad range of nutrient transporters (Marchingo et al., 2020; Wang et al., 2011); transcripts encoding numerous amino acid (AA) transporters were elevated in *Pik3cd*$^{E1020K/+}$cells, including *Slc7a5* (leucine, methionine, and tryptophan), *Slc1a5* (glutamine, serine, threonine, and alanine), and *Slc7a1* (arginine and lysine) (Figure 4I). Surface CD98, a component of the large neutral AA transporter LAT1, and CD71, the transferrin receptor, were also increased (Figure S5F). c-Myc also promotes synthesis of electron transport chain

(C) GSEA plots of enrichment for selected hallmark pathways.
(D) HIF-1α in T cells (n = 2, representative blot).
(E) HGT for top 10 enriched MSigDB TF target sets among negatively (blue) and positively (red) regulated DEGs. Size and color saturations are proportional to gene count (total shown) and p value, respectively.
(F) OT-1 cells expressing GLUT1 (n = 4).
(G) ECAR on day 3 activated OT-1 cells in response to exogenous glucose, oligomycin, and 2-deoxy-D-glucose (n = 3, representative example).
(H) OT-1 cells were peptide stimulated; shown are total c-Myc (left) and phospho-c-Myc^{S62} (right) (n = 3, representative example).
(I) Heatmap of log10 transcripts per million (TPM) +1 values for selected nutrient transporter genes (hierarchically clustered rows).
(J–L) OCR and ECAR measured on day 3 activated OT-1 cells in response to oligomycin, fluoro-carbonyl cyanide phenylhydrazone (FCCP), and antimycin A plus rotenone (AA/R) in the presence of glucose (n = 3, representative example). (J) OCR. (K) Basal ECAR. (L) Ratio of basal OCR/ECAR.
Graphs show mean ± SEM. Graph (K) shows mean ± SEM, $p < 0.0001$. See Figure S5 and Table S1.

components that augment mitochondrial ATP production to match the high metabolic demands of activated lymphocytes (Singh et al., 2019). Genes involved in oxidative phosphorylation were highly enriched in activated *Pik3cd*$^{E1020K/+}$ CD8$^+$ T cells (Figures 4B and 4C). Accordingly, extracellular flux analyses revealed that activated *Pik3cd*$^{E1020K/+}$ OT-1 cells had heightened basal oxidative consumption rates (OCRs), increased maximal respiration, and elevated ATP-dependent respiration compared to WT (Figures 4J and S5G). Strikingly, the bioenergetic profile (OCR versus ECAR) showed that activated *Pik3cd*$^{E1020K/+}$ OT-1 cells were metabolically more energetic than WT counterparts (Figures 4K, 4L, and S5H). Thus, hyperactive PI3Kδ leads to a heightened metabolic state reflective of aberrantly activated transcriptional modules downstream of c-Myc and mTORC1.

Activated *Pik3cd*$^{E1020K/+}$ CD8$^+$ T cells exhibit pronounced IL-2/STAT5 signatures

In conjunction with elevated mTOR and c-Myc signatures, GSEA uncovered an enrichment of genes downstream of IL-2/STAT5 in *Pik3cd*$^{E1020K/+}$ CD8$^+$ T cells (Figures 4B and 5A). These included pro-inflammatory genes, such as *Il2ra*, *Ccr4*, *Nfil3*, and *Csf2*, and regulatory genes, such as *Cish*, *Socs1*, *Socs3*, *Tnfsf10* (encoding TRAIL), and *Tnfsf21* (encoding DR6) (Figure 5B). Increased protein was confirmed for several STAT5 targets, including the TF NFIL3 and costimulatory protein TNFRSF9 (CD137), which are both expressed in IL-2 expanded CTLs (Rollings et al., 2018; Figure 5C). *Pik3cd*$^{E1020K/+}$ OT-1 cells also showed elevated BATF3 (Figure 5C), a TF recently implicated in CD8$^+$ T cell memory (Ataide et al., 2020). NFIL3 and BATF3 were also elevated in CD8$^+$ T cells from patients with APDS compared to healthy controls following stimulation (Figure 5D).

The IL-2R comprises IL-2Rβ:IL-2Rγ heterodimers and CD25, the high-affinity IL-2Rα chain, which is induced by both TCR and IL-2 signals (Ross and Cantrell, 2018). Stimulation with optimal doses of OVA$_{257-264}$ peptide (10 nM) led to similar or only mildly increased CD25 in *Pik3cd*$^{E1020K/+}$ OT-1 cells compared to WT. However, under low peptide dose or altered peptide stimulation, *Pik3cd*$^{E1020K/+}$ OT-1 cells had elevated and sustained CD25 expression (Figure 5E). Stimulated CD8$^+$ T cells from patients with APDS also exhibited elevated CD25 compared to healthy controls (Figure 5D). *Pik3cd*$^{E1020K/+}$ CD8$^+$ T cells also produced increased IL-2 (Figure S6A).

IL-2R engagement results in phosphorylation and activation of the STAT5 TF as well as activation of mTORC1 (Ross and Cantrell, 2018). To address whether PI3Kδ hyperactivity also promotes IL-2 responsiveness, OT-1 cells were stimulated under conditions where WT and *Pik3cd*$^{E1020K/+}$ cells expressed similar CD25 (Figure 5E, left panels), washed, and rested. Upon restimulation with IL-2, *Pik3cd*$^{E1020K/+}$ OT-1 cells exhibited elevated and sustained pSTAT5^{Y694} and pS6$^{S240/244}$ (Figure 5F). To eliminate differential contributions of autocrine IL-2, we included a blocking anti-mouse IL-2 antibody and added saturating levels of exogenous human IL-2 in the cultures prior to resting and re-exposure to IL-2. Under these conditions, *Pik3cd*$^{E1020K/+}$ CD8$^+$ T cells maintained elevated pS6$^{S240/244}$ that increased even further following IL-2 stimulation (Figure 5G). Thus, *Pik3cd*$^{E1020K/+}$ cells exhibit robust and sustained responses to IL-2.

Activated PI3Kδ drives effector programs at the expense of memory phenotypes

CD8$^+$ T cells commit to an effector fate when cultured with IL-2 but differentiate along a memory-like program when cultured with IL-15, a related cytokine that shares two receptor subunits and, at least qualitatively, mobilizes similar signaling pathways (Cornish et al., 2006; van der Windt et al., 2012). To ask whether activated PI3Kδ differentially affects responses to these cytokines, OT-1 cells were peptide-stimulated for 3 days and then cultured with either IL-2 or IL-15 (see Figure S7A).

After expansion in IL-2, WT cultures contained large blasting cells and expressed cytolytic effector molecules and the activation marker CD69 but reduced CD62L. In contrast, WT cells grown in IL-15 were smaller, with low GzmB and elevated CD62L, consistent with a memory-like phenotype (Figures 6A and S7B). Regardless of culture conditions, *Pik3cd*$^{E1020K/+}$ OT-1 cells were larger than WT counterparts; *Pik3cd*$^{E1020K/+}$ CD8$^+$ T cells cultured in IL-15 had similar diameters as WT cells grown in IL-2 (Figure S7B). In addition, IL-15-cultured *Pik3cd*$^{E1020K/+}$ CD8$^+$ T cells exhibited effector-like features, including elevated GzmB and CD69, and low CD62L (Figures 6A and S7B), despite equivalent or higher expression of CD215, the IL-15Rα, compared to WT cells (Figure S7C). *Pik3cd*$^{E1020K/+}$ CD8$^+$ cells proliferated more than WT; their proliferation in IL-15 resembled that of WT cells expanded in IL-2 (Figure S7D). Differences in cell death between WT and *Pik3cd*$^{E1020K/+}$ CD8$^+$ T cells were also less pronounced after growth in cytokines, particularly in IL-15 (Figure S7E). *Pik3cd*$^{E1020K/+}$ CD8$^+$ T cells, therefore, failed to acquire characteristics of T_{CM} cells, even when provided a stimulus that normally pushes toward memory fates.

Principal-component analysis (PCA) of transcripts after *in vitro* culture with IL-2 or IL-15 confirmed that transcriptomes of *Pik3cd*$^{E1020K/+}$ cells exposed to IL-15 broadly resembled those of either WT or *Pik3cd*$^{E1020K/+}$ cells exposed to IL-2, as evidenced by increased values on the principal component (PC)2 axis (Figure 6B). This directionality was also seen when comparing WT and *Pik3cd*$^{E1020K/+}$ OT-1 cells cultured in IL-2, with the latter exhibiting greater PC2 directionality (Figure 6B).

We then assembled a master set of DEGs across experimental conditions, split it into 10 hierarchical clusters, and ran HGT against Kyoto Encyclopedia of Genes and Genomes (KEGG) pathways (Figures 6C and 6D). These clusters revealed distinct patterns that underscored biological differences between WT and *Pik3cd*$^{E1020K/+}$ CD8$^+$ T cells. Cluster 4 (red), which contained genes expressed in IL-2-containing cultures, regardless of genotype, was induced by IL-15 only in *Pik3cd*$^{E1020K/+}$ cells. This cluster was enriched for genes involved in cytotoxicity, chemokine signaling, and PI3K signaling (Figure 6D). Cluster 9 was enriched for FoxO signature genes; expression of these genes was depressed in *Pik3cd*$^{E1020K/+}$ cells regardless of culture conditions (Figures 6C and 6D). In contrast, cluster 2 was enriched for genes involved in ribosomal biogenesis and AA biosynthesis; expression of these genes was exaggerated in *Pik3cd*$^{E1020K/+}$ cells cultured in either IL-2 or IL-15, but more so in the former (Figures 6C and S7F), supporting amplification of protein synthesis machinery in *Pik3cd*$^{E1020K/+}$ cells.

We next ran HGT against curated sets of effector and memory T cell transcripts derived from mouse models of viral infection

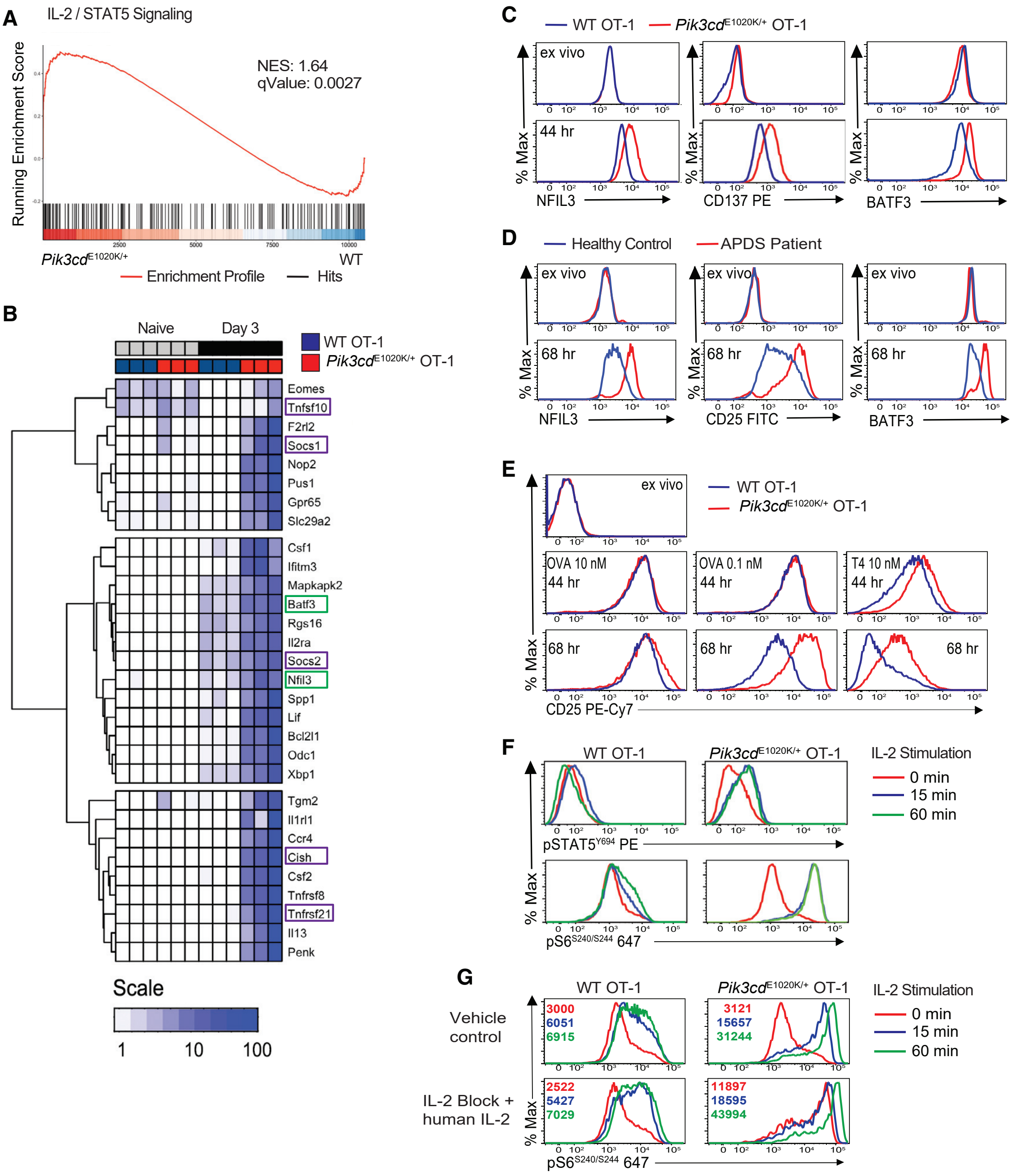

Figure 5. Enhanced IL-2/STAT5 signature and IL-2 sensitivity in activated *Pik3cd*$^{E1020K/+}$ OT-1 T cells

(A) GSEA for IL-2-STAT5 hallmark pathway.

(B) Heatmap of log10 TPM + 1 values for genes (hierarchically clustered rows) defined as core enriched elements in (A). Green boxes, TFs; purple boxes, regulatory genes.

(C) NFIL3, CD137, and BATF3 in peptide-stimulated OT-1 cells (n = 4, representative example).

(D) CD25, BATF3, and NFIL3 in T cells from healthy controls and patients with APDS (on treatment with Sirolimus) stimulated with anti-CD3 plus anti-CD28 (n = 3, representative example).

(E) CD25 on OT-1 cells stimulated with $OVA_{257–264}$ or T4 (n = 4, representative example).

(F) OT-1 cells stimulated with 10 nM $OVA_{257–264}$ for 3 days, re-stimulated with IL-2, and evaluated for $pSTAT5^{Y694}$ and $pS6^{S240/244}$ (n = 3, representative example).

(G) $pS6^{S240/244}$ in OT-1 cells were stimulated with 10 nM $OVA_{257–264}$ with or without vehicle control or anti-IL-2 and exogenous human IL-2 and then washed, rested, and re-stimulated with IL-2 (MFI in histogram; n = 4, representative example).

See Figure S6 and Table S1.

(Kaech et al., 2002; Luckey et al., 2006). Cluster 6 (blue), which contained genes highly expressed in WT IL-15 cultures and reduced in *Pik3cd*$^{E1020K/+}$ cells, was enriched for genes associated with T cell memory. In contrast, cluster 4, which was expressed by IL-2-treated cultures of both genotypes, as well as *Pik3cd*$^{E1020K/+}$ IL-15 cultures, was enriched for genes associated with effector T cells (Figures 6C, 6E, and S7G).

WT effector CD8$^+$ T cells display reduced oxidative metabolism with little to no spare respiratory capacity (SRC) compared to memory cells (Gattinoni et al., 2009; van der Windt et al., 2012), (Figures 6F and 6G). Compared to WT effectors, *Pik3cd*$^{E1020K/+}$ OT-1 cells cultured in IL-2 exhibited decreased maximal respiration and SRC (Figures 6F and S7H); these differences were even more profound in IL-15 cultures (Figures 6G and S7I). Thus, *Pik3cd*$^{E1020K/+}$ OT-1 cells differentiated in the presence of either IL-2 or IL-15 display impaired cellular fitness with reduced energy reserves compared to WT counterparts.

Memory cells have increased fatty acid oxidation, which is facilitated by expression of lysosomal acid lipase (LAL) (O'Sullivan et al., 2014); WT OT-1 cells cultured in IL-15 expressed more LAL than IL-2-differentiated cells. However, *Pik3cd*$^{E1020K/+}$ CD8$^+$ T cells differentiated under either IL-2 effector-like or IL-15 memory-like conditions expressed low LAL (Figure 6H). Collectively, these data provide evidence that IL-15-cultured *Pik3cd*$^{E1020K/+}$ OT-1 cells fail to acquire characteristics of memory-like cells and instead have transcriptional and metabolic programs resembling effector cells.

Activated PI3Kδ alters the chromatin landscape of activated CD8$^+$ T cells

To provide insight into dysregulated effector/memory programs in *Pik3cd*$^{E1020K/+}$ CD8$^+$ T cells, we assessed their chromatin landscape. High-resolution profiling by assay for transposase-accessible chromatin with high-throughput sequencing (ATAC-seq) revealed divergence in areas of open chromatin between activated WT and *Pik3cd*$^{E1020K/+}$ CD8$^+$ T cells, with 2,637 unique peaks detected in the former and 1,389 unique peaks in the latter (Figure 7A). Unbiased *de novo* DNA motif analysis uncovered distinct patterns of TF binding sites within these accessible regions. Consistent with their activated phenotype, JunB-binding sites were the most enriched motif in CD8$^+$ T cells from *Pik3cd*$^{E1020K/+}$ mice (Figure 7B; Yukawa et al., 2020). Recognition sites for STAT5 were also among the top enriched motifs in *Pik3cd*$^{E1020K/+}$ CD8$^+$ T cells (Figure 7B), with enhanced accessibility at the classic STAT5 targets *Socs1*, *Cish*, and *Ccr4*, as well as *Batf3* and *Ccl3* (Figures 7C and S8A). Together, these data demonstrate that post-activation, the chromatin landscapes of WT and *Pik3cd*$^{E1020K/+}$ CD8$^+$ T cells diverged, with the latter displaying evidence for enhanced STAT5 activity.

Although IL-2 is required for both CD8$^+$ memory and effector T cells, high CD25 expression is linked to effector differentiation (Kalia and Sarkar, 2018; Pipkin et al., 2010). The early difference in STAT5 markings suggested that increased IL-2 production and responsiveness of *Pik3cd*$^{E1020K/+}$ CD8$^+$ T cells may contribute to the inability to differentiate along a memory pathway. To address this, we cultured OT-1 cells with blocking anti-IL-2 antibodies or a PI3Kδ inhibitor for 3 days and then washed out the inhibitors and examined differentiation into effector- or memory-like cells upon exposure to IL-2 or IL-15 (Figure S8B, outline). Under these conditions, *Pik3cd*$^{E1020K/+}$ OT-1 cells exhibited reduced GzmB and CD25 and re-expressed CD62L by day 3 of activation (Figure 7D, top panel, and Figures S8C and S8D). Nonetheless, when CAL-101 (Figure S8D) or anti-IL-2 (Figure 7D, middle panels) was washed out and cells were exposed to IL-2, *Pik3cd*$^{E1020K/+}$ OT-1 cells again expressed higher GzmB and reduced CD62L relative to WT, confirming enhanced IL-2 responses.

In contrast, when the PI3Kδ inhibitor or anti-IL-2 was removed and cells were exposed to IL-15, *Pik3cd*$^{E1020K/+}$ CD8$^+$ T cells exhibited more memory-like phenotypes, with reduced GzmB and increased CD62L (Figures 7D and S8D, bottom panels). Thus, early exposure of *Pik3cd*$^{E1020K/+}$ CD8$^+$ T cells to IL-2 results in changes in chromatin and prevents IL-15-induced differentiation into memory-like cells.

Activated PI3Kδ CD8$^+$ T cells lose TCF1-associated chromatin accessibility

Further evaluation of day 3 ATAC-seq data revealed differentially enriched DNA motifs specifically in WT CD8$^+$ T cells. These included increases in motifs for Brother of the Regulator of Imprinted Sites/CCCTC-binding factor (BORIS/CTCF), which bind to boundaries of chromatin topologically associating domains to define regulatory modules, supporting an early divergence in the overall chromatin organization between activated WT and *Pik3cd*$^{E1020K/+}$ cells (Figure 7B, lower panel). Although we observed differential markings of KLF family motifs (Figure S8E), FoxO-binding sites were only modestly enriched (p = 1e-2). However, recognition sites for TCF1 were highly enriched within accessible regions, specifically in WT CD8$^+$ T cells (Figure 7B). Parallel findings were observed in antigen-specific cells 1 week post-influenza X31-OVA infection, including differences in motifs for BORIS, IRF1, and TCF1 in WT compared to *Pik3cd*$^{E1020K/+}$ cells (Figures S8F and S8G).

TCF1 is highly expressed in both naive and T_{CM} cells, and TCF1-binding sites are enriched in accessible regions shared between naive and memory cells after acute viral infection (Scott-Browne et al., 2016). Indeed, a majority of accessible regions found only *in-vitro*-activated WT CD8$^+$ T cells overlapped with peaks previously identified in naive and memory CD8$^+$ T cells (Shih et al., 2016), unlike those detected only in *Pik3cd*$^{E1020K/+}$ CD8$^+$ T cells (Figures 7F and S8H). Thus, hyperactive PI3Kδ instigates a loss of chromatin accessibility at loci controlled by TCF1, a key pro-memory TF. Collectively, our findings position PI3Kδ as a key integrator that drives transcriptional, chromatin, and metabolic changes that promote differentiation of effector cells at the expense of central memory.

DISCUSSION

Effector cell differentiation and the development of memory are critical for proper adaptive immunity; understanding these processes is therefore of major importance for improving responses to vaccination and infection. Using a mouse model expressing activated PI3Kδ and T cells from patients with APDS, we provide evidence that PI3Kδ is central to a network of factors driving effector T cell differentiation while preventing acquisition of

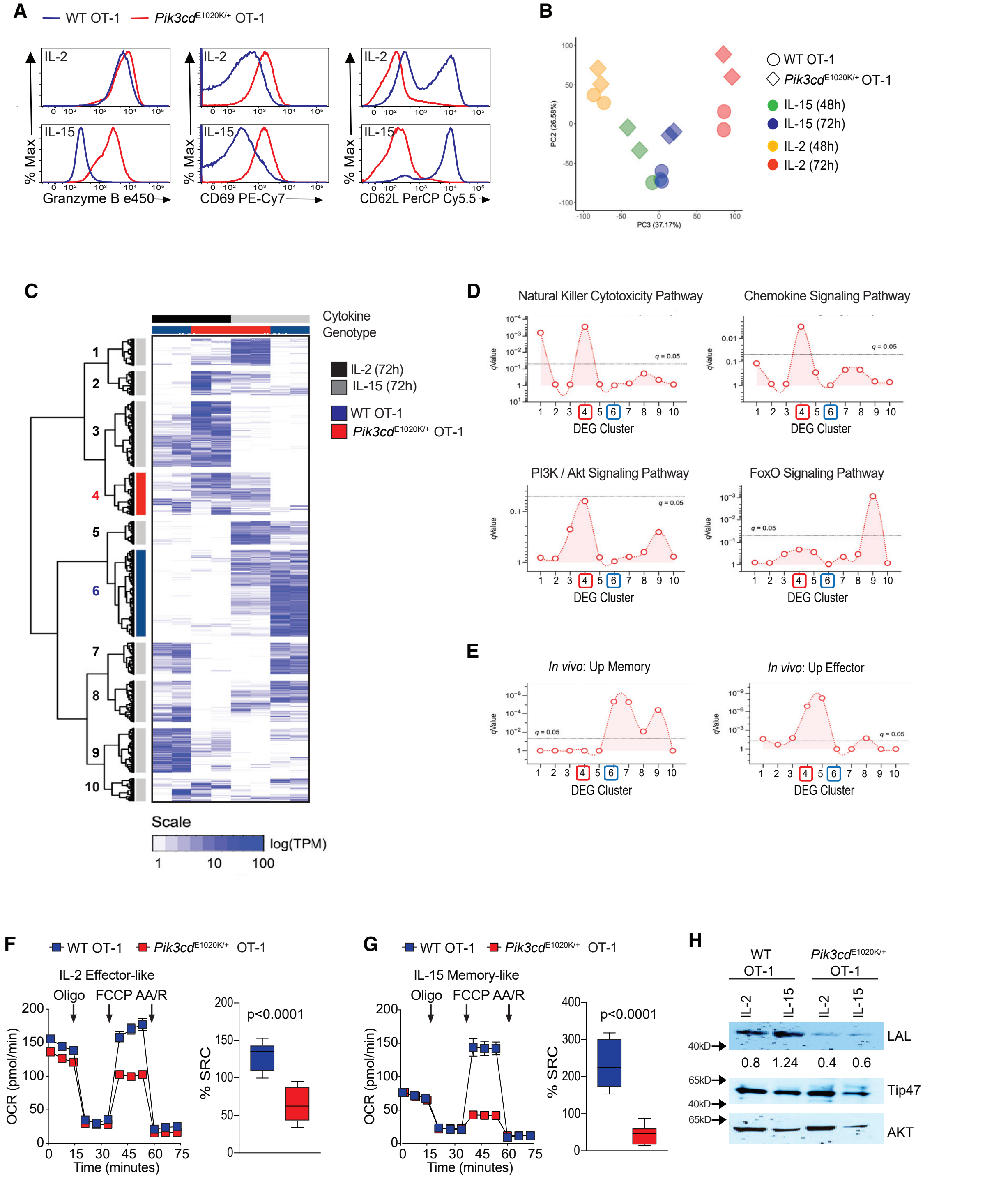

Figure 6. IL-15 differentiated *Pik3cd*$^{E1020K/+}$ OT-1 CD8^{+} T cells resemble effector cells

(A–E) OT-1 cells activated with OVA$_{257–264}$ for 3 days and then cultured with either IL-2 or IL-15 to generate effector-like or memory-like cells, respectively (see Figure S6A). (A) GzmB, CD69, and CD62L (n = 3, representative flow plots). (B–E) RNA-seq analysis. (B) Principal-component analysis (PCA) of the two most variant TPM data components (WT cells, circles; *Pik3cd*$^{E1020K/+}$, triangles). (C) Heatmap of TPM values and Euclidian clustering for DEGs (rows) and experimental groups (columns). (D and E) Enrichment across 10 row clusters (C) of KEGG pathways (D) or curated CD8^{+} T cell gene sets (E) from http://www.gsea-msigdb.org/gsea/index.jsp.

(F and G) OCR under basal conditions and in response to mitochondrial inhibitors oligomycin, FCCP, and antimycin A plus rotenone and calculated as the percent SRC in cells differentiated in IL-2 (F) and IL-15 (G) (representative data, n = 4).

(H) LAL, Tip47, and AKT from IL-2 or IL-15 cultured cells (n = 2). Graphs (F and G) show mean ± SEM, percent SRC $p < 0.0001$.

See Figure S7 and Table S2.

CellPress
OPEN ACCESS

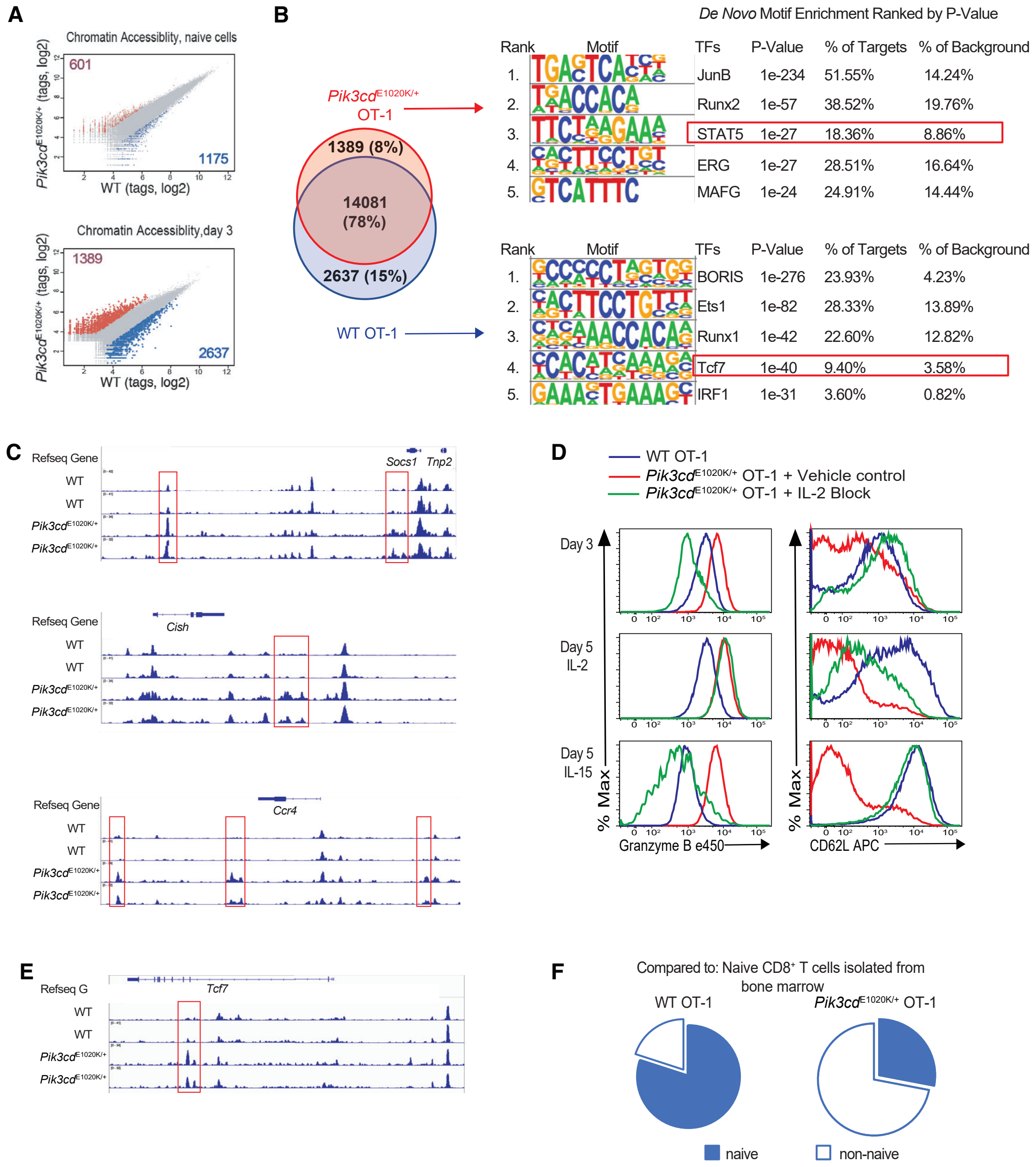

Figure 7. IL-2 drives early effector trajectory of *Pik3cd*$^{E1020K/+}$ CD8$^+$ cells

(A–C) ATAC-seq analysis of naive and day 3 activated OT-1 cells (n = 2). (A) Normalized ATAQ-seq tag density comparing chromatin accessibility (fold change > 2; false discovery rate [FDR] < 0.05; top: naïve; bottom: day 3). (B) *De novo* unbiased motif discovery using HOMER. JunB- and STAT5-enriched motifs noted in *Pik3cd*$^{E1020K/+}$ (top) and Tcf7 enriched motif in WT cells (lower). (C) ATAC-seq genomic tracks across IL-2/STAT5 signature genes. Red boxes highlight differences in chromatin accessibility.

(D) OT-1 cells were stimulated with peptide with or without anti-IL-2 for 3 days, washed, and cultured in either IL-2 or IL-15 (GzmB and CD62L, representative example; n = 3).

(E) ATAC-seq profiles on *Tcf7*.

(F) Pie chart of overlapping unique ATAC-seq peaks from WT and *Pik3cd*$^{E1020K/+}$ day 3 activated OT-1 cells versus BM naive CD8$^+$ T cells.

See Figure S8.

T_{CM} phenotypes. Our results have implications both for understanding phenotypes of patients with APDS and the regulation of effector and memory responses.

A large proportion of effector T cells are short-lived and terminally differentiated, providing immediate, acute function, then undergoing apoptosis (Backer et al., 2018). Although

PI3K is usually associated with cell survival, T cells from both patients with APDS and *Pik3cd*$^{E1020K/+}$ mice exhibit heightened TCR-induced apoptosis (Angulo et al., 2013; Bier et al., 2019), resulting in part from early and increased expression of *Fasl*, a known target of IL-2-STAT5 (Ross and Cantrell, 2018), AKT, and HIF-1α-pathways (Finlay et al., 2012; Macintyre et al., 2011). We now show that FoxO1 plays a major role in restraining *Fasl* expression in CD8^{+} T cells. Recent data indicate that Fas promotes terminal effector differentiation of naive cells in the presence of activated cells expressing FasL (Klebanoff et al., 2016). Thus, increased FasL may also potentiate effector differentiation, underscoring positive feedback in this pathway. Furthermore, as FasL-Fas interactions require a second "competency signal" to induce apoptosis in activated cells (Combadière et al., 1998), our results also implicate PI3Kδ activity in Fas signaling. Nonetheless, Fas-mediated cell death is likely not the only cause of diminished viability, as blocking FasL or AKT did not completely prevent PI3Kδ-driven cell death. Furthermore, *Pik3cd*$^{E1020K/+}$ CD8^{+} T cells also show enhanced proliferation, particularly to cytokines, which may account for their paradoxical increased cell numbers over time.

Our data highlight several major pathways affected by activated PI3Kδ that contribute to heightened effector function. First, despite data arguing that mTORC1 activation and Myc expression are independent of PI3Kδ in CD8^{+} T cells (Finlay et al., 2009; Spinelli et al., 2021), our findings indicate that activated PI3Kδ is sufficient to drive these processes, perhaps due to increased IL-2 signals. Increased phosphorylation of AKT^{S473} also points to amplified mTORC2 signals, an idea supported by similar observations in *Pten*-deficient regulatory CD4^{+} T cells (Shrestha et al., 2015).

Notably, the most dysregulated pathway in *Pik3cd*$^{E1020K/+}$ CD8^{+} T cells was "Myc targets." Data suggest that c-Myc is a broad amplifier of transcription (Nie et al., 2012) that itself is increased by mTORC1. Increased Myc likely contributes to the overall heightened activation of *Pik3cd*$^{E1020K/+}$ T cells, with a more active, synthetic effector phenotype; increased blastogenesis; and elevated Myc-regulated transcripts associated with AA transport, ribosomal biogenesis, tRNA biosynthesis, and the unfolded protein response (Marchingo et al., 2020). In turn, increased AA transporters may help fuel mTOR activation, which also positively impacts biosynthesis and cell size.

Strikingly, we observed an exaggerated IL-2-STAT5 signature in *Pik3cd*$^{E1020K/+}$ CD8^{+} T cells; this strongly correlates with IL-2 signatures in a recent proteomic analyses of CTLs (Rollings et al., 2018). Although both IL-2 and IL-15 transduce signals via the IL-2R common γ-chain, IL-2 drives increased and prolonged pS6 compared to IL-15 (Cornish et al., 2006). It is therefore of note that *Pik3cd*$^{E1020K/+}$ CD8^{+} T cells displayed heightened and sustained pS6 in response to multiple signals. Our data further suggest that accentuated IL-2 signaling prevents *Pik3cd*$^{E1020K/+}$cells from responding appropriately to IL-15. Nonetheless, early addition of high-dose IL-2 to WT cultures was not sufficient to prevent subsequent "memory-like" differentiation in IL-15 (unpublished observations), suggesting that activated PI3Kδ coalesces multiple signals to promote effector phenotypes.

Although activated *Pik3cd*$^{E1020K/+}$ CD8^{+} T cells show both heightened glycolysis and oxygen consumption, further culture of *Pik3cd*$^{E1020K/+}$ cells led to decreased oxidative respiration, with a loss of SRC, which is thought to facilitate rapid expansion and long-term survival in memory cells (van der Windt et al., 2012). Reductions in oxidative phosphorylation were also recently seen in another *Pik3cd*$^{E1020K/+}$ mouse strain (Jia et al., 2021), albeit after a different length of activation. Our results suggest that *Pik3cd*$^{E1020K/+}$ CD8^{+} T cells are initially more active bioenergetically, but then may "burn out," losing their ability to maintain oxidative metabolism for cellular energy, and failing to undergo metabolic reprogramming of memory cells. Why *Pik3cd*$^{E1020K/+}$ mice maintain and even increase a population of antigen-specific cells resembling recently described LLEC is less clear, but is reminiscent of increased T_{EM} and EBV-specific cells in patients with APDS and may result from the ability of IL-15 to induce proliferation and effector-like phenotypes in activated PI3Kδ cells. Thus, our data indicate that proper regulation of PI3K activity is critical to balance long-lived effector versus central memory populations and may be an essential signal for generating LLECs. It is of note that FoxO1-deficient CD8^{+} T cells also show decreased T_{CM} cells and true CD127^{+} T_{EM} cells yet increased CD127^{lo} LLECs (Milner et al., 2020). Whether these cells maintain long-term responses during chronic infections is an important question. Indeed, cells with a phenotype similar to LLECs poorly restrain chronic LCMV clone 13 infection (Nolz and Harty, 2011). Also of note, both our initial description (Lucas et al., 2014),and subsequent work (Cannons et al., 2018; Edwards et al., 2019; Lucas et al., 2014) highlight how T cells from patients with APDS exhibit characteristics of senescence, including increased CD57 and shortened telomeres. We propose these phenotypes are features of *Pik3cd*$^{E1020K/+}$ CD8^{+} T cell differentiation into "supereffectors" that cannot maintain cellular fitness. Their poor proliferative recall responses combined with a loss of naive T cells over time (Preite et al., 2018), as well as effects on expression of molecules such as 2B4 and other immune cells that affect responses to EBV (Cannons et al., 2018; Edwards et al., 2019; Stark et al., 2018), may ultimately compromise the ability to contain certain chronic infections.

Similar to *Pik3cd*$^{E1020K/+}$ CD8^{+} T cells, those lacking TCF1 do not develop T_{CM} cells after acute infection (Zhou et al., 2010). TCF1-deficient cells are also driven to a terminal-effector-like phenotype (Chen et al., 2019) and fail to maintain long-lived responses in chronic infections (Snell et al., 2018; Utzschneider et al., 2016; Wu et al., 2016). TCF1 is expressed in naive cells but suppressed in a large portion of CD8^{+} T effector cells in acute infection, while it is maintained in T_{CM} cells (Lugli et al., 2020) and CD127^{+} T_{EM} cells (Milner et al., 2020). In culture, TCF1 expression is suppressed in a portion of activated cells and is a marker of asymmetric cell division, a PI3K-dependent process (Lin et al., 2015). Indeed, we find that activated PI3Kδ plays a critical role in suppressing TCF1, *in vitro* and *in vivo*, as well as in *in-vitro*-activated CD8^{+} T cells from patients with APDS.

It is therefore of interest that TCF1-associated chromatin modifications are shared by naive and memory cells (Scott-Browne et al., 2016), which retain a more open/poised chromatin conformation compared to effector cells (Gray et al., 2017). In this

article, we defined epigenetics as the study of chromatin states that regulate transcriptional activities without alteration of DNA sequences. Our ATAC-seq data argue that activated PI3Kδ cells rapidly lose TCF1-associated markings both *in vitro* and *in vivo*. The enrichment of BORIS/CTCF motifs within differentially accessible regions further supports a divergence in the overall chromatin architecture between activated WT and *Pik3cd*$^{E1020K/+}$ CD8^{+} T cells. Thus, PI3Kδ plays a fundamental role in reorienting the chromatin landscape in a manner that favors effector differentiation and excludes memory specifications.

Multiple interconnected factors have been shown to regulate generation of T_{CM} cells, including TFs such as FoxO1, TCF1, KLF2, Blimp1, and BACH2; microRNAs such as miR17-92; chromatin remodeling factors such as Ezh2; and metabolic regulators and signaling molecules such as mTORC1, BCAP, and AKT (Araki et al., 2009; Gray et al., 2017; Hand et al., 2010; Kallies et al., 2009; Kim et al., 2013; Lin et al., 2015; Hess Michelini et al., 2013; Roychoudhuri et al., 2016; Singh et al., 2018; Wu et al., 2012). Remarkably, many of these molecules are regulated by or connected to PI3Kδ. Thus, although we think of TFs as master regulators of differentiation, our data suggest that PI3Kδ is a master regulator integral to a network of interacting factors that drive effector versus memory phenotypes and potentially long-term responses.

APDS is associated with multiple recurrent infections; ~40% of patients exhibit high EBV and/or CMV viral titers (Coulter et al., 2017). Our findings provide insight into some of these phenotypes where CD8^{+} T cells are propelled toward an effector phenotype associated with increased cell death and "metabolic fatigue," preventing the generation of T_{CM} cells during acute infection and perhaps a CD8 stem-like progenitor population during chronic infection. Whether these changes affect long-term responses to infection and whether PI3Kδ inhibitors can alter these phenotypes in APDS are important questions. The implications for adoptive T cell therapies, where use of PI3Kδ inhibitors during cell expansion prior to transfer may maintain progenitor and memory pools and thereby extend longevity of therapy, remain important issues to explore.

STAR★METHODS

Detailed methods are provided in the online version of this paper and include the following:

- KEY RESOURCES TABLE
- RESOURCE AVAILABILITY
  - Lead contact
  - Materials availability
  - Additional resources
  - Data and code availability
- EXPERIMENTAL MODELS AND SUBJECT DETAILS
  - Patient samples
  - Mice and infectious models
- METHOD DETAILS
  - Bone marrow chimeras
  - Cell Culture
  - Flow Cytometry
  - Western Blots
  - Metabolic Assays
  - Retroviral transduction
  - RNA-Sequencing and analysis
  - ATAC-Sequencing and analysis
  - Data Analysis
- QUANTIFICATION AND STATISTICAL ANALYSIS.

SUPPLEMENTAL INFORMATION

Supplemental information can be found online at https://doi.org/10.1016/j.celrep.2021.109804.

ACKNOWLEDGMENTS

We thank S. Crotty, Y.S. Choi, and J. Choi for FoxO1 constructs. This work was supported in part by intramural funding from the NIAID, NHGRI, NINDS, and NIAMS. In memory of our colleague R. Handon.

AUTHOR CONTRIBUTIONS

J.L.C., A.V.V., S.M.K., S.P., H.-Y.S., and J.G.-R. designed, performed, and interpreted experiments and edited the manuscript. J.M.R., B.H., T.W., I.T.M., H.S., Z.K., S.G., and R.H. helped perform experiments. H.C.S., D.B.M., J.J.O., P.J.M., and G.U. provided critical reagents and scientific input. J.L.C. and P.L.S. were responsible for project design and manuscript writing.

DECLARATION OF INTERESTS

S.P. is an employee of AstraZeneca and may own stock or stock options. J.G.-R. is an employee of TCR2. The remaining authors declare no competing interests.

Received: October 18, 2020
Revised: July 15, 2021
Accepted: September 16, 2021
Published: October 12, 2021

REFERENCES

Anders, S., McCarthy, D.J., Chen, Y., Okoniewski, M., Smyth, G.K., Huber, W., and Robinson, M.D. (2013). Count-based differential expression analysis of RNA sequencing data using R and Bioconductor. Nat. Protoc. *8*, 1765–1786.

Ando, R., Shima, H., Tamahara, T., Sato, Y., Watanabe-Matsui, M., Kato, H., Sax, N., Motohashi, H., Taguchi, K., Yamamoto, M., et al. (2016). The Transcription Factor Bach2 Is Phosphorylated at Multiple Sites in Murine B Cells but a Single Site Prevents Its Nuclear Localization. J. Biol. Chem. *291*, 1826–1840.

Angulo, I., Vadas, O., Garçon, F., Banham-Hall, E., Plagnol, V., Leahy, T.R., Baxendale, H., Coulter, T., Curtis, J., Wu, C., et al. (2013). Phosphoinositide 3-kinase δ gene mutation predisposes to respiratory infection and airway damage. Science *342*, 866–871.

Araki, K., Turner, A.P., Shaffer, V.O., Gangappa, S., Keller, S.A., Bachmann, M.F., Larsen, C.P., and Ahmed, R. (2009). mTOR regulates memory CD8 T-cell differentiation. Nature *460*, 108–112.

Ataide, M.A., Komander, K., Knöpper, K., Peters, A.E., Wu, H., Eickhoff, S., Gogishvili, T., Weber, J., Grafen, A., Kallies, A., et al. (2020). BATF3 programs CD8^{+} T cell memory. Nat. Immunol. *21*, 1397–1407.

Avery, D.T., Kane, A., Nguyen, T., Lau, A., Nguyen, A., Lenthall, H., Payne, K., Shi, W., Brigden, H., French, E., et al. (2018). Germline-activating mutations in *PIK3CD* compromise B cell development and function. J. Exp. Med. *215*, 2073–2095.

Backer, R.A., Hombrink, P., Helbig, C., and Amsen, D. (2018). The Fate Choice Between Effector and Memory T Cell Lineages: Asymmetry, Signal Integration, and Feedback to Create Bistability. Adv. Immunol. *137*, 43–82.

Bier, J., Rao, G., Payne, K., Brigden, H., French, E., Pelham, S.J., Lau, A., Lenthall, H., Edwards, E.S.J., Smart, J.M., et al. (2019). Activating mutations in PIK3CD disrupt the differentiation and function of human and murine CD4+ T cells. J. Allergy Clin. Immunol. *144*, 236–253.

Buenrostro, J.D., Giresi, P.G., Zaba, L.C., Chang, H.Y., and Greenleaf, W.J. (2013). Transposition of native chromatin for fast and sensitive epigenomic profiling of open chromatin, DNA-binding proteins and nucleosome position. Nat. Methods *10*, 1213–1218.

Cannons, J.L., Yu, L.J., Hill, B., Mijares, L.A., Dombroski, D., Nichols, K.E., Antonellis, A., Koretzky, G.A., Gardner, K., and Schwartzberg, P.L. (2004). SAP regulates T(H)2 differentiation and PKC-theta-mediated activation of NF-kappaB1. Immunity *21*, 693–706.

Cannons, J.L., Qi, H., Lu, K.T., Dutta, M., Gomez-Rodriguez, J., Cheng, J., Wakeland, E.K., Germain, R.N., and Schwartzberg, P.L. (2010). Optimal germinal center responses require a multistage T cell:B cell adhesion process involving integrins, SLAM-associated protein, and CD84. Immunity *32*, 253–265.

Cannons, J.L., Preite, S., Kapnick, S.M., Uzel, G., and Schwartzberg, P.L. (2018). Genetic Defects in Phosphoinositide 3-Kinase δ Influence CD8+ T Cell Survival, Differentiation, and Function. Front. Immunol. *9*, 1758.

Chen, Z., Ji, Z., Ngiow, S.F., Manne, S., Cai, Z., Huang, A.C., Johnson, J., Staupe, R.P., Bengsch, B., Xu, C., et al. (2019). TCF-1-Centered Transcriptional Network Drives an Effector versus Exhausted CD8 T Cell-Fate Decision. Immunity *51*, 840–855.e5.

Combadière, B., Reis e Sousa, C., Trageser, C., Zheng, L.X., Kim, C.R., and Lenardo, M.J. (1998). Differential TCR signaling regulates apoptosis and immunopathology during antigen responses in vivo. Immunity *9*, 305–313.

Corces, M.R., Buenrostro, J.D., Wu, B., Greenside, P.G., Chan, S.M., Koenig, J.L., Snyder, M.P., Pritchard, J.K., Kundaje, A., Greenleaf, W.J., et al. (2016). Lineage-specific and single-cell chromatin accessibility charts human hematopoiesis and leukemia evolution. Nat. Genet. *48*, 1193–1203.

Cornish, G.H., Sinclair, L.V., and Cantrell, D.A. (2006). Differential regulation of T-cell growth by IL-2 and IL-15. Blood *108*, 600–608.

Coulter, T.I., Chandra, A., Bacon, C.M., Babar, J., Curtis, J., Screaton, N., Goodlad, J.R., Farmer, G., Steele, C.L., Leahy, T.R., et al. (2017). Clinical spectrum and features of activated phosphoinositide 3-kinase δ syndrome: A large patient cohort study. J. Allergy Clin. Immunol. *139*, 597–606.e4.

Edwards, E.S.J., Bier, J., Cole, T.S., Wong, M., Hsu, P., Berglund, L.J., Boztug, K., Lau, A., Gostick, E., Price, D.A., et al. (2019). Activating PIK3CD mutations impair human cytotoxic lymphocyte differentiation and function and EBV immunity. J. Allergy Clin. Immunol. *143*, 276–291.e6.

Finlay, D.K., Sinclair, L.V., Feijoo, C., Waugh, C.M., Hagenbeek, T.J., Spits, H., and Cantrell, D.A. (2009). Phosphoinositide-dependent kinase 1 controls migration and malignant transformation but not cell growth and proliferation in PTEN-null lymphocytes. J. Exp. Med. *206*, 2441–2454.

Finlay, D.K., Rosenzweig, E., Sinclair, L.V., Feijoo-Carnero, C., Hukelmann, J.L., Rolf, J., Panteleyev, A.A., Okkenhaug, K., and Cantrell, D.A. (2012). PDK1 regulation of mTOR and hypoxia-inducible factor 1 integrate metabolism and migration of CD8+ T cells. J. Exp. Med. *209*, 2441–2453.

Gattinoni, L., Zhong, X.S., Palmer, D.C., Ji, Y., Hinrichs, C.S., Yu, Z., Wrzesinski, C., Boni, A., Cassard, L., Garvin, L.M., et al. (2009). Wnt signaling arrests effector T cell differentiation and generates CD8+ memory stem cells. Nat. Med. *15*, 808–813.

Gomez-Rodriguez, J., Meylan, F., Handon, R., Hayes, E.T., Anderson, S.M., Kirby, M.R., Siegel, R.M., and Schwartzberg, P.L. (2016). Itk is required for Th9 differentiation via TCR-mediated induction of IL-2 and IRF4. Nat. Commun. *7*, 10857.

Gray, S.M., Amezquita, R.A., Guan, T., Kleinstein, S.H., and Kaech, S.M. (2017). Polycomb Repressive Complex 2-Mediated Chromatin Repression Guides Effector CD8+ T Cell Terminal Differentiation and Loss of Multipotency. Immunity *46*, 596–608.

Hand, T.W., Cui, W., Jung, Y.W., Sefik, E., Joshi, N.S., Chandele, A., Liu, Y., and Kaech, S.M. (2010). Differential effects of STAT5 and PI3K/AKT signaling on effector and memory CD8 T-cell survival. Proc. Natl. Acad. Sci. USA *107*, 16601–16606.

Hashimoto, M., Im, S.J., Araki, K., and Ahmed, R. (2019). Cytokine-Mediated Regulation of CD8 T-Cell Responses During Acute and Chronic Viral Infection. Cold Spring Harb. Perspect. Biol. *11*, a028464.

Heinz, S., Benner, C., Spann, N., Bertolino, E., Lin, Y.C., Laslo, P., Cheng, J.X., Murre, C., Singh, H., and Glass, C.K. (2010). Simple combinations of lineage-determining transcription factors prime cis-regulatory elements required for macrophage and B cell identities. Mol. Cell *38*, 576–589.

Hess Michelini, R., Doedens, A.L., Goldrath, A.W., and Hedrick, S.M. (2013). Differentiation of CD8 memory T cells depends on Foxo1. J. Exp. Med. *210*, 1189–1200.

Jeannet, G., Boudousquié, C., Gardiol, N., Kang, J., Huelsken, J., and Held, W. (2010). Essential role of the Wnt pathway effector Tcf-1 for the establishment of functional CD8 T cell memory. Proc. Natl. Acad. Sci. USA *107*, 9777–9782.

Jia, Y., Yang, Q., Wang, Y., Li, W., Chen, X., Xu, T., Tian, Z., Feng, M., Zhang, L., Tang, W., et al. (2021). Hyperactive PI3Kδ predisposes naive T cells to activation via aerobic glycolysis programs. Cell. Mol. Immunol. *18*, 1783–1797.

Jonsson, H., Allen, P., and Peng, S.L. (2005). Inflammatory arthritis requires Foxo3a to prevent Fas ligand-induced neutrophil apoptosis. Nat. Med. *11*, 666–671.

Kaech, S.M., Hemby, S., Kersh, E., and Ahmed, R. (2002). Molecular and functional profiling of memory CD8 T cell differentiation. Cell *111*, 837–851.

Kalia, V., and Sarkar, S. (2018). Regulation of Effector and Memory CD8 T Cell Differentiation by IL-2-A Balancing Act. Front. Immunol. *9*, 2987.

Kallies, A., Xin, A., Belz, G.T., and Nutt, S.L. (2009). Blimp-1 transcription factor is required for the differentiation of effector CD8(+) T cells and memory responses. Immunity *31*, 283–295.

Kapnick, S.M., Stinchcombe, J.C., Griffiths, G.M., and Schwartzberg, P.L. (2017). Inducible T Cell Kinase Regulates the Acquisition of Cytolytic Capacity and Degranulation in CD8+ CTLs. J. Immunol. *198*, 2699–2711.

Kerdiles, Y.M., Beisner, D.R., Tinoco, R., Dejean, A.S., Castrillon, D.H., DePinho, R.A., and Hedrick, S.M. (2009). Foxo1 links homing and survival of naive T cells by regulating L-selectin, CCR7 and interleukin 7 receptor. Nat. Immunol. *10*, 176–184.

Kim, M.V., Ouyang, W., Liao, W., Zhang, M.Q., and Li, M.O. (2013). The transcription factor Foxo1 controls central-memory CD8+ T cell responses to infection. Immunity *39*, 286–297.

Klebanoff, C.A., Scott, C.D., Leonardi, A.J., Yamamoto, T.N., Cruz, A.C., Ouyang, C., Ramaswamy, M., Roychoudhuri, R., Ji, Y., Eil, R.L., et al. (2016). Memory T cell-driven differentiation of naive cells impairs adoptive immunotherapy. J. Clin. Invest. *126*, 318–334.

Langmead, B., Trapnell, C., Pop, M., and Salzberg, S.L. (2009). Ultrafast and memory-efficient alignment of short DNA sequences to the human genome. Genome Biol. *10*, R25.

Liberzon, A., Birger, C., Thorvaldsdóttir, H., Ghandi, M., Mesirov, J.P., and Tamayo, P. (2015). The Molecular Signatures Database (MSigDB) hallmark gene set collection. Cell Syst. *1*, 417–425.

Lin, W.H., Adams, W.C., Nish, S.A., Chen, Y.H., Yen, B., Rothman, N.J., Kratchmarov, R., Okada, T., Klein, U., and Reiner, S.L. (2015). Asymmetric PI3K Signaling Driving Developmental and Regenerative Cell Fate Bifurcation. Cell Rep. *13*, 2203–2218.

Lucas, C.L., Kuehn, H.S., Zhao, F., Niemela, J.E., Deenick, E.K., Palendira, U., Avery, D.T., Moens, L., Cannons, J.L., Biancalana, M., et al. (2014). Dominant-activating germline mutations in the gene encoding the PI(3)K catalytic subunit p110δ result in T cell senescence and human immunodeficiency. Nat. Immunol. *15*, 88–97.

Luckey, C.J., Bhattacharya, D., Goldrath, A.W., Weissman, I.L., Benoist, C., and Mathis, D. (2006). Memory T and memory B cells share a transcriptional

CellPress
OPEN ACCESS

program of self-renewal with long-term hematopoietic stem cells. Proc. Natl. Acad. Sci. USA *103*, 3304–3309.

Lugli, E., Galletti, G., Boi, S.K., and Youngblood, B.A. (2020). Stem, Effector, and Hybrid States of Memory CD8$^+$ T Cells. Trends Immunol. *41*, 17–28.

Macintyre, A.N., Finlay, D., Preston, G., Sinclair, L.V., Waugh, C.M., Tamas, P., Feijoo, C., Okkenhaug, K., and Cantrell, D.A. (2011). Protein kinase B controls transcriptional programs that direct cytotoxic T cell fate but is dispensable for T cell metabolism. Immunity *34*, 224–236.

Marchingo, J.M., Sinclair, L.V., Howden, A.J., and Cantrell, D.A. (2020). Quantitative analysis of how Myc controls T cell proteomes and metabolic pathways during T cell activation. eLife *9*, e53725.

Masson, F., Minnich, M., Olshansky, M., Bilic, I., Mount, A.M., Kallies, A., Speed, T.P., Busslinger, M., Nutt, S.L., and Belz, G.T. (2013). Id2-mediated inhibition of E2A represses memory CD8+ T cell differentiation. J. Immunol. *190*, 4585–4594.

Matloubian, M., Concepcion, R.J., and Ahmed, R. (1994). CD4+ T cells are required to sustain CD8+ cytotoxic T-cell responses during chronic viral infection. J. Virol. *68*, 8056–8063.

McLane, L.M., Abdel-Hakeem, M.S., and Wherry, E.J. (2019). CD8 T Cell Exhaustion During Chronic Viral Infection and Cancer. Annu. Rev. Immunol. *37*, 457–495.

Milner, J.J., Nguyen, H., Omilusik, K., Reina-Campos, M., Tsai, M., Toma, C., Delpoux, A., Boland, B.S., Hedrick, S.M., Chang, J.T., and Goldrath, A.W. (2020). Delineation of a molecularly distinct terminally differentiated memory CD8 T cell population. Proc. Natl. Acad. Sci. USA *117*, 25667–25678.

Nie, Z., Hu, G., Wei, G., Cui, K., Yamane, A., Resch, W., Wang, R., Green, D.R., Tessarollo, L., Casellas, R., et al. (2012). c-Myc is a universal amplifier of expressed genes in lymphocytes and embryonic stem cells. Cell *151*, 68–79.

Nizet, V., and Johnson, R.S. (2009). Interdependence of hypoxic and innate immune responses. Nat. Rev. Immunol. *9*, 609–617.

Nolz, J.C., and Harty, J.T. (2011). Protective capacity of memory CD8+ T cells is dictated by antigen exposure history and nature of the infection. Immunity *34*, 781–793.

O'Sullivan, D., van der Windt, G.J., Huang, S.C., Curtis, J.D., Chang, C.H., Buck, M.D., Qiu, J., Smith, A.M., Lam, W.Y., DiPlato, L.M., et al. (2014). Memory CD8(+) T cells use cell-intrinsic lipolysis to support the metabolic programming necessary for development. Immunity *41*, 75–88.

Okkenhaug, K. (2013). Signaling by the phosphoinositide 3-kinase family in immune cells. Annu. Rev. Immunol. *31*, 675–704.

Olson, J.A., McDonald-Hyman, C., Jameson, S.C., and Hamilton, S.E. (2013). Effector-like CD8$^+$ T cells in the memory population mediate potent protective immunity. Immunity *38*, 1250–1260.

Pipkin, M.E., Sacks, J.A., Cruz-Guilloty, F., Lichtenheld, M.G., Bevan, M.J., and Rao, A. (2010). Interleukin-2 and inflammation induce distinct transcriptional programs that promote the differentiation of effector cytolytic T cells. Immunity *32*, 79–90.

Pollizzi, K.N., Patel, C.H., Sun, I.H., Oh, M.H., Waickman, A.T., Wen, J., Delgoffe, G.M., and Powell, J.D. (2015). mTORC1 and mTORC2 selectively regulate CD8$^+$ T cell differentiation. J. Clin. Invest. *125*, 2090–2108.

Preite, S., Cannons, J.L., Radtke, A.J., Vujkovic-Cvijin, I., Gomez-Rodriguez, J., Volpi, S., Huang, B., Cheng, J., Collins, N., Reilley, J., et al. (2018). Hyperactivated PI3Kδ promotes self and commensal reactivity at the expense of optimal humoral immunity. Nat. Immunol. *19*, 986–1000.

Preston, G.C., Sinclair, L.V., Kaskar, A., Hukelmann, J.L., Navarro, M.N., Ferrero, I., MacDonald, H.R., Cowling, V.H., and Cantrell, D.A. (2015). Single cell tuning of Myc expression by antigen receptor signal strength and interleukin-2 in T lymphocytes. EMBO J. *34*, 2008–2024.

Rau, A., Gallopin, M., Celeux, G., and Jaffrézic, F. (2013). Data-based filtering for replicated high-throughput transcriptome sequencing experiments. Bioinformatics *29*, 2146–2152.

Renkema, K.R., Huggins, M.A., Borges da Silva, H., Knutson, T.P., Henzler, C.M., and Hamilton, S.E. (2020). KLRG1$^+$ Memory CD8 T Cells Combine Properties of Short-Lived Effectors and Long-Lived Memory. J. Immunol. *205*, 1059–1069.

Rollings, C.M., Sinclair, L.V., Brady, H.J.M., Cantrell, D.A., and Ross, S.H. (2018). Interleukin-2 shapes the cytotoxic T cell proteome and immune environment-sensing programs. Sci. Signal. *11*, eaap8112.

Ross, S.H., and Cantrell, D.A. (2018). Signaling and Function of Interleukin-2 in T Lymphocytes. Annu. Rev. Immunol. *36*, 411–433.

Roychoudhuri, R., Clever, D., Li, P., Wakabayashi, Y., Quinn, K.M., Klebanoff, C.A., Ji, Y., Sukumar, M., Eil, R.L., Yu, Z., et al. (2016). BACH2 regulates CD8(+) T cell differentiation by controlling access of AP-1 factors to enhancers. Nat. Immunol. *17*, 851–860.

Rutigliano, J.A., Morris, M.Y., Yue, W., Keating, R., Webby, R.J., Thomas, P.G., and Doherty, P.C. (2010). Protective memory responses are modulated by priming events prior to challenge. J. Virol. *84*, 1047–1056.

Sabbagh, L., Srokowski, C.C., Pulle, G., Snell, L.M., Sedgmen, B.J., Liu, Y., Tsitsikov, E.N., and Watts, T.H. (2006). A critical role for TNF receptor-associated factor 1 and Bim down-regulation in CD8 memory T cell survival. Proc. Natl. Acad. Sci. USA *103*, 18703–18708.

Scott-Browne, J.P., López-Moyado, I.F., Trifari, S., Wong, V., Chavez, L., Rao, A., and Pereira, R.M. (2016). Dynamic Changes in Chromatin Accessibility Occur in CD8$^+$ T Cells Responding to Viral Infection. Immunity *45*, 1327–1340.

Shih, H.Y., Sciumè, G., Mikami, Y., Guo, L., Sun, H.W., Brooks, S.R., Urban, J.F., Jr., Davis, F.P., Kanno, Y., and O'Shea, J.J. (2016). Developmental Acquisition of Regulomes Underlies Innate Lymphoid Cell Functionality. Cell *165*, 1120–1133.

Shrestha, S., Yang, K., Guy, C., Vogel, P., Neale, G., and Chi, H. (2015). Treg cells require the phosphatase PTEN to restrain TH1 and TFH cell responses. Nat. Immunol. *16*, 178–187.

Sinclair, L.V., Finlay, D., Feijoo, C., Cornish, G.H., Gray, A., Ager, A., Okkenhaug, K., Hagenbeek, T.J., Spits, H., and Cantrell, D.A. (2008). Phosphatidylinositol-3-OH kinase and nutrient-sensing mTOR pathways control T lymphocyte trafficking. Nat. Immunol. *9*, 513–521.

Singh, M.D., Ni, M., Sullivan, J.M., Hamerman, J.A., and Campbell, D.J. (2018). B cell adaptor for PI3-kinase (BCAP) modulates CD8$^+$ effector and memory T cell differentiation. J. Exp. Med. *215*, 2429–2443.

Singh, K., Lin, J., Zhong, Y., Burčul, A., Mohan, P., Jiang, M., Sun, L., Yong-Gonzalez, V., Viale, A., Cross, J.R., et al. (2019). c-MYC regulates mRNA translation efficiency and start-site selection in lymphoma. J. Exp. Med. *216*, 1509–1524.

Snell, L.M., MacLeod, B.L., Law, J.C., Osokine, I., Elsaesser, H.J., Hezaveh, K., Dickson, R.J., Gavin, M.A., Guidos, C.J., McGaha, T.L., and Brooks, D.G. (2018). CD8$^+$ T Cell Priming in Established Chronic Viral Infection Preferentially Directs Differentiation of Memory-like Cells for Sustained Immunity. Immunity *49*, 678–694.e5.

Spinelli, L., Marchingo, J.M., Nomura, A., Damasio, M.P., and Cantrell, D.A. (2021). Phosphoinositide 3-Kinase p110 Delta Differentially Restrains and Directs Naïve Versus Effector CD8$^+$ T Cell Transcriptional Programs. Front. Immunol. *12*, 691997.

Stark, A.K., Chandra, A., Chakraborty, K., Alam, R., Carbonaro, V., Clark, J., Sriskantharajah, S., Bradley, G., Richter, A.G., Banham-Hall, E., et al. (2018). PI3Kδ hyper-activation promotes development of B cells that exacerbate Streptococcus pneumoniae infection in an antibody-independent manner. Nat. Commun. *9*, 3174.

Stinchcombe, J.C., and Griffiths, G.M. (2007). Secretory mechanisms in cell-mediated cytotoxicity. Annu. Rev. Cell Dev. Biol. *23*, 495–517.

Subramanian, A., Tamayo, P., Mootha, V.K., Mukherjee, S., Ebert, B.L., Gillette, M.A., Paulovich, A., Pomeroy, S.L., Golub, T.R., Lander, E.S., and Mesirov, J.P. (2005). Gene set enrichment analysis: a knowledge-based approach for interpreting genome-wide expression profiles. Proc. Natl. Acad. Sci. USA *102*, 15545–15550.

Takeda, A.J., Zhang, Y., Dornan, G.L., Siempelkamp, B.D., Jenkins, M.L., Matthews, H.F., McElwee, J.J., Bi, W., Seeborg, F.O., Su, H.C., et al. (2017). Novel PIK3CD mutations affecting N-terminal residues of p110δ cause activated

PI3Kδ syndrome (APDS) in humans. J. Allergy Clin. Immunol. *140*, 1152–1156.e10.

Tarasenko, T.N., Pacheco, S.E., Koenig, M.K., Gomez-Rodriguez, J., Kapnick, S.M., Diaz, F., Zerfas, P.M., Barca, E., Sudderth, J., DeBerardinis, R.J., et al. (2017). Cytochrome c Oxidase Activity Is a Metabolic Checkpoint that Regulates Cell Fate Decisions During T Cell Activation and Differentiation. Cell Metab. *25*, 1254–1268.e7.

Utzschneider, D.T., Charmoy, M., Chennupati, V., Pousse, L., Ferreira, D.P., Calderon-Copete, S., Danilo, M., Alfei, F., Hofmann, M., Wieland, D., et al. (2016). T Cell Factor 1-Expressing Memory-like CD8(+) T Cells Sustain the Immune Response to Chronic Viral Infections. Immunity *45*, 415–427.

van der Windt, G.J., Everts, B., Chang, C.H., Curtis, J.D., Freitas, T.C., Amiel, E., Pearce, E.J., and Pearce, E.L. (2012). Mitochondrial respiratory capacity is a critical regulator of CD8+ T cell memory development. Immunity *36*, 68–78.

Wang, R., Dillon, C.P., Shi, L.Z., Milasta, S., Carter, R., Finkelstein, D., McCormick, L.L., Fitzgerald, P., Chi, H., Munger, J., and Green, D.R. (2011). The transcription factor Myc controls metabolic reprogramming upon T lymphocyte activation. Immunity *35*, 871–882.

Wu, T., Wieland, A., Araki, K., Davis, C.W., Ye, L., Hale, J.S., and Ahmed, R. (2012). Temporal expression of microRNA cluster miR-17-92 regulates effector and memory CD8+ T-cell differentiation. Proc. Natl. Acad. Sci. USA *109*, 9965–9970.

Wu, T., Ji, Y., Moseman, E.A., Xu, H.C., Manglani, M., Kirby, M., Anderson, S.M., Handon, R., Kenyon, E., Elkahloun, A., et al. (2016). The TCF1-Bcl6 axis counteracts type I interferon to repress exhaustion and maintain T cell stemness. Sci. Immunol. *1*, eaai8593.

Xu, H., Luo, X., Qian, J., Pang, X., Song, J., Qian, G., Chen, J., and Chen, S. (2012). FastUniq: a fast de novo duplicates removal tool for paired short reads. PLoS ONE *7*, e52249.

Yang, C.Y., Best, J.A., Knell, J., Yang, E., Sheridan, A.D., Jesionek, A.K., Li, H.S., Rivera, R.R., Lind, K.C., D'Cruz, L.M., et al. (2011). The transcriptional regulators Id2 and Id3 control the formation of distinct memory CD8+ T cell subsets. Nat. Immunol. *12*, 1221–1229.

Yu, G., Wang, L.G., Han, Y., and He, Q.Y. (2012). clusterProfiler: an R package for comparing biological themes among gene clusters. OMICS *16*, 284–287.

Yukawa, M., Jagannathan, S., Vallabh, S., Kartashov, A.V., Chen, X., Weirauch, M.T., and Barski, A. (2020). AP-1 activity induced by co-stimulation is required for chromatin opening during T cell activation. J. Exp. Med. *217*, e20182009.

Zhang, X., Gan, L., Pan, H., Guo, S., He, X., Olson, S.T., Mesecar, A., Adam, S., and Unterman, T.G. (2002). Phosphorylation of serine 256 suppresses transactivation by FKHR (FOXO1) by multiple mechanisms. Direct and indirect effects on nuclear/cytoplasmic shuttling and DNA binding. J. Biol. Chem. *277*, 45276–45284.

Zhang, Y., Liu, T., Meyer, C.A., Eeckhoute, J., Johnson, D.S., Bernstein, B.E., Nusbaum, C., Myers, R.M., Brown, M., Li, W., and Liu, X.S. (2008). Model-based analysis of ChIP-Seq (MACS). Genome Biol. *9*, R137.

Zhao, F., Cannons, J.L., Dutta, M., Griffiths, G.M., and Schwartzberg, P.L. (2012). Positive and negative signaling through SLAM receptors regulate synapse organization and thresholds of cytolysis. Immunity *36*, 1003–1016.

Zhou, X., Yu, S., Zhao, D.M., Harty, J.T., Badovinac, V.P., and Xue, H.H. (2010). Differentiation and persistence of memory CD8(+) T cells depend on T cell factor 1. Immunity *33*, 229–240.

STAR★METHODS

KEY RESOURCES TABLE

REAGENT or RESOURCE	SOURCE	IDENTIFIER
Antibodies		
anti-human CD3 purified (HIT3a)	BD Biosciences	Cat #555336; RRID: AB_395742
anti-human CD28 (purified (CD28.2)	BD Biosciences	Cat #555725; RRID: AB_396068
anti-human CD62L BV 421 (DREG-56)	Biolegend	Cat #302616; RRID: AB_493043
anti-human CD4 PerCP Cy5.5 (A16A1)	Biolegend	Cat #357413; RRID: AB_2565666
anti-human CD8 APC eFluor780 (RPA-T8)	Thermo Fisher	Cat #47-0088-42; RRID: AB_1272046
anti-human CD25 Alexa 488 (BC960	Biolegend	Cat #302616; RRID: AB_493043
anti-human Granzyme B BV421 (GB11)	BD Biosciences	Cat #563389; RRID: AB_2738175
anti-human pFoxO1 (S256)	Cell Signaling Technologies	Cat #9461
anti-human FoxO1 (C29H4)	Cell Signaling Technologies	Cat #2880; RRID:AB_2106495
anti-human NFIL3 (D5K80)	Cell Signaling Technologies	Cat #14312; RRID:AB_2798446
anti-human BATF3	LS Bio	Cat #LS-C413396-100
anti-human Myc (D84C12)	Cell Signaling Technologies	Cat #5605; RRID:AB_1903938
ant-human pMyc S62 (EPR17924)	ABCAM	Cat #ab185656
anti-human GLUT1 PE (EPR3915)	ABCAM	Cat #ab209449
anti-human TCF1 (C46C7)	Cell Signaling Technologies	Cat #2206; RRID:AB_2199300
anti-mouse CD3 (2C11)	BioXCell	Cat #BE0001-1; RRID: AB_1107634
anti-mouse CD4 BV605 (RM4-5)	Biolegend	Cat #100548; RRID: AB_2563054
anti-mouse CD8 APC eFluor780 (53-6.7)	Thermo Fisher	Cat #47-0081-82; RRID: AB_1272185
anti-mouse CD8 PE (53-6.7)	Thermo Fisher	Cat #12-0081-83; RRID: AB_465530
anti-mouse CD11a FITC (M17/4)	Thermo Fisher	Cat #11-0111-85; RRID: AB_464931
anti-mouse CD25 PE-Cy7 (PC61)	Thermo Fisher	Cat #25-0251-82; RRID: AB_469608
anti-mouse CD27 FITC (LG.3A10)	Thermo Fisher	Cat #11-0271-85; RRID: AB_465002
anti-mouse CD28 purified (37.51)	BioXCell	Cat #BE0015-1; RRID: AB_1107624
anti-mouse CD28 APC (37.51)	Thermo Fisher	Cat #17-0281-82; RRID: AB_469374
anti-mouse CD44 AF700 (IM7)	Thermo Fisher	Cat #56-0441-82; RRID: AB_494011
anti-mouse CD44 FITC (IM7)	Thermo Fisher	Cat#11-0441-82; RRID: AB_465045
anti-mouse CD45.1 BV605 (A20)	Biolegend	Cat #110738; RRID: AB_2562565
anti-mouse CD45.2 PerCP(104)	Biolegend	Cat #109825; RRID: AB_893351
anti-mouse CD62L PerCPCy5.5 (MEL-14)	Thermo Fisher	Cat #45-0621-82; RRID: AB_996667
anti-mouse CD62L Alexa Fluor488 (MEL-14)	Biolegend	Cat #104420; RRID: AB_493376
anti-mouse CD62L APC (MEL-14)	Thermo Fisher	Cat #17-0621-83; RRID: AB_469411
anti-mouse CD62L eFluor 450 (MEL-14)	Thermo Fisher	Cat#48-0621-82; RRID: AB_1963590
anti-mouse CD69 PE-Cy7 (H1.2F3)	Biolegend	Cat #104512; RRID: AB_493564
anti-mouse CD71 PE (C2F2)	BD Biosciences	Cat #552367; RRID: AB_394744
anti-mouse CD98 PE-Cy7 (RL388)	Biolegend	Cat #128214; RRID: AB_2750547
anti-mouse CD127 eFluor 450 (A7R34)	Thermo Fisher	Cat #48-1271-82; RRID: AB_2016698
anti-mouse CD137 PE (1AH2)	Biolegend	Cat # 106106; RRID: AB_2287565
anti-mouse CD178 PE (MFL3)	Biolegend	Cat #106606; RRID: AB_313279
anti-mouse CD215 PerCPeFluor 710 (DNT15Ra)	Thermo Fisher	Cat #46-7149-82; RRID: AB_11150247
anti-mouse CD244 PE-Cy7 (244F4)	Thermo Fisher	Cat #25-2441-82; RRID: AB_2573432
anti-mouse IFN-γ eFluor 450 (XMG1.2)	Thermo Fisher	Cat #48-7311-82; RRID: AB_1834366
anti-mouse TNF-α PE-Cy7 (MO6-XT22BD)	BD Biosciences	Cat #557644; RRID: AB_396761

(Continued on next page)

Continued

REAGENT or RESOURCE	SOURCE	IDENTIFIER
anti-mouse IL-2 APC (JES6-5H4)	Thermo Fisher	Cat #17-7021-81; RRID: AB_469489
anti-mouse IL-2 purified (S4B6)	BioXCell	Cat #BE0043-1; RRID: AB_1107705
anti-mouse Eomes PerCP-eFluor710 (Dan11mag)	Thermo Fisher	Cat #46-4875-82; RRID:AB_10597455
anti-mouse KLRG1 PE-Cy7 (2F1)	Thermo Fisher	Cat #25-5893-82; RRID: AB_1518768
anti-mouse TCR Vα2 APC (B20.1)	Thermo Fisher	Cat #17-5812-82; RRID: AB_1659733
anti-mouse TCR Vβ5 PE (MR9-4)	BD Biosciences	Cat #553190; RRID: AB_394698
anti-mouse pSTAT5(Y694) PE (47/Stat5)	BD Biosciences	Cat #612567; RRID: AB_399858
anti-mouse/human pS6(S240/244) Alexa 647(D68F8)	Cell Signaling Technologies	Cat #5044; RRID:AB_10829359
anti-mouse/human pS6(S235/236) Alexa 488 (D57.2.2E)	Cell Signaling Technologies	Cat #4803; RRID:AB_916158
Armenian hamster Ig control	Biolegend	Cat #400902
Rat anti-mouse IgG2a control	Thermo Fisher	Cat #04-6200; RRID:AB_2532944
anti-human pFoxO1 (T24) / FoxO3a (T32)	Cell Signaling Technologies	Cat #9464; RRID:AB_329842
anti-human FoxO (C29H4)	Cell Signaling Technologies	Cat #2880; RRID:AB_2106495
anti-human Hif1α (D1S7W)	Cell Signaling Technologies	Cat #36169; RRID:AB_2799095
anti-human LAL	Thermo Fisher	Cat #PA5-27346; RRID: AB_2544822
anti-human Tip47	Thermo Fisher	Cat #PA1-46161; RRID:AB_2139115
anti-human pAKT (S473) (D9E)	Cell Signaling Technologies	Cat #4060S; RRID:AB_231504
anti-mouse AKT (C67E7)	Cell Signaling Technologies	Cat #4691S; RRID:AB_915783
anti-human p110δ (D1Q7R)	Cell Signaling Technologies	Cat #34050; RRID:AB_2799043
pZAP-70 antibody (Tyr319)/Syk (Tyr352) (65E4)	Cell Signaling Technologies	Cat #2717T; RRID:AB_2218658
ZAP70 antibody (99F2)	Cell Signaling Technologies	Cat #2705S RRID:AB_2273231
pERK antibody (Thr202/Tyr204) (D13.14.4E)	Cell Signaling Technologies	Cat #4370S; RRID:AB_2315112
ERK antibody (137F5)	Cell Signaling Technologies	Cat #4695S; RRID:AB_390779
anti-rabbit 488	Thermo Fisher	Cat #A32731; RRID: AB_2633280
anti-rabbit 647	Thermo Fisher	Cat #A21245; RRID: AB_2535813
anti-rabbit HRP	Thermo Fisher	Cat #31460; RRID: AB_228341
anti-mouse HRP	Thermo Fisher	Cat #G-21040; RRID: AB_2536527
Bacterial and virus strains		
DH5a competent cells	Thermo Fisher	Cat #18265017
Lymphocytic choriomeningitis virus Armstrong strain	Provided by McGavern Lab, NINDS, NIH	N/A
Influenza strain X31	Provided by M. Eichelberger, FDA McGuire Lab, NHGRI, NIH	N/A
Influenza strain PR8	Provided by McGuire Lab, NHGRI, NIH	N/A
Influenza strain X31-OVA	Provided by McGuire Lab, NHGRI, NIH	N/A
Influenza strain PR8-OVA	Provided by McGuire Lab, NHGRI, NIH	N/A
Biological samples		
Blood Healthy Donor	NIH Blood Bank	N/A
Peripheral blood from patients with APDS	Provided by H. Su and G. Uzel, NIAID	N/A
Chemicals, peptides, and recombinant proteins		
H-2Db LCMV $NP_{396-404}$ (FQPQNGQFI) tetramer	NIH Tetramer Facility	N/A
H-2Db LCMV GP_{33-41} (KAVYNFATM) tetramer	NIH Tetramer Facility	N/A
H-2Db Influenza $NP_{366-374}$ (ASNENMETM) tetramer	NIH Tetramer Facility	N/A

(*Continued on next page*)

Continued

REAGENT or RESOURCE	SOURCE	IDENTIFIER
H-2Db Influenza $PA_{224-233}$ (SSLENFRAYV) tetramer	NIH Tetramer Facility	N/A
$OVA_{257-264}$ (SIINFEKL)	AnaSpec	Cat #AS-60193-1
OVA T4 (SIITFEKL)	AnaSpec	Cat #AS-64403
Influenza $PA_{224-233}$	AnaSpec	Cat #AS-61636
Influenza $NP_{366-374}$	AnaSpec	Cat #AS-60624
LCMV $NP_{396-404}$ (FQPQNGQFI)	AnaSpec	Cat #AS-61700
recombinant human IL-2	NIH AIDs Reagent Program	Cat #136
recombinant mouse IL-15	NCI Repository	N/A
LIVE/DEAD^{TM} Fixable Aqua 530	Thermo Fisher	Cat #L34966
LIVE/DEAD^{TM} Fixable Near-IR	Thermo Fisher	Cat #L34976
Annexin V APC	Biolegend	Cat #640920
Annexin V binding buffer	Biolegend	Cat #422201
CAL-101	Santa Cruz Biotechnology	Cat #364453
CAL-101, GS1101	Selleckchem	Cat #S2226
zVAD FMK	R&D Systems	Cat #FK001
Akt inhibitor	Calbiochem	Cat #1240-17-1MG
Necrostatin-1	Cayman Chemical	Cat #11658
Liberase DL	Millipore	Cat #5401160001
Oligomycin	Sigma	CAS # 1404-19-9
Fluoro-carbonyl cyanide phenylhydrazone (FCCP)	Sigma	CAS # 370-86-5
Antimycin A	Sigma	CAS # 1397-94-0
Rotenone	Sigma	CAS # 83-79-4
Lymphocyte Separation Medium	MP Bio	Cat # 50494X
Golgi Stop	BD Biosciences	Cat # 554724
CellTrace Violet	Thermo Fisher	Cat #C34557
cOmplete, Mini Protease Inhibitor Cocktail	Sigma	Cat #11836153001
Sodium orthovanadate	Sigma	Cat # S6508-10G
Polybrene	Sigma	Cat #TR-1003-G
TRIzol^{TM} Reagent	Thermo Fisher	Cat #15596026
PowerUP^{TM} SYBR^{TM} Green Master Mix	Thermo Fisher	Cat# A25742
SuperScriptTM IV First-Strand Synthesis	Thermo Fisher	Cat# 18091050
**Critical commercial assays**		
Fixation/Permeabilization Solution Kit	BD Biosciences	Cat #554717
Foxp3 / Transcription Factor Staining Buffer Set	Thermo Fisher	Cat #00-5523-00
PhiPhiLux-G1D2 kit	OncoImmunin Inc	Cat # A304R1G-5
Naive CD8+ T cell isolation kit	Miltenyibiotec	Cat #130-096-543
T cell enrichment columnns	R&D Sytems	Cat #MTCC-525
PureLink Viral RNA/DNA Mini Kit	Thermo Fisher	Cat #12280050
RNeasy Plus Mini Kit	QIAGEN	Cat #74136
L-Lactate Assay kit	Cayman Chemical	Cat #700510
Seahorse XFp Cell Mito Stress Test Kit	Agilent	Cat #103010-100
Seahorse XF Glycolysis Stress Test Kit	Agilent	Cat #103020-100
**Deposited data**		
Bulk raw and processed RNA-Seq data	GEO	GEO: GSE155799
Bulk raw and processed ATAQ-Seq data	GEO	GEO: GSE155799
**Experimental models: Cell lines**		
293T	ATCC	CRL-11268

(Continued on next page)

Continued		
REAGENT or RESOURCE	SOURCE	IDENTIFIER
Experimental models: Organisms/strains		
C57BL/6	The Jackson Laboratory	Stock# 000664
C57BL/6-Tg(TcraTcrb)1100Mjb/J (OT-1)	The Jackson Laboratory	Stock# 003831
B6.Cg-Tg(Prdm1-EYFP)1Mnz/J (Blimp-YFP)	The Jackson Laboratory	Stock# 008828
B6.SJL-Ptprca Pepcb/BoyJ (CD45.1)	The Jackson Laboratory	Stock# 002014
Pi3kcd$^{E1020K/WT}$	Preite et al., 2018	N/A
Oligonucleotides		
PR8-NS F 5′ TTC ACC ATT GCC TTC TCT TC 3′	IDT	N/A
PR8-NS R 5′ CCC ATT CTC ATT ACT GCT TC 3′	IDT	N/A
PR8-NP F 5′ CAG CCT AAT CAG ACC AAA TG 3′	IDT	N/A
PR8-NP R 5′ TAC CTG CTT CTC AGT TCA AG 3′	IDT	N/A
Beta-actin F 5′ GGC TGT ATT CCC CTC CAT CG 3′	IDT	N/A
Beta-actin R 5′ CCA GTT GGT AAC AAT GCC ATG T 3′	IDT	N/A
Recombinant DNA		
Migr Foxo1	Provided by Crotty lab La Jolla Institute for Immunology	N/A
Migr Foxo1^{AAA}	Provided by Crotty lab La Jolla Institute for Immunology	N/A
Software and algorithms		
FlowJo v9.9.6	BD Bioscience	https://www.flowjo.com
Graphpad Prism version 7	GraphPad	https://www.graphpad.com
GSEA software	Subramanian et al., 2005	https://www.gsea-msigdb.org/gsea/index.jsp
Adobe Illustrator 2019	Adobe	https://www.adobe.com
MACS 1.4.2	Zhang et al., 2008	http://gensoft.pasteur.fr/docs/macs/1.4.2
FastUniq	Xu et al., 2012	https://sourceforge.net/projects/fastuniq/files/
Homer v4.10	Heinz et al., 2010	http://homer.ucsd.edu/homer/
Python 3.3.2	Python software foundation	https://www.python.org
R 3.4.0	R development core team	https://www.r-project.org
RStudio 1.0.143	RStudio Team	https://www.rstudio.com
IvG 2.3.42	The Broad Institute	http://software.broadinstitute.org/software/igv/igv2.3
CASAVA 1.8.2	Illumina	http://bioweb.pasteur.fr/packages/pack@casava@1.8.2/
TopHat 2.1.0	Anders et al., 2013	https://ccb.jhu.edu/software/tophat/index.shtml
Cufflinks 2.2.1	Anders et al., 2013	http://cole-trapnell-lab.github.io/cufflinks/
ClusterProfiler	Yu et al., 2012	https://guangchuangyu.github.io/software/clusterProfiler/
Partek Genomics Suite 6.6	Partek	https://www.partek.com/partek-genomics-suite/
Bowtie 0.12.8	Langmead et al., 2009	http://bowtie-bio.sourceforge.net/index.shtml
Other		
Seahorse Analyzer	Aligent	N/A
LSRII Analzyer	BD Biosciences	N/A
Fortessa Analyzer	BD Biosciences	N/A
Aria Cell Sorter	BD Biosciences	N/A

(*Continued on next page*)

Continued

REAGENT or RESOURCE	SOURCE	IDENTIFIER
Novex WedgeWell 10%, Tris-Glycine	Thermo Fisher	Cat #XP00100BOX
Novex WedgeWell12% Tris glycine gels	Thermo Fisher	Cat #XP00120BOX
Trans-Blot Turbo Mini 0.2 μm Nitrocellulose	BIO-RAD	Cat #1704158

RESOURCE AVAILABILITY

Lead contact

Further information and requests for resources and reagents should be directed to the lead contact, Pamela L Schwartzberg (pams@nih.gov).

Materials availability

No new unique reagents were generated in this study. Reagents will be made available upon completion of a Material Transfer Agreement.

Additional resources

The clinical trial studies are registered at ClinicalTrials.gov, Identifier: NCT00128973 and Identifier: NCT00001355.

Data and code availability

RNA-Seq and ATAC-Seq are publicly available. Raw and processed sequencing data for RNA-Seq and ATAC-Seq are available from NCBI Gene Expression Omnibus. Accession number is listed in the Key resources table.

This study does not report original code.

Any additional information required to reanalyze the data reported in this paper is available from the lead contact upon request.

EXPERIMENTAL MODELS AND SUBJECT DETAILS

Patient samples

Human subjects and guardians in this study signed written consent in accordance with Helsinki principles for enrollment in research protocols approved by NIAID Institutional review board (clinical trial registration number NCT00001355, protocol 05-I-0213 as well as clinical trial registration number NCT00128973, protocol 05-1-0213). Peripheral blood from patient A.I.1 female age 44 (Takeda et al., 2017), patient F.1 female age 9 and patient H.1 female age 16 was used for Figure 5. Peripheral blood from patient A.1 male age 12, BIII.1 female age 14, C.1 female age 15, DI.1 male age 40, D.II.2 female age 12, F.II.1 female age 17, G.1 female age 12 was used in Figure 5 (Lucas et al., 2014). Patient samples used for Figure 5 were on Sirolimus treatment. Blood from anonymous healthy donors were obtained from the NIH Clinical center under approved protocols. Peripheral blood mononuclear cells were isolated using lymphocyte separation medium gradient centrifugation.

Mice and infectious models

Commercially available mice and reagents are described in the Key resources table. Generation of *Pi3kcd*$^{E1020K/+}$ was previously described (Preite et al., 2018). For adoptive transfer experiments, *Pi3kcd*$^{E1020K/+}$ mice were bred to OT-1 as well as OT-1 BLIMP-YFP mice. We used sex and age matched 8-12 week-old mice (both male and female) for experiments and subsequent comparisons. Mice were maintained and treated under specific pathogen free (SPF) conditions in accordance with the guidelines of the NHGRI (protocol G98-3) and NINDS (protocol 1295-14) Animal Care and Use committees at the NIH (Animal Welfare Assurance #A-4149-01). For adoptive transfers 3x10^6 (day 3) or 1x10^4 (day 8) OT-1 CD8$^+$ T cells were transferred into CD45.1/CD45.2 recipients. Mice were infected with mouse adapted human influenza virus A/PR/8/34 (PR8), A/X/31 (X31), X31 containing ovalbumin X31-OVA or PR8-OVA. Mice were exposed to aerosolized (Glas-Col) 500 TCID in 10ml of saline. Influenza A/PR8/34 (H1N1) shares the immuno-dominant nucleoprotein (NP) epitope of the HK-X31 strain but differs in the major neutralizing antibody epitopes of the hemagglutinin (H) and neuraminidase (N) proteins (Sabbagh et al., 2006). Expression of virus in the lungs of infected mice was determined by qPCR (Tarasenko et al., 2017). Mice were injected with 2x10^5 PFU of LCMV Armstrong strain (Wu et al., 2016). Viral titers in the liver were determined by plaque assay using Vero cells (Wu et al., 2016).

METHOD DETAILS

Bone marrow chimeras

8 week old WT CD45.1$^+$ CD45.2$^+$ recipients were sub-lethally irradiated (900 rads) using a Cesium source 18hr before retro-orbital transfer of 5x10^6 BM cells from 8 week old donors. For mixed BM chimeras, WT CD45.1$^+$ and *Pi3kcd*$^{E1020K/+}$ CD45.2$^+$ (ratio of 4:1) was transferred. Mice were maintained on acid water for 5-6 weeks and analyzed after 8-12 weeks.

Cell Culture

OT-1 (2.5x10^6) splenocytes were stimulated with $OVA_{257-264}$ or T4 peptide for 3 days in complete media (Kapnick et al., 2017). On day 3, cells were washed, counted and 10 IU/ml of recombinant IL-2 or 10ng/ml IL-15 was added to cultures (2x10^5 cells/ml) (O'Sullivan et al., 2014). Cells were washed, counted, and fresh cytokines added every 24hr. For IL-2 blocking, anti-mouse IL-2 (20μg/ml) or Ig control (20μg/ml) was added to the cultures. For assays including inhibitors: Idelalisib p110δ inhibitor (CAL-101): 2nM, Z-VAD-FKM caspase inhibitor: (100μM), AKTi: 50-200nM, Necrostatin-1: 0.3mM or DMSO vehicle control was added to cultures. Target and flow-based cytotoxicity assays were previously described (Kapnick et al., 2017). B cells were stimulated with LPS as previously described (Zhao et al., 2012).

Flow Cytometry

Antibodies and reagents are described in Key resources table. For staining, single cell suspension were made from spleen or lymph nodes in RPMI (Penn/Strep, L-Glut, 2ME and 10% FBS). After ACK (ammonium chloride) lysis of RBCs, cells were washed once, counted, and resuspended in FACS buffer (PBS, 2% FBS). Cells were incubated with antibodies for 30-45 min on ice. For Intracellular cytokine staining, cells were permeabilized with BD Cytofix/Cytoperm. Intracellular staining of TFs cells were permeabilized using Foxp3 staining buffer kit. Unconjugated antibodies were detected with secondary antibodies conjugated with 488 or 647. For intracellular staining of cytoplasmic phospho-proteins, cells were fixed with 4% PFA, permeabilized with cold methanol at −20°C as described (Gomez-Rodriguez et al., 2016). For short-term cytokine stimulations, cells were incubated in serum free media for 3hr prior to the cytokine addition. The following reagents were used according to manufacture instructions: PhiPhiLux-G1D2 kit, Annexin-V-APC, Live/Dead Fixable Aqua Dead Cell Stain Kit. MHC class I tetramers specific for LCMV and influenza CD8 epitopes were obtained from the NIAID tetramer facility (Emory University, Atlanta, GA). The following gates were applied before identification of specific cell types: FSC-A/SSC-A, exclusion of doublets (FSC-H/FSC-W and SSC-H/SSC-W) live cells (negative for Aqua). Flow cytometry based conjugate assay was performed as previously described (Cannons et al., 2010). Flow cytometry was performed on a LSRII (BD Bioscience) and data analyzed with FlowJo 9.9 software (Treestar).

Western Blots

Stimulations and immunoblot analysis was performed as previously described (Cannons et al., 2004). Briefly, T cells were stimulated with plate bound anti-CD3 (5 μg/ml) +/− anti-CD28 (5 μg/ml) for the indicated times. For short term stimulations, T cells were resuspended in serum free media at 10^8/ml and 100μl of T cells were added to a coated 6 well dish. at 37°C At the indicated times, 100μl of 1% SDS in PBS (plus protease inhibitor minitab (Sigma) and sodium orthovanadate) was added to each well followed by the addition of 900ul 1% TritonX in PBS (containing inhibitors). Lysates were sheared through a 25-gauge needle with a 1 cc syringe 5 times. Lysates were spun at 14 krpm for 15 min at 4°C. For longer time courses, 200-400μl T cells (10^7) were stimulated in media in 2% serum at 37°C in a 24 well dish, prior to removal of media and lysis as above. For assays when cells were cultured in cytokines, 10^7 cells were transferred to an Eppendorf tube and spun down, prior to lysis as above. Proteins (in reducing sample buffer) were separated by SDS-PAGE and transferred to nitrocellulose. Membranes blocked with TBS plus 5% BSA, 0.1% Tween-20 were incubated with primary antibodies overnight at 4°C followed by incubation with HRP-conjugated secondary antibodies for 60 minutes. Signals were detected by chemiluminescence.

Metabolic Assays

L-Lactate levels were measured in the cell supernatants using a glycolysis cell-based assay kit (Cayman Chemicals). Oxygen consumption rates (OCR) and extracellular acidification rates (ECAR) were measured with the Seahorse XFe96 Analyzer (Agilent). Briefly, a fixed number of sorted $CD8^+$ T cells were adhered to XF96 cell culture microplates immediately before assays using Cell-Tak (Corning). Mito Stress tests were performed in response to 1μM oligomycin, 1.5μM fluoro-carbonyl cyanide phenylhydrazone (FCCP), and 1μM antimycin A plus 0.5μM rotenone in XF media (non-buffered RPMI 1640, 25 mM glucose, 2mM L-glutamine, 1mM sodium pyruvate). Basal respiration: (last rate measurement before oligomycin injection - non-mitochondrial respiration rate), Maximal respiration: (maximal rate after FCCP injection - non-mitochondrial respiration rate), ATP production: (last rate measurement before oligomycin injection – minimal rate measurement after oligomycin injection), Spare respiratory capacity as %: (Maximal respiration / Basal respiration) x 100. Glyco Stress tests were performed in response to 25mM glucose, 1μM oligomycin, and 50mM 2-deoxyglucose (2-DG, Sigma) in XF media (non-buffered RPMI 1640 and 2mM L-glutamine) (van der Windt et al., 2012). Glycolysis: (maximal rate measurement before oligomycin injection – last rate measurement before glucose injection), Glycolytic Capacity: (maximal rate measurement after oligomycin injection – last rate measurement before glucose injection), Glycolytic reserve: (Glycolytic capacity - Glycolysis) (van der Windt et al., 2012). Calculations based on Agilent's Report Generator Guide.

Retroviral transduction

Migr, Migr-Foxo1 and Migr-$Foxo1^{AAA}$ were kindly provided by Y.S. Choi, J. Choi and S. Crotty. Retroviral stocks were generated as previously described (Cannons et al., 2004). For retroviral transduction, OT-1 $CD8^+$ T cells were activated with $OVA_{257-264}$ for 24hr. Cells were transduced with viral supernatants plus polybrene (8μg/ml) by centrifuging at 2500 rpm for 90 mins at 30°C. Cells were subsequently cultured in fresh media. Expression of cell surface markers were evaluated 24hr post-transduction.

RNA-Sequencing and analysis

Viable, CD62L$^+$CD44^{lo} CD8$^+$ T cells were sorted from spleens of WT OT-I or *Pik3cd*$^{E1020K/+}$ OT-1mice (> 99% purity). These were lysed and stored in Trizol reagent either directly *ex vivo* or following *in vitro* stimulation. For the latter, blasting, live, CD8$^+$ T cells were sorted after 72hr of antigen exposure (day 3) or upon subsequent culture with IL-2 or IL-15 for 24 or 48hr. 2-3 biological replicates were sequenced per genotype/condition and equal numbers were collected per replicate (40x10^4 cells). Total RNA was isolated by phenol-chloroform extraction with GlycoBlue as co-precipitant (7-15 μg per sample; Life Technologies) and poly(A)$^+$ mRNA enriched by oligo-dT-based magnetic separation. Single-end read libraries were prepared using the NEBNext Ultra RNA Library Prep Kit and sequenced with a HiSeq 2500 instrument (Illumina, San Diego, CA).

50 bp reads (> 20x10^6 per sample) were aligned onto mouse genome build mm9 with TopHat, assembled with Cufflinks and gene-level counts compiled with htseq-count (Anders et al., 2013). Counts were then normalized, differentially expressed genes (DEG) called, and TPM values calculated using edgeR (Anders et al., 2013). To minimize normalization artifacts, transcripts failing to reach an empirically defined count threshold were purged using HTSFilter (Rau et al., 2013). DEG classification indicates > 2 fold pairwise change and Benjamini-Hochberg (BH) adjusted p value < 0.05 (Figure 4A). An offset value was added to all TPM (TPM+1) and those failing to reach a value > 2 TPM in any genotype/condition were excluded, as were micro-RNAs, sno-RNAs and sca-RNAs. prcomp was used for PCA with TPM+1 values as input. hclust was used for euclidian clustering with the following TPM+1 sets as inputs: (1) genes defined by Gene Set Enrichment Analysis (GSEA) as core enriched elements within the IL-2-STAT5 signaling pathway (Molecular Signatures database hallmark gene sets) (Figure 5B) (Liberzon et al., 2015), and (2) all DEGs called across pairwise comparisons (Figure 6D).

Clusterprofiler was used to mine the KEGG and Molecular Signatures (MSigDB) gene ontology databases (Yu et al., 2012). For GSEA, input genes were ranked based a composite metric (Log2FC x -log10 pVal) and tested against MSigDB hallmark (Figures 4B, 4C, 5A, and 5B) (Liberzon et al., 2015). For Hypergeometric Testing, input gene sets were defined by: (1) splitting DEGs into negatively (*Pik3cd*$^{E1020K/+}$ < WT) and positively (*Pik3cd*$^{E1020K/+}$ > WT) regulated fractions (Figure 4E), or (2) hierarchical clustering of compiled DEGs (Figures 6D and 6E), then tested against KEGG, MSigDB TF targets (C3_TFT) and/or MSigDB immunologic signatures (C7) (Liberzon et al., 2015).

GSEA plots were drawn with enrichplot, heatmaps with heatmap and all other plots with ggplot2 or DataGraph (Visual Data Tools Inc). All input data and test results available in Tables S1 and S2. Raw and processed sequencing data are available from the NCBI Gene Expression Omnibus under accession number GSE155799.

ATAC-Sequencing and analysis

ATAC-Seq was performed according to a modified published protocol (Corces et al., 2016). Ten thousand cells, isolated as above, were pelleted and washed with 50 μL PBS. After pelleting the nuclei by centrifuging at 500 x g for 10 min at 4°C, the pellets were resuspended in 50 μL transposase mixture including 25 μL of 2xTD buffer (Tagment DNA buffer, 15027866, Illumina), 2.5 μL of TDE1 (Tagment DNA enzyme, 15027865, Illumina), 0.5 μL of 1% digitonin (G9441, Promega), 22 μL of nuclease-free water. The reaction was incubated at 37°C with shaking at 300 rpm for 30 min. The fragmentalized DNAs were then purified using a QIAGEN MinElute kit (28006) in 10ul elution buffer. Transposed fragments were amplified with 10 or 11 cycles of PCR based on the amplification curve using primers described previously (Buenrostro et al., 2013). Once the libraries were purified using a QIAGEN PCR cleanup kit (28106), they were further sequenced for 50 cycles (paired-end reads) on a HiSeq 2500.The following software were used: Bowtie 0.12.8 (Langmead et al., 2009), Homer v4.10 (Heinz et al., 2010), MACS 1.4.2, Python 3.3.2 (https://www.python.org),R 3.4.0 (https://www.R-project.org), RStudio 1.0.143 (https://www.rstudio.com/), Igv 2.3.42. ATAC-Seq data were processed as previously described with minor modifications (Shih et al., 2016). Raw sequencing data were processed with CASAVA 1.8.2 to generate FastQ files. ATAC-Seq reads from two biological replicates for each sample were mapped to the mouse genome (mm9 assembly) using Bowtie 0.12.8. In all cases, redundant reads were removed using FastUniq (Xu et al., 2012). Regions of open chromatin were identified by MACS (version 1.4.2) (Zhang et al., 2008) using a P value threshold of $1x10^{-5}$. Only one mapped read to each unique region of the genome that was less than 175 base pairs was kept and used in peak calling. Peak intensities (‘tags’ column) were normalized as tags-per-10-million reads (RP10M) in the original library and were plotted in R using Rstudio. Differentially accessible genomic regions were determined by merging MACS called ATAC-Seq peaks from different conditions using the mergePeaks module in HOMER. Enrichment of TF motifs among genomic regions with differential chromatin accessibility were analyzed by the findMotifsGenome module in HOMER. BigWig tracks were generated by HOMER and visualized by Igv. ATAC-Seq datasets for naive and memory CD8$^+$ T cell were downloaded from previous GEO database accession number GEO: GSE77695 (Shih et al., 2016). Cells used for ATAC-Seq were a portion of the cells isolated for RNA-Seq above.

Data Analysis

Flow cytometry was performed using LSRII and sort purification was performed on a BD FACS Aria Fusion. All data were analyzed using FlowJo (9). Extracellular Flux analysis were done with Agilent Seahorse XFe96 Analyzer.

QUANTIFICATION AND STATISTICAL ANALYSIS.

Data were analyzed via Prism 6 (GraphPad Software) using non-parametric unpaired Mann-Whitney U test for comparison of two unpaired groups. Graphs show mean ± SEM. *p < 0.05, **p < 0.01 or as indicated in the figures.

Cell Reports, Volume *37*

Supplemental information

PI3Kδ coordinates transcriptional, chromatin, and metabolic changes to promote effector CD8$^+$ T cells at the expense of central memory

Jennifer L. Cannons, Alejandro V. Villarino, Senta M. Kapnick, Silvia Preite, Han-Yu Shih, Julio Gomez-Rodriguez, Zenia Kaul, Hirofumi Shibata, Julie M. Reilley, Bonnie Huang, Robin Handon, Ian T. McBain, Selamawit Gossa, Tuoqi Wu, Helen C. Su, Dorian B. McGavern, John J. O'Shea, Peter J. McGuire, Gulbu Uzel, and Pamela L. Schwartzberg

Supplemental Figure 1

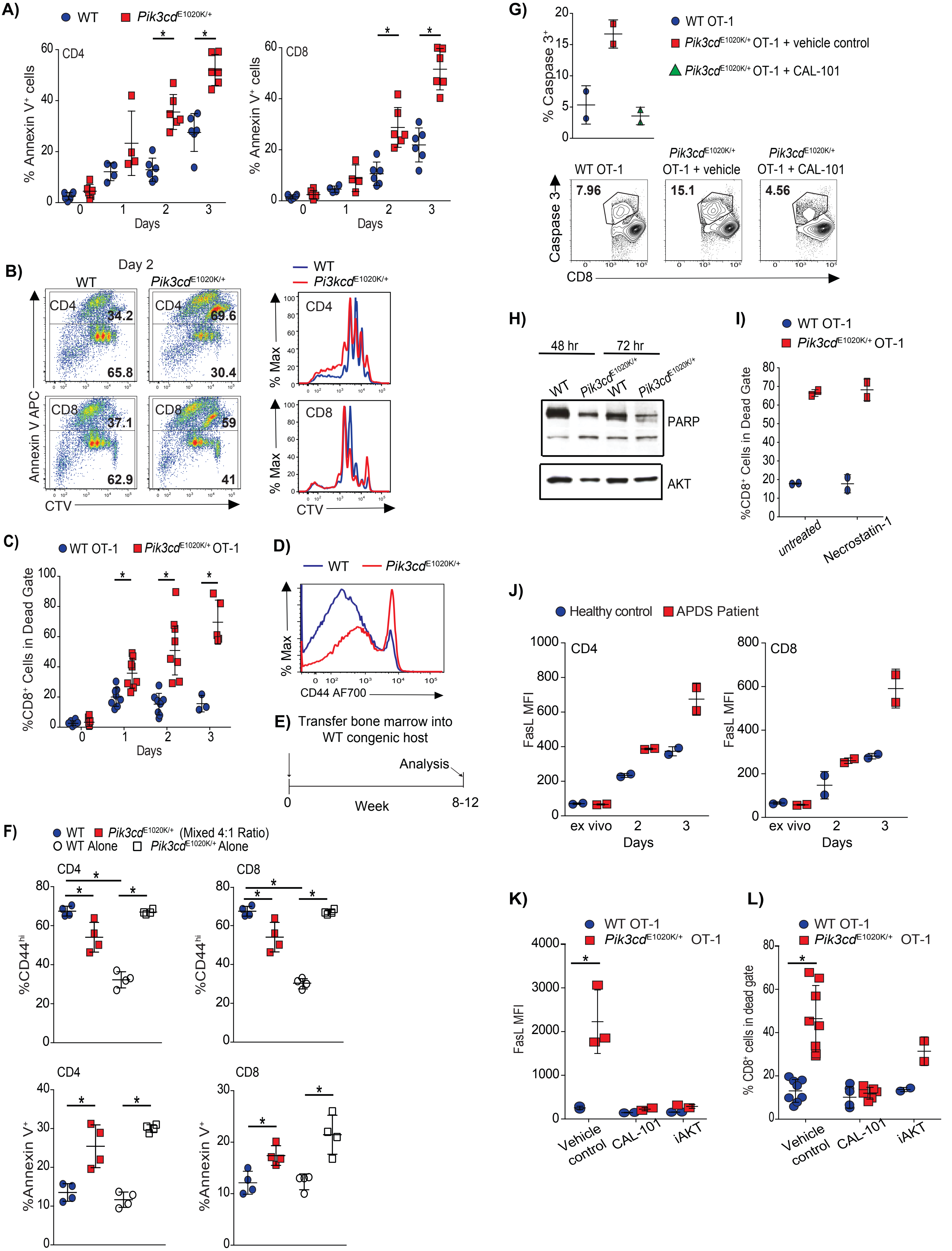

Supplemental Figures

Figure S1. *Pik3cd*$^{\mathbf{E1020K/+}}$ T cells reveal pronounced FasL-mediated cell death. Related to Figure 1.

(A-B) CTV labeled WT and *Pik3cd*$^{E1020K/+}$ CD4$^+$ (left panel) and CD8$^+$ (right panel) T cells stimulated with anti-CD3 plus anti-CD28 and evaluated for A) Annexin-V staining, B) representative flow plot (left panel), histogram of viable cells (right panel) (n=6). (C) Peptide stimulated OT-1 cells were evaluated for cell death by viability stain (n=3-6). (D) Flow cytometric analysis: CD44 expression on viable CD8$^+$ T cells from WT and *Pik3cd*$^{E1020K/+}$ mice (mice aged 10 weeks). (E-F) Recipients received WT or *Pik3cd*$^{E1020K/+}$ bone marrow, or for mixed chimeras, recipients received bone marrow from WT and *Pik3cd*$^{E1020K/+}$ (4:1 ratio). E) Experimental outline. F) Percentage of CD44^{hi} CD4$^+$ or CD8$^+$ T cells (top panels), percentage of Annexin-V$^+$ CD4$^+$ or CD8$^+$ T cells (lower panels). (G) OT-1 cells stimulated with peptide +/- of p110δ inhibitor, CAL-101, and evaluated for caspase-3 activity, 48hr. Bottom panel: representative flow plot, n=2. (H) WT and *Pik3cd*$^{E1020K/+}$ T cells stimulated with anti-CD3 plus anti-CD28. Representative western blot for PARP and AKT (n=2). (I) Cell death analysis of OT-1 cells stimulated with peptide +/- Necrostatin-1 (n=2). (J) Surface FasL MFI: *ex vivo* and anti-CD3 plus anti-CD28 stimulated CD4$^+$ (left panel) and CD8$^+$ (right panel) T cells from healthy controls and ADPS patients (n=2). (K-L) OT-1 cells evaluated post peptide stimulation in the presence of p110δ inhibitor CAL-101 or iAKT (n=2-5). K) FasL MFI from viable cells and L) Cell death analysis, (n=2-3). Graphs show mean ± SEM *$p<0.05$.

Supplemental Figure 2

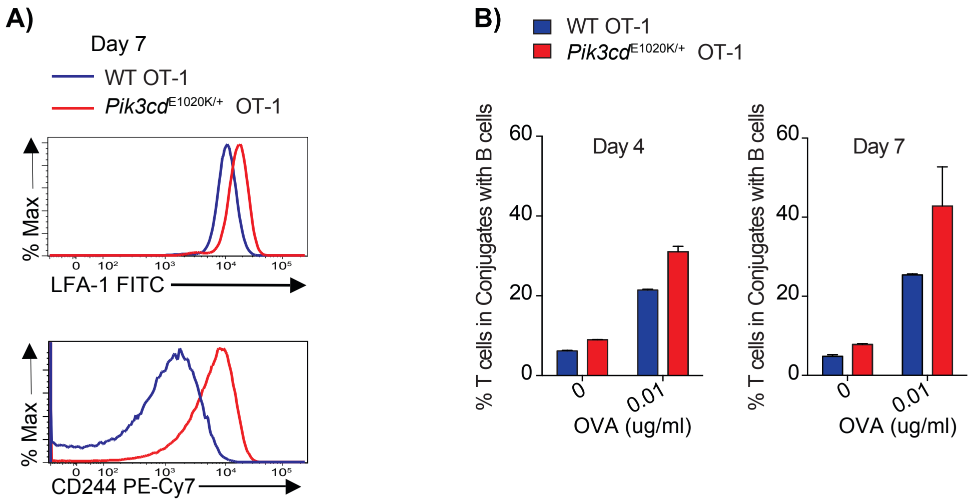

Figure S2. *Pik3cd*$^{E1020K/+}$ OT-1 CD8^{+} T cells display an early and enhanced effector phenotype.

Related to Figure 2.

(A) OT-1 T cells were stimulated with peptide for 3 days and expanded in exogenous IL-2. Flow cytometric analysis of LFA-1 (top panel) and CD244 (2B4) (bottom panel) expression on day 7 (n=3, representative histogram shown). (B) WT and *Pik3cd*$^{E1020K/+}$ OT-1 cells were stimulated with peptide for 3 days and expanded in exogenous IL-2 for the indicated times. Conjugate efficiency with LPS-activated B cells pulsed with $OVA_{257-264}$ (n=2). Graphs show mean ± SEM $p<0.05$.

Supplemental Figure 3

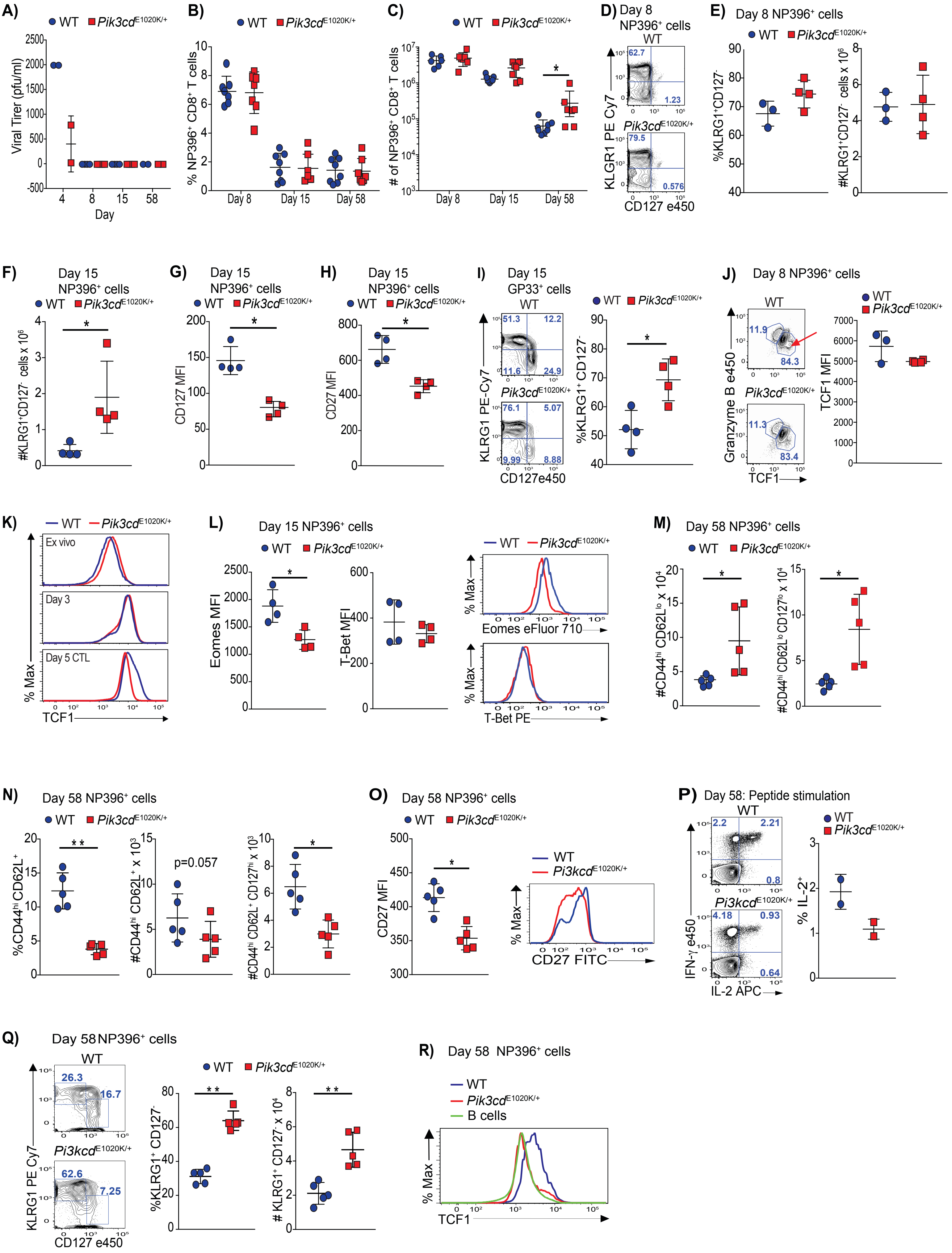

Figure S3. *Pik3cd*$^{E1020K/+}$ mice exhibit impaired development of T_{CM} populations. Related to Figure 3.

Mice were infected with LCMV Armstrong strain (n=2, 2-5 mice/group/time point). (A) Viral plaque assay from liver samples. B) Percentage and C) number of NP396-specific $CD8^+$ T cells on day 8, 15 and 58 post-infection. (D-E) Day 8 NP396-specific $CD8^+$ T cells. D) Analysis of KLGR1 and CD127 expression, representative flow plot, E) %$KLRG1^+CD127^-$ cells (left panel), number of $KLRG1^+CD127^-$ cells (right panel). (F-H) Day 15 NP396-specific $CD8^+$ T cells, F) number of $KLRG1^+CD127^-$ cells. G) CD127 MFI and H) CD27 MFI. (I) Day 15 GP33-specific $CD8^+$ T cells. Analysis of KLGR1 and CD127 expression, representative flow plot (left panel). %$KLRG1^+CD127^-$ cells (right panel). (J) Day 8 NP396-specific $CD8^+$ T cells. Analysis of Granzyme B and TCF1 expression, representative flow plot, left panel. Arrow points to $TCF1^{hi}$ population. TCF1 MFI, right panel. (K) OT-1 T cells were stimulated with peptide for 3 days and subsequently cultured with IL-2. Viable OT-1 cells were examined for TCF1 expression (n=3, representative histograms). (L) Day 15 NP396-specific $CD8^+$ T cells, Eomes MFI (left panel), T-Bet MFI (middle panel)), representative histograms, right panel. (M-N) Day 58 NP396-specific $CD8^+$ T cells. Cell numbers of $CD44^{hi}CD62L^{lo}$ (total T_{EM}) and $CD44^{hi}CD62L^{lo}CD127^{lo}$ (LLEC), populations; percentages and cell numbers of $CD44^{hi}CD62L^+$ and cell numbers of $CD44^{hi}CD62L^+CD127^{hi}$ T_{CM} cells. (O) Day 58 NP396-specific $CD8^+$ T cells, CD27 MFI (left panel), representative histogram (right panel). (P) Day 58, splenocytes stimulated with peptide and evaluated for IFN-γ and IL-2 production. Left panel, flow plot and right panel, % $IL\text{-}2^+$ cells. (Q) Day 58 NP396-specific $CD8^+$ T cells evaluated for KLRG1 and CD127 (left panel, representative flow plot), right panels: percentage and cells numbers of $KLRG1^+CD127^-$ populations. (R) Day 58 NP396-specific $CD8^+$ T cells, representative TCF1 histogram. Graphs show mean ± SEM *$p<0.05$, **$p<0.01$.

Supplemental Figure 4

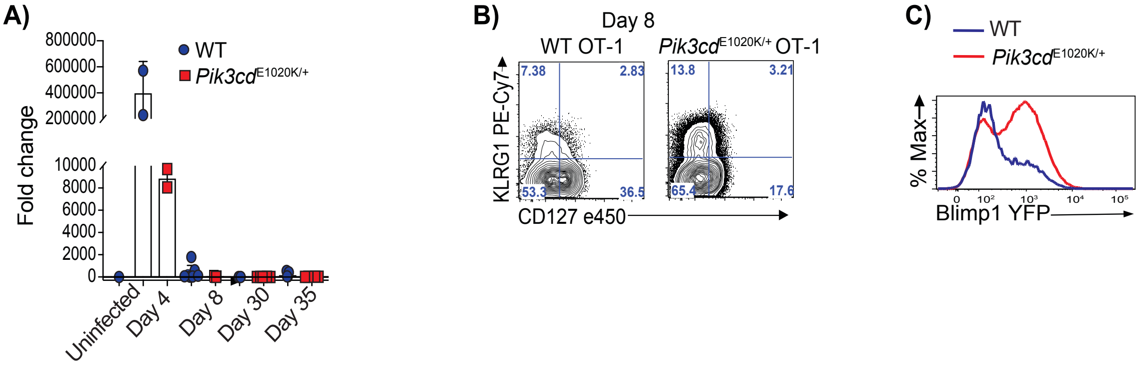

Figure S4. ***Pik3cd*$^{\mathbf{E1020K/+}}$ mice exhibit altered responses to influenza infection. Related to Figure 3.**

(A) Lung influenza viral titer following X31 infection and PR8 challenge (n=2, 3-5 mice per time point). (B) WT and *Pik3cd*$^{E1020K/+}$ OT-1 cells were transferred into congenic hosts, subsequently infected with influenza X31-OVA (n=2, 3 mice/genotype/time point). Analysis of CD127 and KLRG1 expression on transferred OT-1 cells, day 8 (representative flow plot). (C) OT-1 Blimp1-YFP $CD8^+$ T cells were transferred into CD45.1/2 host, subsequently infected with aerosolized X31-OVA influenza. Blimp1-YPF expression assessed day 8 post-infection (n=3 mice, representative example). Graphs show mean ± SEM.

Supplemental Figure 5

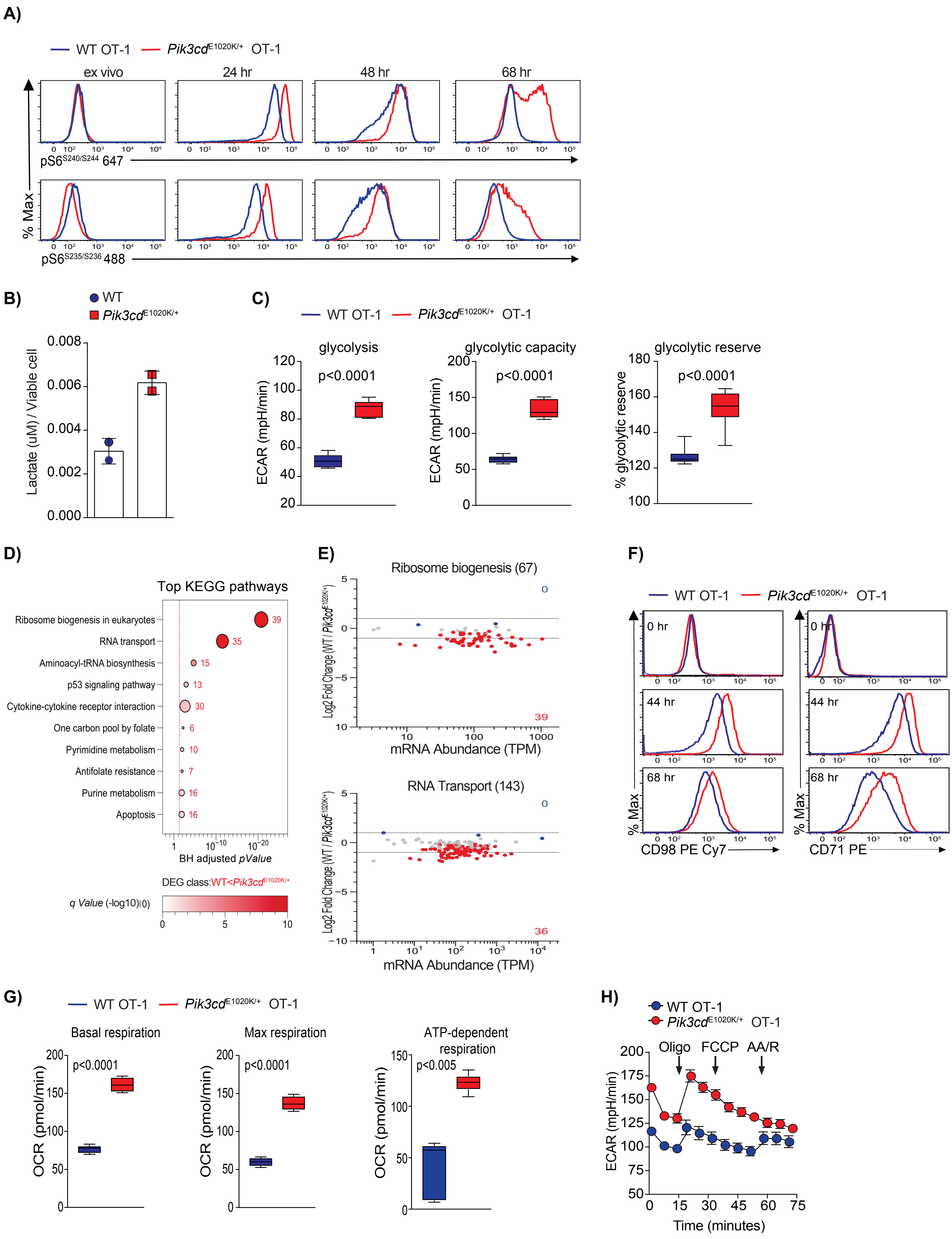

Figure S5. Activated *Pik3cd*$^{E1020K/+}$ OT-1 T cells demonstrate elevated and sustained pS6 and c-Myc expression accompanied by metabolic perturbations.

Related to Figure 4.

(A) Flow cytometric analysis of pS6$^{S235/236}$ and pS6$^{S240/244}$ from viable WT and *Pik3cd*$^{E1020K/+}$ OT-1 cells stimulated with peptide for the indicated times (n=3, representative histogram shown). (B) L-lactate was measured in CD8^{+} T cell supernatants by ELISA, 24 hr following anti-CD3 plus anti-CD28 stimulation (n=2). (C) WT and *Pik3cd*$^{E1020K/+}$ OT-1 CD8^{+} T cells were stimulated with peptide for 3 days. Sorted viable CD8^{+} T cells were evaluated via extracellular flux: ECAR was measured under basal conditions and in response to exogenous glucose, oligomycin and 2-deoxy-D-glucose. Glycolysis, glycolytic capacity and reserve are shown (n=3, representative example shown). (D-E) RNA-Seq analysis of gene expression. D) Scatter plot shows HGT data for top 10 enriched KEGG pathways among positively regulated DEGs (*Pik3cd*$^{E1020K/+}$>WT). Size and color saturations are proportional to gene count (total shown) and *p* value, respectively, n=3 biological replicates. (E) M (log ratio) and A (mean average) data (MA plots) show TPM and log2 fold change for all genes within selected KEGG pathways. Negatively (*Pik3cd*$^{E1020K/+}$<WT) and positively (*Pik3cd*$^{E1020K/+}$>WT) regulated DEGs are highlighted in blue and red, respectively. (F) Flow cytometric analysis of peptide stimulated OT-1 cells: CD98 (left panel) and CD71 (right panel), (n=3, representative example). (G-H) OT-1 T cells stimulated with peptide for 3 days. Extracellular flux analysis on sorted viable CD8^{+} T cells: G) OCR, basal respiration, maximal respiration and ATP-dependent respiration calculated as in methods and H) ECAR in response to oligomycin, FCCP, and antimycin A plus rotenone in the presence of glucose (n=3, representative example). Graph B show mean ± SEM. Graphs C, G and H show mean ± SEM *p value in graph.

Supplemental Figure 6

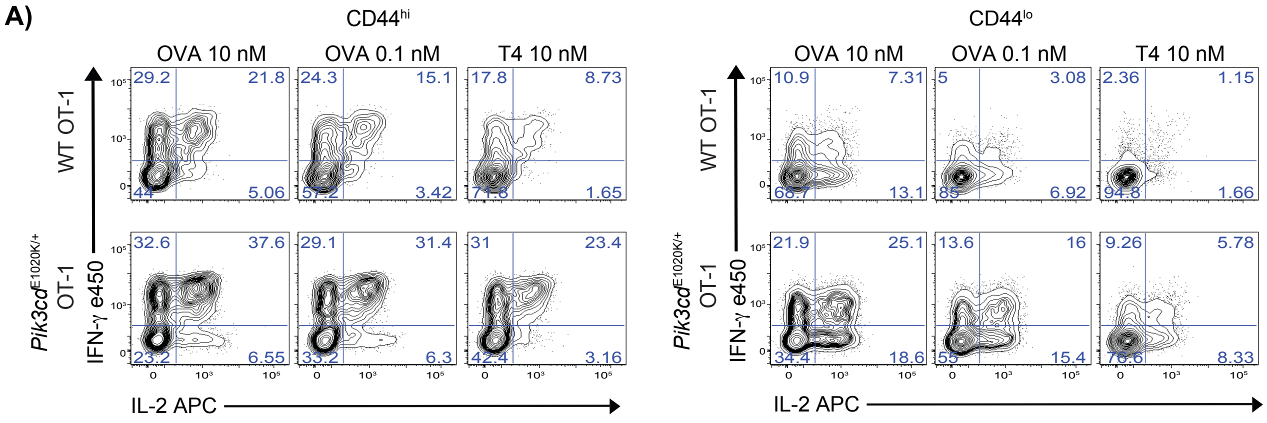

Figure S6. ***Pik3cd*$^{\mathbf{E1020K/+}}$ OT-1 CD8^{+} T cells produce early and elevated IL-2.**

Related to Figure 5.

(A) OT-1 T cells were stimulated with peptide and Golgi Stop for 3hr. Viable CD8^{+} T cells gated on CD44^{hi} (left panels) or CD44^{lo} (right panels) expression were assessed for IFN-γ and IL-2 production. Representative flow plots shown, (n=3).

Supplemental Figure 7

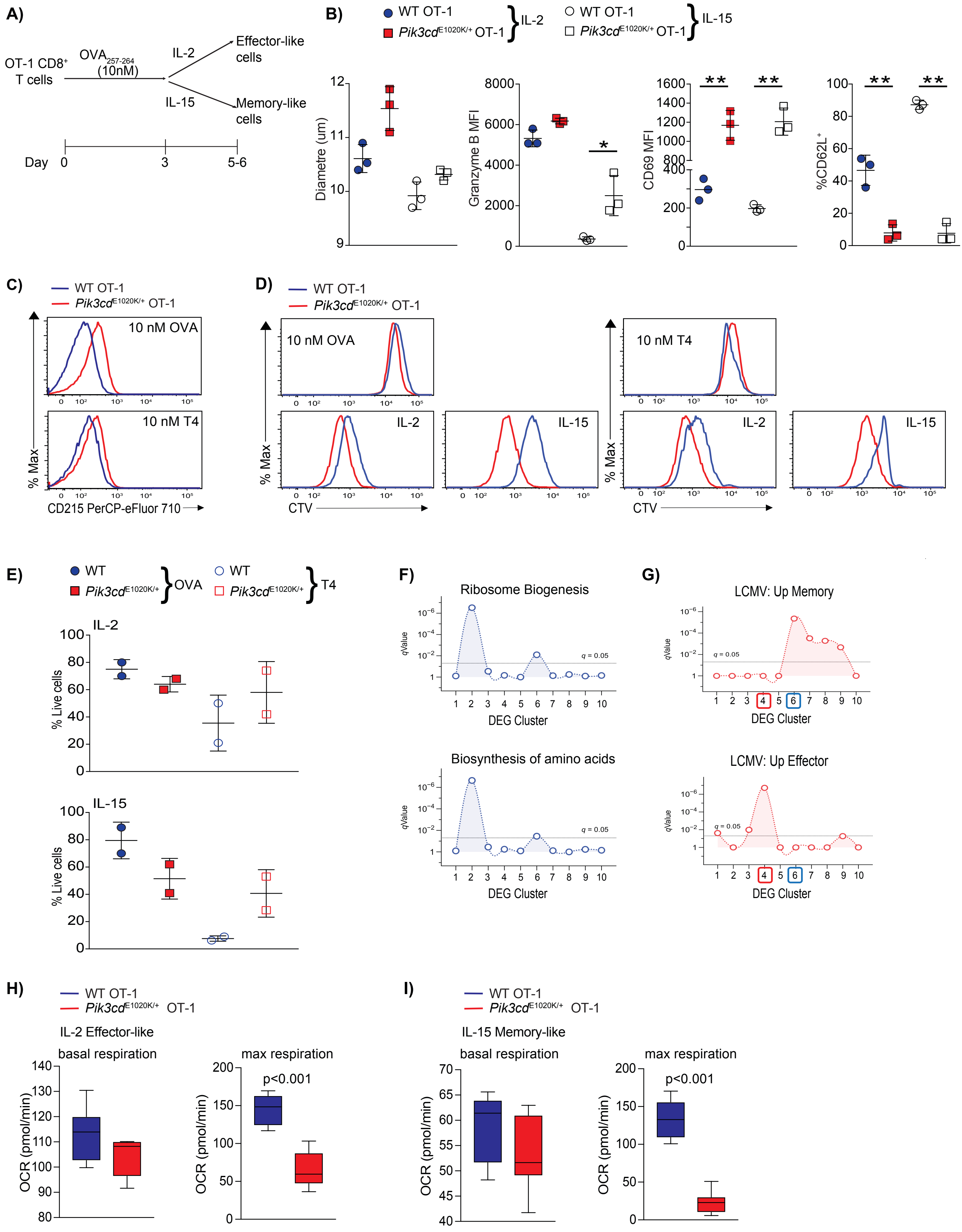

Figure S7. IL-15 differentiated *Pik3cd*$^{E1020K/+}$ OT-1 CD8^{+} T cells display features exemplified by lytic effector cells.

Related to Figure 6.

(A-B) OT-1 cells were stimulated with peptide for 3 days and subsequently cultured with cytokine to generate IL-2-effectors or IL-15-memory cells. A) Experimental outline. B) Cell diameter and flow cytometric analysis evaluating Granzyme B, CD69 and CD62L expression (n=3, summary plots shown). (C) OT-1 cells were activated with peptide for 3 days and assessed for CD215 expression, (n=3, representative histogram shown). (D-E) OT-1 cells were activated with $OVA_{257-264}$ or T4 for 3 days. Viable cells were CTV labeled and cultured in either IL-2 or IL-15 for 2 days. D) Cell expansion evaluated by CTV dilution. E) Cell viability evaluated post-cytokine culture. (F-G) RNA-Seq analysis of gene expression at day 6 on sorted viable OT-1 cells. Line graphs show enrichment of Kyoto Encyclopedia of Genes and Genomes (KEGG) pathways (F) or curated effector/memory CD8^{+} T cell gene sets (G) across 10 row clusters from (Figure 6C) https://www.gsea-msigdb.org/gsea/msigdb/cards/KAECH_DAY15_EFF_VS_MEMORY_CD8_TCELL_DN.html. (H-I) OT-1 cells were peptide stimulated for 3 days and subsequently cultured with either H) IL-2 or I) IL-15 to generate effector-like and memory-like cells, respectively. Extracellular flux analysis: OCR measured in real time under basal conditions and in response to mitochondrial inhibitors oligomycin, FCCP and antimycin A plus rotenone. OCR: basal and maximal respiration assessed (representative data shown, n=4). Graphs B and E show mean ± SEM *p<0.05, **p<0.01.Graphs H and I show mean ± SEM *p value in graph.

Supplemental Figure 8

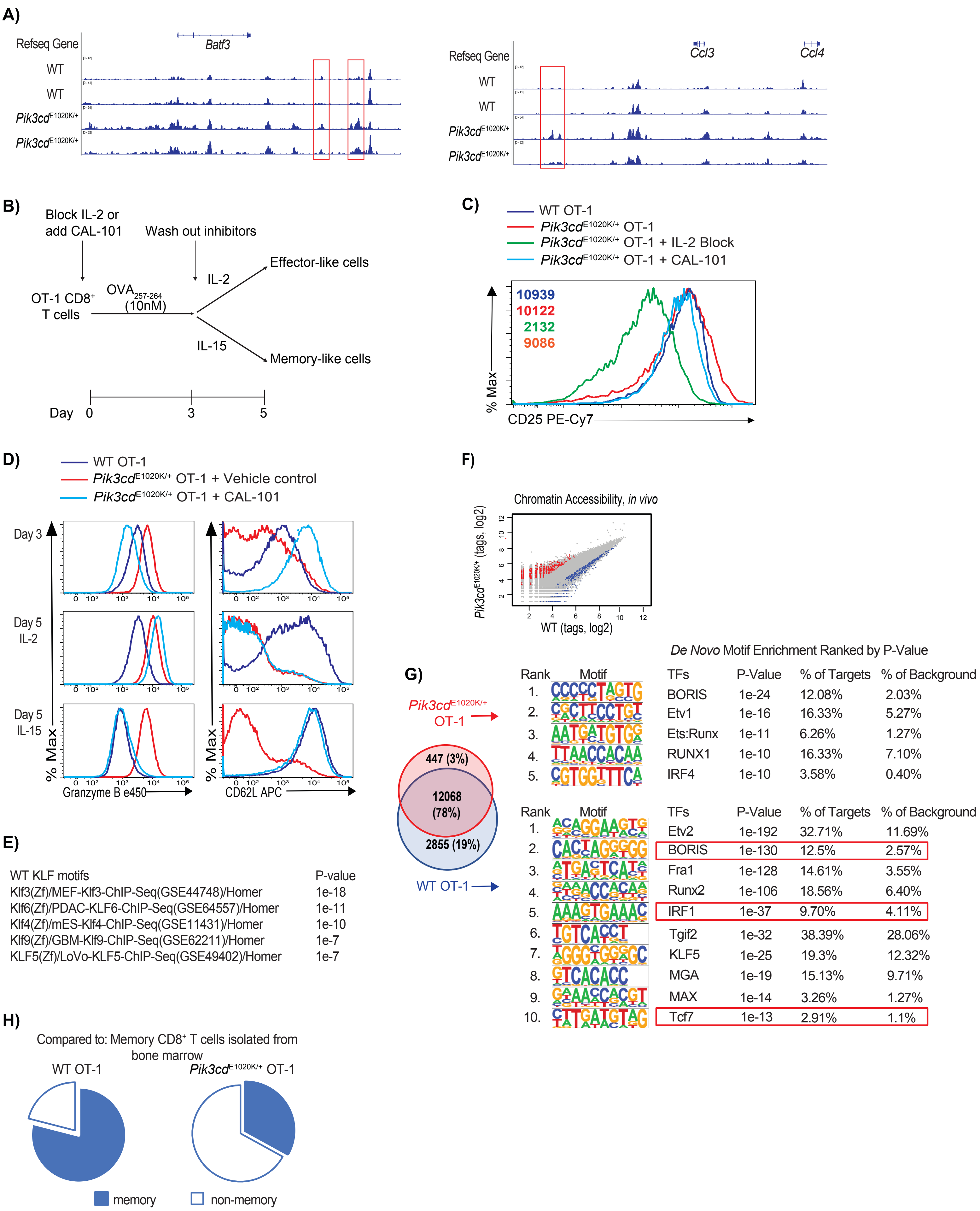

Figure S8. **Effector differentiation of *Pik3cd*$^{E1020K/+}$ CD8^{+} T cells is determined by IL-2. Related to Figure 7.**

(A) Genomic tracks of ATAC-Seq signal profile across *Batf3* (left panel) and *Ccl3* (right panel). Red boxes highlight differences in chromatin accessibility. (B-D) OT-1 cells were stimulated with peptide for 3 days +/- blocking IL-2 antibodies, p110δ inhibitor CAL-101, Ig control or vehicle control and subsequently cultured with either IL-2 or IL-15 to generate effector-like or memory-like cells, respectively (n=3). B) Experimental outline. C) Flow cytometry analysis of CD25 expression on day 3, representative histogram shown. D) Flow cytometric analysis of Granzyme B and CD62L evaluated +/- CAL-101, representative example shown (n=3). (E) ATAC-Seq analysis of day 3 activated sorted viable WT and *Pik3cd*$^{E1020K/+}$ OT-1 cells (n=2 independent experiments). *De novo* motif discovery using revealed KLF motifs enriched in WT cells. (F-G) ATAC-Seq analysis of sorted viable lung WT and *Pik3cd*$^{E1020K/+}$ OT-1 cells day 7 following influenza X31-OVA infection (n=2). E) Scatter plots of normalized ATAQ-Seq tag density compare chromatin accessibility (fold change>2; FDR<0.05). F) *De novo* motif discovery using HOMER revealed enrichment of BORIS, IRF4 and Tcf7 enriched motifs in WT cells (lower half). (H) Pie chart illustrating overlap of unique ATAC-Seq peaks from WT and *Pik3cd*$^{E1020K/+}$ day 3 activated CD8^{+} T cells with peaks from memory CD8^{+} T cells isolated from bone marrow.
